# Comparative Structural Analysis of Different Mycobacteriophage-Derived Mycolylarabinogalactan Esterases (Lysin B)

**DOI:** 10.3390/biom10010045

**Published:** 2019-12-27

**Authors:** Ahmed H. Korany, Adel Abouhmad, Walid Bakeer, Tamer Essam, Magdy A. Amin, Rajni Hatti-Kaul, Tarek Dishisha

**Affiliations:** 1 Department of Microbiology and Immunology, Faculty of Pharmacy, Nahda University, Beni-Suef 62513, Egypt; ahmed.hassan@nub.edu.eg (A.H.K.); waleed.ismail@nub.edu.eg (W.B.); 2 Biotechnology, Department of Chemistry, Center for Chemistry and Chemical Engineering, Lund University, P.O. Box 124, SE-221 00 Lund, Sweden; adel.abouhmad@biotek.lu.se; 3 Department of Microbiology and Immunology, Faculty of Pharmacy, Al-Azhar University, Assiut 71524, Egypt; 4 Department of Microbiology and Immunology, Faculty of Pharmacy, Beni-Suef University, Beni-Suef 26511, Egypt; tarek.dishisha@pharm.bsu.edu.eg; 5 Department of Microbiology and Immunology, Faculty of Pharmacy, Cairo University, Cairo 11562, Egypt; tamer.essam@yahoo.com (T.E.); magdy_biotek3@yahoo.com (M.A.A.)

**Keywords:** mycolylarabinogalactan esterases, α/β-hydrolase family, LysB homology models, multiple sequence alignment, molecular docking

## Abstract

Mycobacteriophage endolysins have emerged as a potential alternative to the current antimycobacterial agents. This study focuses on mycolylarabinogalactan hydrolase (LysB) enzymes of the α/β-hydrolase family, which disrupt the unique mycolic acid layer of mycobacterium cell wall. Multiple sequence alignment and structural analysis studies showed LysB-D29, the only enzyme with a solved three-dimensional structure, to share several common features with esterases (lacking lid domain) and lipases (acting on long chain lipids). Sequence and structural comparisons of 30 LysB homology models showed great variation in domain organizations and total protein length with major differences in the loop-5 motif harboring the catalytic histidine residue. Docking of different *p*-nitrophenyl ligands (C4-C18) to LysB-3D models revealed that the differences in length and residues of loop-5 contributed towards wide diversity of active site conformations (long tunnels, deep and superficial funnels, shallow bowls, and a narrow buried cave) resembling that of lipases, cutinases, and esterases. A set of seven LysB enzymes were recombinantly produced; their activity against *p*-nitrophenyl esters could be related to their active site conformation and acyl binding site. LysB-D29 (long tunnel) showed the highest activity with long chain *p*-nitrophenyl palmitate followed by LysB-Omega (shallow bowl) and LysB-Saal (deep funnel).

## 1. Introduction

Mycobacterial infections cause a number of deadly and disabling diseases worldwide, including tuberculosis (TB), leprosy, and buruli ulcers [1], the most common being TB, a respiratory contagious disease caused by a direct contact with the acid-fast bacterium, *Mycobacterium tuberculosis* [2]. Approximately 10 million of the world’s population fell ill with TB and around 1.3 million annual deaths were reported in 2017, making it the world’s second leading killer infectious diseases next to HIV [3]. Two new forms of TB infections, known as multidrug-resistant TB (MDR-TB) and extensively drug resistant TB (XDR-TB), have emerged, which are difficult and expensive to treat and fail to respond to standard antimycobacterials [4,5,6].

Although mycobacteria are classified as Gram-positive bacteria, the multilayered structure of their outer membrane (mycomembrane) shares common features with Gram-negative bacteria [7]. A characteristic feature of the cell wall is the presence of a unique type of long chain (C_60_–C_90_) fatty acids known as mycolic acids that are esterified to (i) the disaccharide sugar trehalose, forming trehalose 6,6-dimycolate (TDM) and trehalose-6-monomycolate (TMM) that constitute the outer layer of glycolipids (extractable lipids), and (ii) the polysaccharide layer arabinogalactan (AG), which is covalently linked to the inner peptidoglycan layer (PG) to form a rigid, highly hydrophobic mycolyl arabinogalactan-peptidoglycan (mAGP; non-extractable lipids) complex [8,9,10].

This highly intricate nature of the mycobacterial cell envelope poses a major obstacle for the passage of antibiotics into the mycobacterial cells for treatment of the disease [8,9,10]. Fortunately, mycobacteriophages, the viruses that specifically infect mycobacteria, produce different lytic enzymes (endolysins) that act synergistically to ensure complete cell lysis. Two mycobacterial endolysins, LysA and LysB, are known, the former targeting the PG layer and the latter acts on the mAGP complex hydrolyzing the ester bonds between mycolic acids and AG [11,12,13,14,15]. For treatment of mycobacterial cells from outside, LysB catalyzed hydrolysis of the mAGP layer is important for enabling access to LysA for lysis of PG [15,16].

Despite its importance, few reports so far have addressed the understanding of structure and function of LysB enzymes [11,13,16,17,18]. Among more than 1800 fully sequenced mycobacteriophages, only the 3D structure of LysB from mycobacteriophage D29 has been resolved to date [13]. Earlier reports on LysBs have revealed these enzymes to be a type of serine esterases that belong to the α/β hydrolase family, having highly variable sets of domains characteristic of cutinase and/or Pro-Glu (PE)- and Pro-Pro-Glu (PPE) motifs. Only three LysB enzymes (D29, Ms6, and Bxz2) have been screened for their enzymatic activity and have shown structural relatedness and activity patterns similar to esterases and lipases [11,12,13,17]. They resemble esterases in exhibiting activity against *para*-nitrophenyl butyrate (*p*-NPB) [13] and tributyrin [11]. In addition, LysBs have similar activity pattern to cutinases and lipases on different lipids [11], beside their exclusive action on mAGP that is lacking in all other α/β-hydrolases [13].

The aim of the present study was to perform comparative bioinformatic analysis to gain insight to the sequence and structural diversity of LysB enzymes and to get a clear idea on the relationship with its relative α/β hydrolases (esterases, cutinases, and lipases). Thirty LysB enzymes having statistically significant sequence similarity (>30% identity to LysB-D29) were chosen and subjected to molecular homology modelling and docking of *p*-nitrophenyl ligands of different chain lengths to understand the interaction between the ligands and the active site. The information gained was further supported by running enzymatic activity assays of seven LysB enzymes on different *p*-nitrophenyl substrates (C12 and C16).

## 2. Materials and Methods

### 2.1. Multiple Sequence Alignment and Phylogenetic Analysis

Homologous sequences for LysB-D29 were identified using the Position Specific Iterated-Basic Local Alignment Search Tool (PSI-BLAST) against the UniProt database [19], and analyzed using the Protein family database (PFAM) through the MOTIF online tool with default settings [20]. Multiple sequence alignments of LysB-D29 with selected members of α/β hydrolase family or homologous putative LysB proteins were done using Clustal Omega embedded in the UniProt database [21]. Phylogenetic tree was constructed using maximum likelihood in Molecular Evolutionary Genetics Analysis (MEGA) 6.0 software [22].

### 2.2. Homology Modeling and Structural Alignments of LysB Homologs

Homology modeling of selected LysB sequences was done based on known templates having similar function, using the automated YASARA^®^ Structures software package version 17.4.17 (YASARA Biosciences GmbH, Graz, Austria) [23,24]. The modeling parameters were set to, speed: slow (slow = best), maximum number of templates to be used: 5, and maximum number of conformations tried per loop (LoopSamples): 50.

Briefly, the sequences were subjected to PSI-protein blast against th eProtein Data Bank (PDB) [25] and several hits were retrieved and classified according to the total score (simply the product of the BLAST alignment score, the WHAT_CHECK quality score in the PDBFinder2 database and the target coverage). The five templates with the highest total score were used for model building. For each template, YASARA^®^ constructed different initial models that were subjected to two successive simulated annealing minimizations for the side chains and the entire models, respectively. After each refinement step, a Z-score (measure of the standard deviation of the model quality from the average high-resolution X-ray structure) was calculated. Subsequently, all the generated models from the five templates were ranked according to their Z-score. Finally, the fractions with the best scoring from all the models were fused to obtain a number of hybrid models, aiming to increase the accuracy beyond each of the contributing initial models and, thus, capturing the correctness of backbone-(Ramachandran plot) and side-chain dihedrals, as well as packing interactions. The resulting hybrid model was given an overall Z-score score (Table S1).

The models were further evaluated using Verify_3D [26], PROCHECK [27], ERRAT (Protein structure verification web server) [28], Prove [29] and ProSA (Protein Structure Analysis) [30] (Table S1).

In order to get a crystallized protein structure of the relative members of the α/β hydrolase family bound with inhibitors (holo-protein), we searched the structural database using PDB ID: 3HC7 as query structure for Dali server [31]. The hits of similar esterases, cutinases, and lipases complexed with inhibitors were selected. Structure-based sequence alignments of LysB-D29 with its relative α/β hydrolase members and other homologous LysB models were performed using UCSF Chimera version 1.13.1 [32]. Structural alignments of the active sites of LysB-D29 and the 30 homologous LysB models were done using YASARA^®^ Structures (version 17.4.17, YASARA Biosciences GmbH, Graz, Austria).

### 2.3. Molecular Docking of Various Substrates against Generated LysB Models

In order to compare the potential affinity of the LysB-D29 and its 3D homology models towards *p*-nitrophenyl (*p*-NP) ligands (C4-C18), molecular docking studies were carried out using Molecular Operating Environment (MOE^®^ 2014.0901, Chemical Computing Group, Montreal, QC, Canada) as the computational software [33]. Before docking, ligands and proteins were prepared using MOE^®^ 2014.0901 software. Most macromolecular crystal structures contain little or no hydrogen coordinate data due to limited resolution and thus the constructed 3D models were subjected to protonation prior to docking using Protonate 3D tools implemented in MOE^®^. Protonation was followed by energy minimization of all 3D models up to 0.05 Gradient using Amber99 force field. MOE^®^ Alpha Site Finder was used for search of the active sites in the enzyme structure and dummy atoms were created from the obtained alpha spheres. MOE script (sitefind_volume.svl) was used to measure the size, volume, and solvent accessible surface area (SASA) of each active site.

3D structures of all substrate compounds (*p*-NP ligands) were built by means of the Molecular Builder program implemented in MOE^®^ software. Then, a database was created in which all the ligands were converted into their particular 3D structures and this database was used as input file for MOE^®^-docking. Subsequently, the energy of compounds present in the database was minimized up to a 0.05 gradient using an MMFF94x force field. The database was then docked into the active site of each of the LysB protein models using the induced fit docking method [34,35] and conformations of each ligand-protein complex were generated with a docking score (S). Each complex was analyzed for interaction of the ligand with the protein active site and their 3D pose was taken. The best pose of the docked ligand was selected based on minimum Gibbs free energy (docking score (S)).

### 2.4. Cloning of LysB-His_6_ Enzymes

*LysB* genes from LysB candidate models, with a hexa-histidine tag (His_6_) at the C-terminus, were codon optimized and ordered as gBlock gene fragments from Integrated DNA Technologies (IDT, Leuven, Belgium). *LysB-His*_6_ gBlocks were cloned in *EcoR*I and *Nde*I restriction sites of pET22b (+) expression vector (Novagen, Madison, WI, USA). After ligation with T4 DNA ligase (Thermo Fisher Scientific, Waltham, MA, USA), the ligation mixture was transformed into *E. coli* BL21(DE3) (Novagen, Madison, WI, USA), plated on LB agar (Saveen and Werner AB, Limhamn, Sweden) supplemented with 100 µg/mL ampicillin (Sigma-Aldrich, St Louis, MO, USA), and grown overnight at 37 °C. Plasmids (pET22b(+)-LysB-His_6_) were extracted from the transformant colonies and sequenced (GATC Biotech AB, Solna, Sweden); the one with the correct sequence was used for protein expression in *E. coli* BL21(DE3). Cells were grown overnight at 37 °C, 200 rpm in LB supplemented with 100 µg/mL ampicillin, and glycerol stocks of the recombinant cells were prepared and stored at −80 °C.

### 2.5. Production and Purification of LysB-His_6_ Enzymes

#### 2.5.1. Small-Scale Expression

For inocula preparation, the respective glycerol stocks were inoculated into 10 mL LB medium with 100 μg/mL ampicillin and grown overnight at 37 °C, 200 rpm in 50 mL sterile falcon tubes. Subsequently, 5 mL of the culture were used to inoculate 50 mL of the same medium in 250 mL Erlenmeyer flask grown under similar conditions as above. When the optical density (OD600_nm_) reached 0.5–0.6, 1mM isopropyl β-d-1-thiogalactopyranoside (IPTG; Thermo Fisher Scientific, Waltham, MA, USA) was added to induce the protein expression and the incubation temperature was decreased to 30 °C. After 4 h, the cells were harvested by centrifugation (3900× *g*, 4 °C, 15 min; Sigma 3-16PK).

#### 2.5.2. Large-Scale Protein Expression

Large scale protein expression was performed in auto-induction medium (1% tryptone, 0.5%, yeast extract, 25 mM Na_2_HPO_4_, 25 mM KH_2_PO_4_, 25 mM (NH_4_)_2_SO_4_, 2 mM MgSO_4_, 0.05% glucose, and 0.2% α-lactose) supplemented with 100 µg/mL ampicillin. The inocula were prepared in LB medium as above, 15 mL inocula were used to inoculate 1 L of auto-induction medium, cells were allowed to grow at 37 °C, 180 rpm for 4 h, followed by cultivation for 24 h at 30 °C prior to harvesting the cells (6000× *g*, 20 min, 4 °C; Sorvall Lynx 4000 centrifuge, Thermo Scientific, Waltham, MA, USA).

### 2.6. Purification of LysB-His_6_ Enzymes

The cell pellet was suspended in a resuspension buffer (50 mM Tris-HCl, 50 mM NaCl, pH 8) supplemented with a cocktail of protease inhibitors (Calbiochem) and then sonicated (5 × 60 s, cycle 0.5) on ice using UP400S sonicator (Dr. Hielscher GmbH, Stahnsdorf, Germany). The cell debris was removed by centrifugation (18,500× *g*, 30 min, 4 °C; Sorvall RC5C, Sorvall Instruments, Dupont, Wilmington, DE). The soluble recombinant proteins were purified from the clarified lysate by metal ion affinity chromatography (IMAC) on 5 mL HisTrap FF^TM^ nickel column (GE Healthcare Bio-Sciences AB, Uppsala, Sweden) according to manufacturer’s instructions. The column was equilibrated with the binding buffer (50 mM Tris-HCl, 0.3 M NaCl, 20 mM imidazole, pH 8) before applying the clarified lysate, unbound proteins were washed out with wash buffer (50 mM Tris-HCl, 0.3 M NaCl, 40 mM imidazole, pH 8) and, finally, the bound proteins were eluted with elution buffer (50 mM Tris-HCl, 0.3 M NaCl, 0.5 M imidazole, pH 8). The purified proteins were dialyzed against dialysis buffer (50 mM Tris-HCl, 50 mM NaCl, 50% *w*/*v* glycerol, pH 8), analyzed by SDS-PAGE, quantified with Bicinchoninic acid (BCA) using bovine serum albumin (BSA) as a standard (Sigma-Aldrich, St Louis, MO, USA) and stored at −20 °C.

### 2.7. Assay of Enzymatic Activity

Activity measurement of LysB-His_6_ enzymes was based on following the formation of *para*-nitrophenol spectrophotometrically at 410 nm, resulting from the hydrolysis of *p*-nitrophenyl palmitate (*p*-NPP) and *p*-nitrophenyl laurate (*p*-NPL), respectively, by LysB-His_6_ enzymes. The assays were done using a microtiter plate reader with built-in incubator (Multiskan^TM^ GO Microplate Spectrophotometer, Thermo Scientific, Waltham, MA, USA). Twenty microliters of LysB-His_6_ enzymes were added to 180 µL of *p*-NPL and *p*-NPP, respectively (1 mM dissolved in 20 mM Tris-HCl, 100 mM NaCl, 0.1% Triton X-100, pH 8), the reaction mixture was incubated at 37 °C and the release of *p*-nitrophenol was recorded at 410 nm at 1 min intervals. Reactions without LysB enzymes were run as blanks. The assay was done in 3 independent replicates and the presented data are the mean of these replicates ±standard deviation.

For calculating the enzymatic activity, the following equation was used:

$$\mathrm{Activity}\;\left(\mathrm{Unit}/\mathrm{mL}\;\mathrm{enzyme}\right)\;=\;\frac{\left(\Delta A_{410}\;\mathrm{test}-\Delta A_{410}\;\mathrm{blank}\right)\times \left(\mathrm{TV}\right)\;}{\varepsilon \times \left(V\right)}$$

where:

TV: is the total reaction volume in milliliters (0.2 mL)
ε: is the extinction coefficient of *p*-nitrophenol (8.4 mM^−1^)
V: is the volume in milliliters of the enzyme solution added to the reaction (0.02 mL).

One unit (U) of enzyme activity corresponds to the liberation of 1 µmol of *p*-nitrophenol per min under the assay conditions.

For measuring the specific activity, the following equation was used:

$$\mathrm{Specific}\;\mathrm{activity}\;\left(U/\mathrm{mg}\;\mathrm{enzyme}\right)\;=\;\frac{\mathrm{Activity}\;\left(U/\mathrm{mL}\right)}{\mathrm{Enzyme}\;\mathrm{concentration}\;\left(\mathrm{mg}/\mathrm{mL}\right)}$$

## 3. Results

The comparative study of LysB-D29 with the relative members of α/β hydrolase family and with the 30 homologous LysB proteins, respectively, consisted of four steps: (1) multiple sequence alignment; (2) generating homology models for selected LysBs and comparison with the resolved 3D structure of LysB-D29 available in protein databank (PDB ID: 3HC7); (3) molecular docking studies of different *p*-nitrophenyl ligands to the active site of 3D structure of the selected α/β hydrolase family members and LysB models; and (4) determination of the specific activity of LysB-His_6_ enzymes against *p*-NPL and *p*-NPP, respectively.

### 3.1. Comparison of LysB-D29 to Different α/β Hydrolase Family Members

#### 3.1.1. Multiple Sequence Alignment and Phylogenetic Analysis

Multiple sequence alignment of LysB-D29 and relative members of the α/β hydrolase family was done in order to identify common features and differences. LysB-D29 protein has the same length of 254 residues as *Trichoderma reesei* cutinase, which is intermediate between the shorter cutinases and esterases, *Humicola insolens* cutinase (194 residues), *Fusarium solani* cutinase (230 residues) and *Penicillium purpureogenum* acetylxylan esterase (234 residues), and the longer lipases, *Pseudomonas cepacia* lipase (364 residues), human pancreatic lipase (465 residues) and *Candida rugosa* lipase (549 residues) (Table 1).

All proteins share high conservation of the classic catalytic triad residues (Ser, His, and Asp (Glu for *C. rugosa* lipase)) and the pentapeptide GXSXG (typical GYSQG for LysB-D29, *F. solani* cutinase, *H. insolens* cutinase, *T. reesei* cutinase, and *P. purpureogenum* acetylxylan esterase) (Figure S1). Except for the true cutinases from *F. solani* and *H. insolens*, the GXP motif seems to be highly conserved in all other proteins. Inversely, many gaps were identified among amino acid sequences of LysB-D29 compared to its relative α/β hydrolase members that contributed to the major differences (Figures S1 and S2).

#### 3.1.2. Structural Comparison of LysB-D29 with Different α/β Hydrolase Family Members

The crystal structure of LysB-D29 was used as query in Dali server to search for similar α/β hydrolase members. Thousands of hits were obtained, but according to the highest percentage of identity and presence of co-crystallized inhibitor, seven proteins were selected for structural comparison (Table 1). The retrieved structures in complex with inhibitors were chosen to allow the proteins to be in their open conformation (especially for lipases which have a lid domain).

The multiple sequence- and structural- alignments revealed that LysB-D29 has common features with esterases, cutinases, and lipases. LysB-D29 has a typical α/β fold (consisting of five central parallel β-strands winged by two α-helices on each side) similar to esterases and cutinases (Figure S3), however, it lacks the first two short N-terminal α-helices (region-2 in the multiple sequence alignment) found in all cutinases (Figure S1). The positions of the catalytic triad residues are also highly conserved among LysB-D29, *P. purpureogenum* acetylxylan esterase and all cutinases.

**Figure 1:**
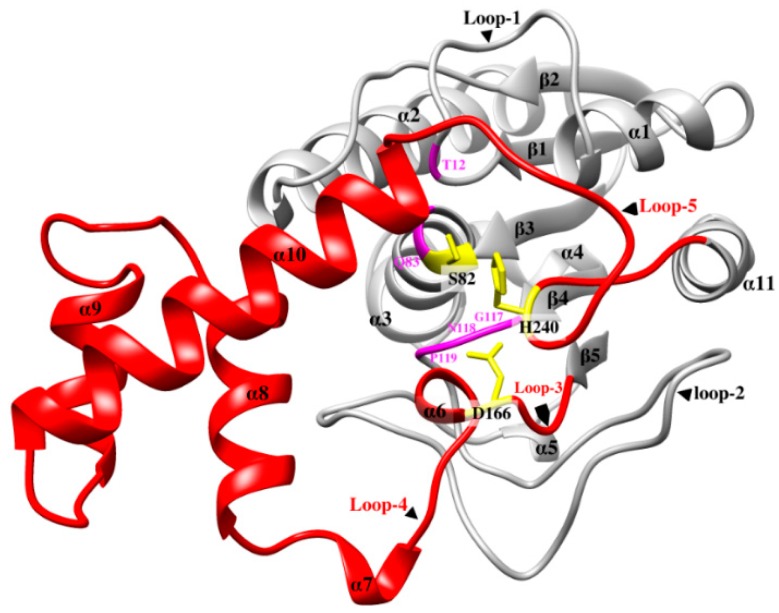
Crystal structure of LysB-D29 showing secondary structure elements including linker domain (red color), catalytic triad residues (yellow), oxyanion hole residues, and GNP residues (pink), and the rest of the protein (gray).

LysB-D29 possesses an 83-residues-long domain (region-3 in multiple sequence alignment) (Figure S1) linking the end of the fifth β-strand (Y161) to the beginning of the C-terminal α-helix (Y245) (Figure 1). This long “linker” domain also connects the members of the catalytic triad (D166 to H240). It was noticed that the distance between these two catalytic residues in LysB-D29 is much longer than that in other esterases and cutinases (12 residues) (Figures S1 and S3). Furthermore, *T. reesei* cutinase has an additional N-terminal domain (region-1 in multiple sequence alignment), which aligns well to the linker domain of LysB-D29 and is missing in *P. purpureogenum* acetylxylan esterase and the other two cutinases (*F. solani* cutinase and *H. insolens* cutinase) (Figures S1 and S3).

LysB-D29 exhibited lower sequence identity to lipases than esterases and cutinases (Table 1). LysB-D29 is shorter by 100–300 residues than the aligned lipases, which can be attributed to the larger number of parallel β-strands of the central α/β fold in lipases (6, 8, and 10 for *P. cepacia* lipase, human pancreatic lipase and *C. rugosa* lipase, respectively, compared to five in LysB-D29) that appeared as gaps in LysB-D29 in the multiple sequence alignment (Figure S2). Moreover, lipases have lid domains (region 1 and 2) covering the active site when in closed form, which is a missing feature in LysB-D29 (Figures S2 and S4).

On the other hand, LysB-D29 shares high conservation of the GXP motif with lipases where X accounts for Asn in LysB-D29, Lys in *C. rugosa* lipase and Thr in both *P. cepacia* lipase and human pancreatic lipase (Figure S2). However, the position of this motif is poorly conserved in *C. rugosa* lipase and human pancreatic lipase in comparison to LysB-D29, *P. cepacia* lipase, *P. purpureogenum* acetylxylan esterase, and *Trichoderma reesei* cutinase, where this motif is located at the end of the forth β-strand just adjacent to the catalytic serine (34, 22, 40, and 26 residues, respectively, downstream of the catalytic Ser) (Figures S3 and S4b–f). The long sequence connecting the catalytic Asp and His that corresponds to the linker domain in LysB-D29 (83 residues) was found to be comparable to that of human pancreatic lipase and *C. rugosa* lipase (87 and 107, respectively) and much longer than *P. cepacia* lipase (21 residues) (Figure S2).

### 3.2. Comparison of LysB-D29 to Its Homologous LysB Models

#### 3.2.1. Multiple Sequence Alignment and Phylogenetic Analysis

The selection of LysB sequences for comparative bioinformatics analysis was based on the degree of sequence identity to LysB-D29 [13]. The search for LysB-D29 homologous proteins was done using BLAST search against the UniProt database which provided 1000 hits. The different homologous proteins were classified into seven groups according to their percentage identity to LysB-D29 and their chain length (Table 2). The identity ranged from 100% to 23.4%, and homologous LysB proteins with percentage similarity of more than 30% (statistically significant sequence similarity) only were selected for subsequent comparative study [36,37].

Thirty homologous proteins were selected as representatives for each of the seven groups (Table S2). Multiple alignments of amino acid sequences revealed a number of important features: (i) LysB proteins vary in length from 244 (LysB-BabyRay group10) to 346 (LysB-Dylan gp67) residues. (ii) The domain architectures of the represented LysB proteins were highly diverse ranging from no conserved motif (LysB-Obama12 and -Enkosi) to having up to seven different motifs (LysB-MrMagoo). The majority of proteins (25 LysBs besides LysB-D29) have two combined domains: PE-PPE (PF08237) and cutinase (PF01083), among them 15 LysB proteins have one, two or four additional motifs (Table S2). Three LysB proteins have a sole domain, either PE-PPE (LysB-Palestino) or cutinase -motif (LysB-Omega and -Larva). (iii) With respect to conserved residues, serine and aspartate residues from the catalytic triad are absolutely conserved in contrast to the third member (histidine) which has weak conservation (Figure S5b–d). Additionally, the pentapeptide G[DA]-Y[F]-S-Q-G[S] and the GNP motifs are highly conserved among all members of the seven groups. Remarkably, two regions were found to be highly variable among all LysB sequences representing the different N-terminal extra residues (region-1) and C-terminal mobile loop (region-2) (Figure S5a,d).

**Figure 2:**
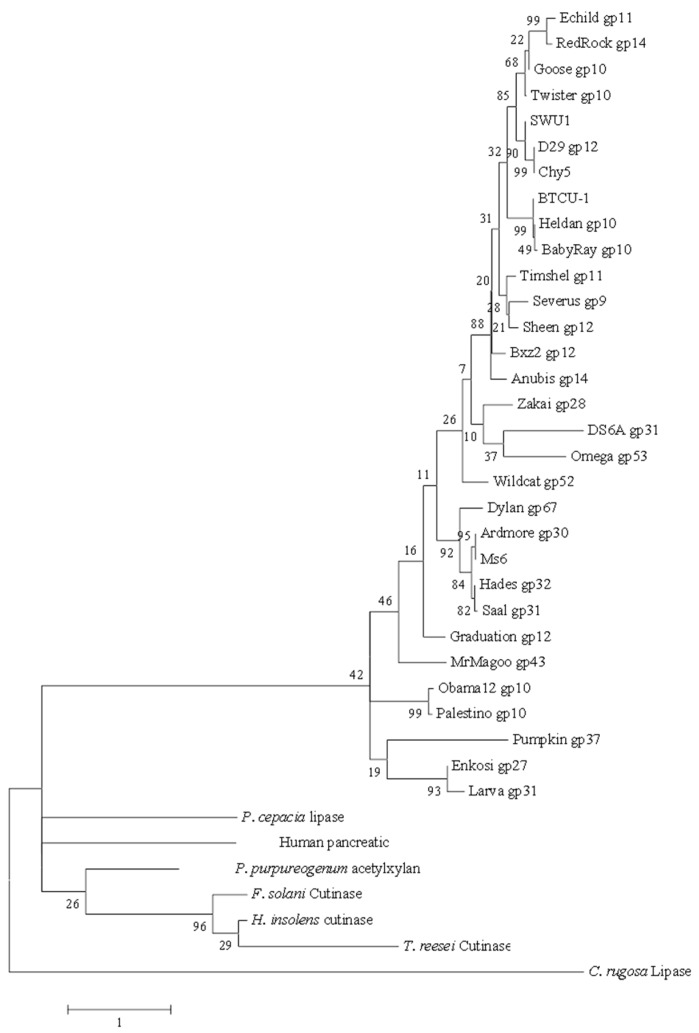
Phylogenetic tree for LysB proteins of mycobacteriophage D29 and its 30 homologs, and relative members of the α/β hydrolase family. The tree was built using maximum likelihood (ML) method by MEGA 6.0. The bootstrap values are shown at every node. The high bootstrap value at each node reveals the significance of its branching.

To explore the phylogenetic relationship among the LysB-D29 homologs and relative members of the α/β hydrolase family, we constructed an unrooted maximum likelihood (ML) tree (Figure 2). In general, LysB representatives of the same group share the same root in the phylogenetic tree except for DS6A (gp 6A) and Omega (gp 7C), which inspite of different degrees of identity to LysB-D29, share the same root. It is obvious that almost all LysB enzymes are distant from esterases, cutinases, and lipases.

#### 3.2.2. Structural Comparison of LysB-D29 to Homologous LysB Models

In the absence of experimentally solved structures, an attempt was made to predict the three-dimensional (3D) structure for LysB-D29 homologs in order to gain information on their structure and active site conformation. YASARA^®^ Structures, a powerful tool for generation of refined homology models from high-resolution crystallographic structures, was used for generating the 3D models [24]. Almost all the predicted 3D models of the proteins were shown to have a good geometrical quality comparable to 3D structure of LysB-D29 as confirmed by the Z-score, Verify-3D, ERRAT quality factor, Prove, and Ramachandran plot (Table S1 and Figure S6). Nevertheless, the accuracy of the information obtained with the homology models would need to be confirmed with crystal structures of the proteins.

Structural alignments of LysB-D29 and the 3D homology models revealed almost identical pattern of their secondary structures except for a fourteen-residue long loop (loop-5) in the linker domain extending from R231 to R244 in LysB-D29 and connecting the rest of linker domain to the C-terminal helix (Figure 1). This mobile loop is a hypervariable region among all LysB representatives in terms of length and the involved amino acid residues (Table S3 and Figure S5d) and contains the catalytic His residue. Furthermore, major differences in four other loops (loop-1, -2, -3, and -4) appear mainly in members of the sixth and the seventh group with low percentage identities (43–30%) to LysB-D29 (Figure S7). As the flexible loops may adopt different conformations, their role may be related to influencing substrate entry, proper substrate positioning and controlling of the reaction conditions (see also Section 3.3.2).

**Figure 3:**
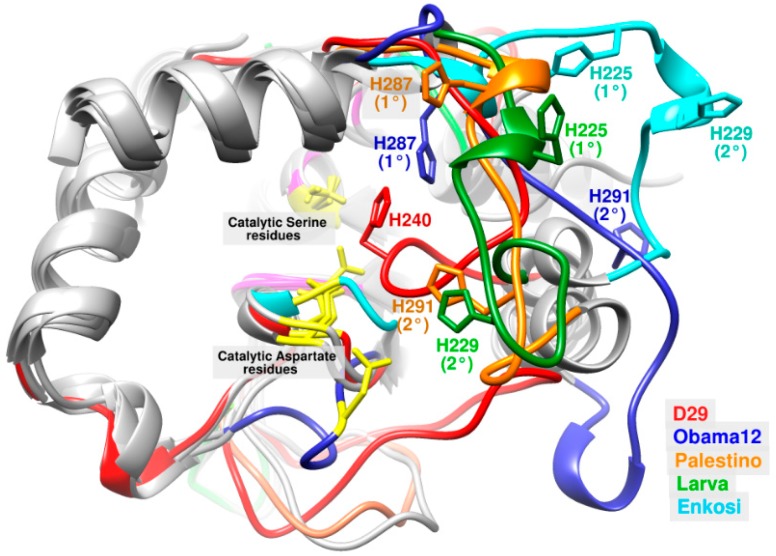
3D structural alignment of LysB-D29 (red) with LysB-Obama12 (blue), LysB-Palestino (orange), LysB-Larva (green) and LysB-Enkosi (cyan) models with focus on loop-5. The figure shows catalytic Ser and Asp residues (yellow), primary and additional His residues (in each protein color), oxyanion hole, and GNP residues (pink), and the rest of the protein (gray).

Interestingly, the multiple sequence alignment showed poor conservation of His residue through subtle shifts either N-terminally by one position (e.g., LysB-Wildcat) or C-terminally by three positions (LysB-Pumpkin) (Figure S5d). However, structural alignment illustrated great translocation of the catalytic His in many LysB models from its aligned position in LysB-D29 and far away from the two other catalytic residues (Ser and Asp) whose positions were well conserved (Figure S7). Additionally, LysB models with similarity percentage to LysB-D29 below 36% (group 7B and 7C except LysB-Omega) showed an additional His residue (His 291 in LysB-Palestino and -Obama12 and His 229 in LysB-Larva and -Enkosi) located three positions towards the C-terminus compared to that of the primary catalytic His 287 and 225, respectively (Figures S5d and S7k,l). This secondary His residue was adjacent to the catalytic Ser and Asp (i.e., in a comparable position to that of His residue in LysB-D29) in both LysB-Palestino and LysB-Larva models in contrast to LysB-Obama12 and LysB-Enkosi (Figure 3 and Figure S7k,l).

Furthermore, significant difference in the length of the mobile loop-5 was observed in the 3D structural alignment and the multiple sequence alignment. For example, the multiple sequence alignment of LysB-Enkosi and LysB-Obama12 proteins to LysB-D29 showed a decrease in length of loop-5 by one and three residues, respectively. However, structural alignment of the 3D models of the same proteins revealed a notable increase in loop-5 length by two (LysB-Enkosi) and five (LysB-Obama12) residues, respectively, with respect to the crystal structure of Lys-D29 (Table S3 and Figure 3).

Structural alignments revealed an absolutely conserved position of GNP motif between the main catalytic serine and aspartate residues in all the studied LysB models (Figure S6).

### 3.3. Molecular Docking Studies of the Generated LysB Homology Models

#### 3.3.1. Substrate Affinity of *p*-Nitrophenyl Esters to LysB Models

Molecular docking studies were performed in order to understand the likely interactions of the *p*-nitrophenyl ligands (C4–C18) with the active sites of the constructed LysB homology models and to deduce their potential enzymatic activity on the basis of affinity to the different ligands.

**Figure 4:**
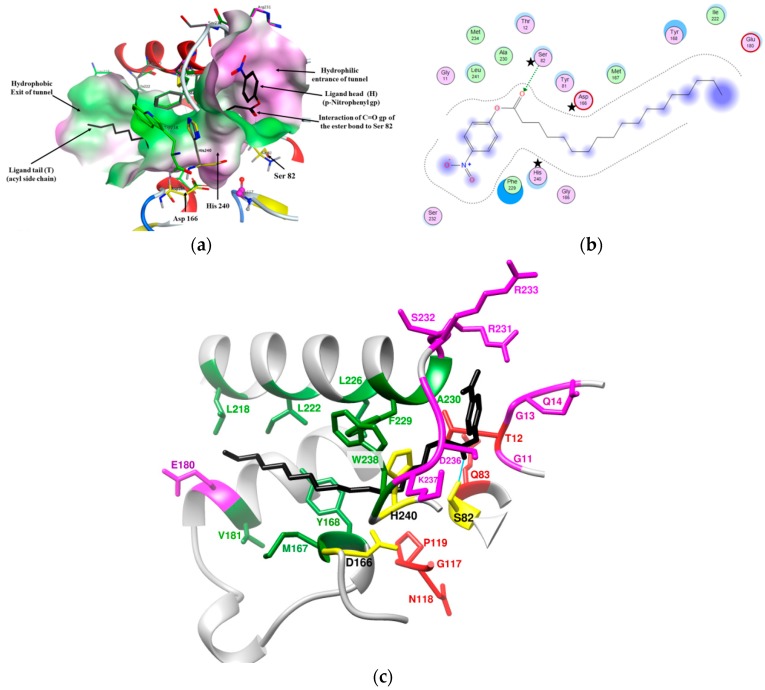
Binding of *p*-NPS (C18) (black color) to the tunnel binding site of LysB-D29 showing: (**a**) 3D shape of LysB-D29 active site, (**b**) 2D illustration of interacting residues of LysB-D29 active site with *p*-NPS ligand (generated by MOE^®^) (stars indicate catalytic triad residues of LysB-D29), (**c**) 3D illustration of interacting residues of LysB-D29 active site with *p*-NPS ligand: loops forming the hydrophilic opening (Loop-1 (G11 to Q14) and loop-5 (R231 to K237) in pink), helices forming the hydrophobic opening (α6 (M167 and Y168), α8 (V181), α10 (L218, I222, A225, L226, F229, A230), and loop-5 (W238) in green), oxyanion hole residues (T12 and Q83) and GNP residues (red), and H-bond between serine residue of LysB-D29 and the carbonyl group (C=O) of *p*-NPS ligand (cyan).

The space in the constructed LysB models containing the catalytic triad Ser, Asp, and His (counterparts to no. 82, 166, and 240; respectively in LysB-D29) was selected as the docking site as reported earlier [13], where Ser residue plays the key role of attacking the ester bond of each ligand [38]. Upon docking of *p*-nitrophenyl ligands to the active site of LysB-D29 crystal structure, several poses (docking configurations of the ligand-enzyme complexes) were obtained. Computationally, poses achieving hydrogen bond interaction between the carbonyl group of the ligand ester bond and Ser82 residue (Figure 4) and having the highest docking score (S) were selected as the top ranked conformations.

Table S3 represents the top poses (with docking scores) achieved upon docking different *p*-nitrophenyl ligands to the 30 LysB models. For example, LysB-D29 showed better S values of −7.56 and −8.07 kcal/mol for poses 136 and 163 of long chain *p*-nitrophenyl ligands *p*-NPP and *p*-NPS, respectively, than S values −5.41 and −6.45 for poses 4 and 22, respectively, for shorter chain *p*-nitrophenyl ligands *p*-NPB and *p*-NPC.

#### 3.3.2. Active Site Conformation

Based on the docking results, it was clear that the active site of LysB-D29 has a characteristic long L-shaped tunnel conformation wherein the head of the ligand (*p*-nitrophenyl group) is directed towards the hydrophilic mouth of the tunnel formed by G11 to Q14 (loop-1) and R231 to K237 (loop-5). The tail of the ligand (acyl side chain) is accepted by the hydrophobic body of the tunnel lined by F229, A230, W238, and Y168 (linker α10, loop-5, and α6) and then directed towards the hydrophobic exit of the tunnel formed by M167, V181, and L218, I222, A225, L226 (linker α6, α8, and α10) (Figure 4c). This tunnel conformation of the active site is a common feature for more than half of the studied LysB models (16 out of 30), (Table S3 and Figure S8-IA) and might explain the higher affinity of this group of enzymes towards longer chain *p*-nitrophenyl substrates.

Interestingly, apart from LysB-Chy5, we noticed a remarkable difference in the number and type of hydrophilic residues of loop-5 (forming the hydrophilic mouth of the tunnel) among the tunnel-shaped LysB active sites. For instance, Loop-5 of LysB-D29 and -Chy5 involve more polar hydrophilic residues (e.g., Arg, Asp and Lys) than the tunnels in the other LysB proteins (Figure S8d). While, exit of the tunnels in the latter LysB proteins is more hydrophilic than that of LysB-D29 and -Chy5 tunnels (Figure S8-IA,B). These differences might explain the observed low activities (Table S3) and the inverted orientation of the *p*-NP ligands obtained with docking experiments (Figure S8-IB). In these cases, the hydrophilic head of *p*-NP ligands was oriented towards the exit (instead of the mouth of the tunnel) and the acyl side chain towards the mouth of the tunnel where sufficient hydrophobic surface is available (Figure S8-IB). These inverted orientations had better docking scores compared to the corresponding un-inverted ones (the higher the negative value, the better the docking score).

The second conformation was deep funnel shape appearing in two forms (Figure S8-II). The first form is a very deep funnel which was noticed in LysB-Zakai, -Saal, -Graduation, and -DS6A models, in which the head of the ligand is buried in the deep hydrophilic bottom formed by the catalytic Ser and the two oxyanion hole residues Thr and Gln. However, the long acyl side chain twists sharply and follows the opposite hydrophobic wall of the funnel that is formed mainly by the hydrophobic residues of α6, α8 and α10. This hydrophobic wall wraps the acyl side chain terminating the deep funnel in a narrow exit near the protein surface (Figure S8-IIA). LysB protein models with the deep funnel form scored increasing (S) values with increasing chain length of their docked *p*-nitrophenyl ligands (Table S3). The second form is a less deep funnel where the hydrophilic head binds to the catalytic Ser residue in a shallow hydrophilic bottom while the acyl side chain curls sharply to follow a wide hydrophobic wall away towards the protein surface (Figure S8-IIB). Three LysB models including LysB-Twister, -Wildcat and -Hades showed this conformation.

The third conformation is a superficial funnel where the head of the ligand is almost flat, and the fatty acid side chain runs parallel to the short opposite hydrophobic wall where it exits the funnel. The bottom of this funnel is mainly lined by hydrophilic residues of the catalytic Ser and the two oxyanion hole residues and harbors the hydrophilic head of the ligand, while the hydrophobic tail of the ligand is faced by a short hydrophobic wall usually formed of α6, α8, and α10 (Figure S8-III). Five LysB models including LysB-Anubis, -Heldan, -Bxz2, -BTCU-1, and -Severus showed this conformation. Many of these models failed to achieve good poses with long chain *p*-nitrophenyl ligands, hence we expect a better activity with short chain substrates (Table S3).

An additional shallow conformation was noticed only in LysB-Omega, which represented a distinctive superficial bowl-shaped active site. In this case, an opened, shallow, long hydrophobic groove runs parallel to the surface of the LysB-Omega protein (Figure S8-IV). The hydrophilic head of the *p*-nitrophenyl ligand is directed towards a wide shallow hydrophilic pocket formed by the hydrophilic residues of loops 2 and 5 in addition to the catalytic Ser and the two oxyanion hole residues Thr and Gln. The hydrophobic tail curls at the bottom of this pocket and runs in a long hydrophobic furrow lined by the hydrophobic residues of α6, α8, and α10.

The last conformation was noted for the LysB-Goose model where its active site showed a narrow, buried cave. The head of the ligand is directed towards the catalytic Ser which is deeply buried in the protein core, and the tail of the ligand passes through a narrow hydrophobic channel that opens at the protein surface at C8 (Figure S8-V). This narrow channel hinders access to the catalytic Ser, which was reflected by lack of good docking scores/poses upon docking of different *p*-NP ligands (Table S3).

The active sites of the generated LysB models were further subjected to structural alignments with their relative crystallographic resolved α/β hydrolases with the same active site conformation (i.e., tunnel-shaped active sites of LysB models were aligned to the active site of LysB-D29 crystal structure, deep funnel-shaped active sites were aligned to the active sites of *C. antartica* lipase B and *P. cepacia* lipase crystal structures, etc.). The alignments were measured in terms of RMSD values, percentage of residues identity between aligned active sites, and the TM-score, a scoring function to assess the similarity of protein structures, was calculated for the aligned active sites and summarized in Table S4. Among the different proteins with the tunnel-shaped active sites, only LysB-Chy5 and -SWU-1 had about the same fold (TM-score > 0.5) to the corresponding reference protein (LysB-D29) with 100% sequence identity of their active sites. All other LysBs had TM-score below 0.17 to the corresponding reference proteins indicating random structural similarity. Moreover, Most LysB tunnels had more solvent accessible surface area than *C. rugosa* lipase tunnel. On the other hand, all shallow conformations demonstrate smaller volume and provide less accessible surface areas than deep and long active site conformations.

Interestingly, the GNP residues visualized by MOE^®^ were involved in forming the active site of almost all of the 30 LysB models (Figure 3 and Table S3). In addition, H-bond formation was observed between the glycine residue of the GNP motif and the catalytic Asp residues in the active site of LysB-Hades and -Ms6 (Figure S9f,g). Similar H-bonds were observed linking the catalytic Ser, Gly (residue of GNP motif) and the catalytic His in the active sites of *F. solani* cutinase, *P. cepacia* lipase, *R. miehei* lipase, *P. purpureogenum* acetylxylan esterase and *T. reesei* cutinase (Figure S9a–e).

Based on the results of this bioinformatics study, LysB candidate proteins representing all classes of LysB active site shapes as well as LysB-D29 (reference standard), were selected for cloning, protein expression, purification and subsequent enzymatic activity characterization (Table S3). However, only seven LysB proteins (LysB-D29, -SWU1, -Babyray, -Palestino, -Obama12, -Saal, -Omega), were successfully expressed and then tested for their enzymatic activity.

### 3.4. Enzymatic Activity

The enzymatic activity of the selected LysBs was determined against medium and long chain *p*-NP substrates: *p*-NPL (C12) and *p*-NPP (C16), respectively. LysB-Omega showed the highest specific activity with *p*-NPL (9.7 U/mg) while LysB-D29 had the highest specific activity against *p*-NPP (0.55 U/mg). LysB-Palestino had the lowest specific activity values (0.011 U/mg with *p*-NPL and no detectable activity with *p*-NPP) (Table 3). In general, the specific activity of the tested LysB proteins dropped dramatically by 84–97% with increase in the chain length of the *p*-NP substrates from C12 to C16. Moreover, all LysB enzymes showed the same relative activity to LysB-D29 with both *p*-NP substrates except for LysB-Omega whose specific activity with *p*-NPL was twice that of LysB-D29 and dramatically fell to only half the specific activity of LysB-D29 with *p*-NPP (Table 3). Interestingly, LysB-SWU1, and -Babyray had very low specific activity values with *p*-NPL while there was no detectable activity for LysB-Palestino with *p*-NPP.

**Figure 5:**
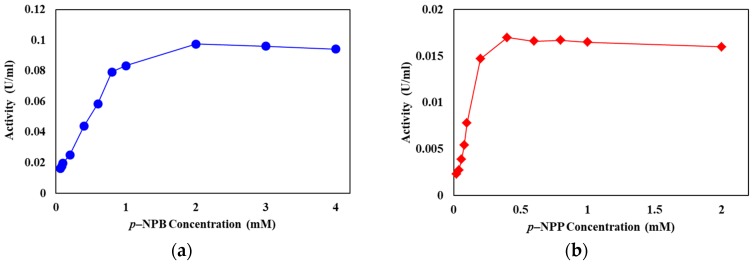
Rate of LysB-D29 catalyzed reaction with different concentrations of (**a**) *p*-NPB and (**b**) *p*-NPP. Twenty microliters of LysB-His_6_ enzymes were added to 180 µL of *p*-NPL and *p*-NPP, respectively (1 mM dissolved in 20 mM Tris-HCl, 100 mM NaCl, 0.1% Triton X-100, pH 8), the reaction mixture was incubated at 37 °C and the release of *p*-nitrophenol was recorded at 410 nm at 1 min intervals.

The effect of the surfactant Triton X-100 on the hydrolysis of *p*-NPB, *p*-NPL, and *p*-NPP by LysB-D29 was studied. Interestingly, omitting surfactant from the assay buffer resulted in a notable increase in the specific activity of the enzyme against *p*-NPB to 1.61 U/mg from 0.94 U/mg in presence of Triton X-100. In contrast, the specific activity of the enzyme dropped from 4.8 to 1.4 U/mg against *p*-NPL and from 0.55 to 0.07 U/mg against *p*-NPP, respectively, in the absence of Triton X-100. It is however noteworthy that LysB-D29 retains partial activity with long chain substrates even in the absence of the surfactant. Moreover, LysB-D29 was shown to have the same activity pattern (i.e., acting instantly with no need for interfacial activation) on different concentrations of short (*p*-NPB) and long (*p*-NPP) chain *p*-NP substrates (Figure 5).

## 4. Discussion

To date, more than 10,500 mycobacteriophages have been isolated and ≈1800 were fully sequenced [39]. Despite these massive numbers, few reports are available on mycobacteriophage LysB endolysins including LysB-Ms6, -D29, -TM4, -Ardmore, -Bxz2, -BCTU-1, and -L5 [11,13,17,18,36,40,41]. Furthermore, the lack of resolved crystal structure of LysB proteins except LysB-D29, has been the motivation to use the powerful bioinformatics tools to understand the structural diversity/identity and the interaction between the ligands and the active site. Therefore, homology modeling and docking studies were sought, with implementation of proper evaluation tools for quality check, until further experimental evidence is obtained through protein crystallization and kinetics analyses.

Being members of the α/β hydrolase family, LysBs share common features of cutinases, esterases, and lipases [11,13,36]. Multiple sequence alignment and structural alignment of LysB-D29 with different relative α/β hydrolases showed higher degree of identity to esterases and cutinases than lipases (Figures S1–S4). LysB-D29 resembles classical cutinases (*F. solani* and *H. insolens* cutinases) in lacking the lid domain [38], which is present in the atypical cutinase from *T. reesei* (region-1; Figures S1 and S3) [42] and all aligned lipases (regions 1 and 2; Figures S2 and S4) [43,44,45]. On the other hand, all LysB proteins possess a long linker domain that is missing in all esterases and cutinases but seems to align well with lipases (Figures S1 and S2). This linker domain constitutes the acyl binding site in many LysB proteins, and its movement was considered to be important for accepting long chain fatty substrates [13]. Therefore, LysB-D29 can be considered as intermediary between cutinases and lipases, having the advantages of both.

The potential absence of lid domain (like classical cutinases) gives LysB proteins the advantage of being activated by default, avoiding the need for interfacial activation (a common phenomenon of lipases) prior to reaction with fatty molecules [46]. In an earlier study, the increased esterase activity of LysB-Ms6 and -Bxz2 against *p*-NPB in the presence of surfactants (Tween 80 and Triton X-100) was attributed to the lid domain undergoing conformational changes resulting in a more open form of the active site [17]. This assumption contradicts the fact that enzymes having lid domain only show detectable activity on partially soluble substrates (e.g., *p*-NPB) at substrate concentrations exceeding the solubility limit or in the presence of surfactants (oil/water interface) where the active site opens by moving the lid out [47,48]. Both LysB-Ms6 and -Bxz2 showed specific activity exceeding 0.1 and 1.5 U/mg, respectively, at *p*-NPB concentration of 1 mM (below the solubility limit) without the addition of any surfactants [17]. Moreover, we observed a profound increase in the specific activity of LysB-D29 against *p*-NPB in a surfactant-free reaction and also noticed that addition of Triton X-100 increases the auto-degradation of *p*-NPB, which may give false positive results. Similarly, *C. rugosa* lipase A and B could achieve a considerable increase (~100 fold) in their specific activities upon removing Triton X-100 from the reaction buffer (Table S5) [49]. This anomaly was attributed to the aggregation of *p*-NPB molecules in the absence of surfactants, which provokes interfacial activation of these lipases even in the absence of surfactants. However, LysB-D29 retains ~30% and 13% of its specific activity on *p*-NPL and *p*-NPP, respectively, in contrast to *C. rugosa* lipase A and B whose activities on the same substrates were demolished by removing Triton X-100 from the reaction solution (Table S5).

In the same context, lipases known for losing the lid domain, e.g., guinea pig pancreatic lipase showed an esterase-like pattern of activity against small chain partially soluble substrates, e.g., *p*-NPB [44]. On the other hand, *T. reesei* cutinase (atypical cutinase having lid domain) shows interfacial activation behavior and higher specificity for longer chain fatty substrates and, thus, has been reported to have the kinetic and structural features of a ‘true lipase’ [42] (Figures S1 and S3). The reaction of LysB-D29 with different concentrations of short chain (*p*-NPB) and long chain (*p*-NPP) substrates showed activity pattern very identical to that of esterases as they act instantly on their substrates and their activity reaches a plateau at substrate concentrations below the solubility limit [47,50] (Figure 5). Moreover, the activity of LysB enzymes was shown to be better than cutinases on short chain fatty substrates (higher V_max_ on *p*-NPB) and lower than lipases on long chain lipids (lower V_max_ values on *p*-NPP) (Table S5). Therefore, by their default activity on fatty substrates, we propose that LysB proteins lack the typical lid domain, and are not considered as true lipases despite sharing some common features with them.

Lipases exhibit high specificity on long chain fatty substrates due to the large hydrophobic acyl binding site (represented in many lipases by the lid domain) and the deep wide conformations of their active site’s architecture [42,51,52]. Increased activity of *F. solani* cutinase was reported on hydrophobic substrates such as olive oil emulsions by increasing the hydrophobicity of its acyl binding loop [53]. Moreover, *T. reesei* cutinase showed higher lipolytic activity than *F. solani* cutinase on olive oil, due to its long acyl binding site that aligns well to the linker domain of LysB-D29 [42] (Figure S3). These observations emphasize the importance of the acyl binding region for specificity with hydrophobic substrates as most LysB proteins resemble lipases in exhibiting manifold deep conformations of their active sites with long acyl binding sites represented by their linker domain (Figure S8).

Based on the structural alignment and docking results, we could assume that differences in loops 1–5 among LysB proteins may contribute to differences in positions of the catalytic triad residues relative to each other, with the major role played by the hypervariable loop-5. LysB models with loop-5 motif length range of 9–19 residues (14 residues in LysB-D29), are likely to have the “long tunnel, steep funnel or long shallow bowl” conformations of their active sites and hence can accept long chain fatty substrates (Table S3). The tunnels of the LysBs are closely similar to that of *C. rugosa* lipase which was reported to accept an inhibitor molecule matching a C17 fatty acid [54] (Figure S8-IA) and also showing high preference for long chain fatty acids (C16) [55].

According to de Maria and colleagues [56], this tunnel pattern is not common in lipases and exists exceptionally in *C. rugosa* and *Geotrichum candidum* lipases [54], which can be regarded as nonspecific lipases acting on a broad range of fatty acid chain lengths. In general, the tunnel conformation confers many advantages over shallow pockets including high binding affinity and activity in addition to broad spectrum of binding modes since large surface area and more residues are available for contact with substrates [57]. Unlike tunnels of lipases, LysB tunnels have the advantage of being superficial (not deeply buried in the protein core) and are dually opened at their entrance (alcohol moiety of the ligand) and exit (acyl side chain of the ligand), providing higher access to very long fatty substrates since neither the head of the ligand nor its tail is limited by a wall [51,58] (Figure S8-I).

The second conformation observed in seven LysB models was subdivided into very deep (type-1) and less deep (type-2) funnels. Type-1 deep funnel conformation was similar to *C. antarctica* lipase B (CALB) and could accept up to 13-C fatty substrates [54,59], while the second type resembles the conformation of *P. cepacia* lipase [60] which could accommodate up to 14-C fatty substrates [54] (Figure S8-II). The long shallow bowl conformation represented by LysB-Omega is very similar to that of *R. miehei* lipase (Figure S8-IV) previously reported to accept long chain ligands up to C18 [54,61].

In contrast, five LysB models with loop-5 motifs shorter than nine residues (6–7 residues) were found to have the “superficial funnel” conformation (Table S3), which was similar to that of human pancreatic lipase [62] and *F. solani* cutinase [63] active sites accepting up to 8-C fatty substrates [54] (Figure S8-III). LysB-Goose 3D model showed a deeply buried cave conformation similar to that of acetylcholine esterase enzyme [54] where the acyl binding site is short and close to the protein surface accommodating *p*-NP ligand tail only up to 8-C fatty substrates (Figure S8-V).

These docking results were further supported by structural alignment of LysB active sites to their counterparts of the α/β hydrolases and quantitative measurements of the active site volume and solvent accessible surface area of each LysB model (Table S4).

Based on these observations, we suggest that the high diversity of loop-5 length among LysB proteins might have a role in varying the conformation of their active sites, which in turn would influence their activity towards fatty substrates. This suggestion is supported by results of the enzymatic assays on *p*-NPL and *p*-NPP (serving as medium and long acyl side chain substrate, respectively). LysB with a long loop-5 e.g., LysB-D29 having a long tunnel-shaped active site achieved high specific activity with *p*-NPP substrate, followed by LysB-Omega forming long shallow bowl conformation, and LysB-Saal with very deep funnel conformation (Table 3 and Tables S3 and S5). Furthermore, the predicted affinity of LysB enzymes towards *p*-NP ligands (in terms of docking scores of the predicted poses) matches the experimental affinity expressed as K_m_ values (recovered from the kinetic data of the enzymatic assays [64]) (Tables S3 and S5). For example, the docking scores for the achieved poses of long chain *p*-NP ligands (e.g., *p*-NPL and *p*-NPP) with LysB-D29, -Saal, -Obama12 have better values than short chain *p*-NP ligands (e.g., *p*-NPB and *p*-NPC) (Table S3). Similarly, LysB-D29, -Saal and -Obama12 enzymes were reported to have lower K_m_ values (i.e., higher affinity) with long chain *p*-NP substrates (*p*-NPL and *p*-NPP) than short chain ones (*p*-NPB) (Table S5).

Although the four LysB enzymes (LysB-Obama12, -SWU1, -Babyray, and -Palestino) also have tunnel-shaped active sites, they showed higher activities with *p*-NPL than *p*-NPP. This activity pattern could also be explained by docking studies. Upon docking *p*-NPP ligand to the tunnel-shaped active sites of these enzymes, all top ranked poses of the *p*-NPP ligand have inverted conformation in contrast to that with LysB-D29. This inverted conformation provides insufficient hydrophobic surface for the ligand’s acyl side chain, which is faced by the hydrophilic mouth of the tunnel while the hydrophobic exit harbors the head of the ligand where it is very hard to reach the catalytic triad (Figure S8-IB). The absence of sufficient hydrophobic acyl binding region may also account for the sharp drop in activity of LysB-Obama12, -SWU1, -Babyray, and -Palestino with *p*-NPP substrate and also having much lower affinity (higher K_m_ values) for *p*-NPP than LysB-D29 (Table 3 and Table S5). We can similarily explain the results from an earlier report on the higher activity of LysB-Ms6 with tunnel-shaped active site on long-chain substrates in contrast to LysB-Bxz2 with superficial funnel conformation that had higher activity with shorter chain substrates [17] (Figure S8-I-B-4 and 8-III-3, Table S5).

Regarding the previously ambiguous GXP motif, structural alignments revealed absolute conservation of its position between members of the catalytic triad in all LysB models, cutinases, and some lipases (Figures S3, S4, and S7). Docking studies suggest its likely role in stabilization of the active site through forming H-bonds with the catalytic residues (either Asp e.g., LysB-Hades and -Ms6, or His, e.g., relative esterases, cutinases, and lipases), or in providing additional oxyanion hole residues (Figure S9). The exact role of the GNP motif in LysB enzymes can be deciphered by mutagenesis studies.

## 5. Conclusions

The present study suggests that LysB enzymes could be a link between cutinases and lipases through their activity on soluble and aggregated substrates without interfacial activation like cutinases and on longer substrates as lipases. Although only one available 3D structure, the use of in silico analysis techniques including molecular homology modeling, structural alignments, and docking studies revealed differences among LysB structures that could be related to variations in their enzymatic specificities and activities. The structural differences were particularly attributed to length of the hypervariable loop-5 that appears to play a role in constitution of LysB active sites and that could be used as a parameter in classification of LysBs. Resolving more 3D structures of the LysB enzymes and preparing mutants with different loop-5 length and composition would be needed to obtain more accurate structures and decipher the precise role of the loop in LysB enzymes. It would also be interesting to determine if the differences in the active site conformation are reflected in the activity of the enzymes against the mycolic acid layer of the mycobacteria and their potential as anti-mycobacterials.

## Figures and Tables

**Table 1:** Relative members of α/β hydrolase family used in multiple sequence alignment and structural alignment.

Name	Abb.	Pdb ID	UniProt ID	Z-Score	RMSD	Aligned Residues	Total Protein Length ^1^	(%) Identity to LysB-D29
*Penicillium purpureogenum* Acetylxylan esterase	PPA	1G66	O59893	18.9	2.2	162	234	22
*Fusarium solani* cutinase	FSC	1XZM	P00590	16.4	2.6	156	230	20
*Humicola insolens* cutinase	HIC	4OYL	A0A075B5G4	16.2	2.6	155	194	21
*Trichoderma reesei* cutinase	TRC	4PSE	G0RH85	14.2	2.6	148	254	22
Human pancreatic lipase	HuPL	1LPB	P16233	8.6	2.8	147	465	15
*Pseudomonas cepacia* lipase	PCL	1YS1	P22088	9.1	3.1	143	364	16
*Candida rugosa* lipase	CRL	1Lpo	P20261	7.3	3.4	100	549	12

^1^ Length according to UniprotKB. Note: Crystal structures were retrieved from Dali server.

**Table 2:** Classification of LysB homologs according to their percentage identity to LysB-D29 and chain lengths.

Group	Subgroups	(%) Identity to LysB-D29	Chain Length	Comment
Group-1	—	100–89	254	Same length
Group-2	2A	77–74.5	~325	Extra 68 residues in N-terminal ^1^
	2B	76.6–75.6		Extra 75 residues in N-terminal ^1^
	2C	70–68		Extra 78 residues in N-terminal ^1^ (Except LysB-Sheen is short 246 residues)
Group-3		63	244–246	Shorter
Group-4		64–58	321	Extra 79 residues N-terminal ^1^
Group-5	5A	48.8–47.3	342–343	Extra 97 residues N-terminal ^2^
	5B	47	321	Extra 82 residues N-terminal ^2^
Group-6	6A	43-41.1	332–333	Extra 89 residues N-terminal ^2^
	6B	42.3–41.0	285	Extra 15 residues N-terminal and 14 residues C-terminal ^1^
Group-7	7A	40–37	322–346	Extra 72-95 residues N-terminal ^2^
	7B	36–35	326	Extra 16–71 residues N-terminal and 7-22 residues C-terminal ^1^
	7C	32–30	252–290	Shorter-Extra 16 residues N-terminal and 7 residues C-terminal ^1^

^1^ extra residues forming no motif. ^2^ extra residues forming peptidoglycan binding domains or other catalytic domains.

**Table 3:** Activities of LysB enzymes with *p*-nitrophenyl laurate (*p*-NPL) and *p*-nitrophenyl palmitate (*p*-NPP) substrates.

LysB-	Specific Activity (U/mg)		Relative Activity to LysB-D29	
	*p*-NPL	*p*-NPP	*p*-NPL	*p*-NPP
**D29**	4.8 ± 0.09	0.55 ± 0.07	100.0%	100.0%
**Omega**	9.7 ± 0.3	0.32 ± 0.02	201.0%	59.8%
**Saal**	2.1 ± 0.3	0.25 ± 0.02	43.6%	45.7%
**Obama12**	0.9 ± 0.03	0.16 ± 0.01	19.1%	28.2%
**SWU1**	0.09 ± 0.01	0.007 ± 0.0009	1.9%	1.3%
**BabyRay**	0.02 ± 0.001	0.002 ± 0.003	0.4%	0.4%
**Palestino**	0.01 ± 0.001	0	0.2%	0.0%

## Supplementary Materials

The following are available online at https://www.mdpi.com/2218-273X/10/1/45/s1, Figure S1: Multiple sequence alignment of LysB-D29 with relative members of the α/β hydrolase family including *Penicillium purpureogenum* Acetylxylan esterase (PPA), *Fusarium solani* cutinase (FSC), *Humicola insolens* cutinase (HIC), and *Trichoderma reesei* cutinase (TRC). Identical residues are labeled by *asterisk*, conserved residues labeled by *colon* and *full stop*, indicating semi-conserved residues. Residues forming the active site are shaded in red color. Region-1 (lid domain) in TRC is highlighted in yellow, Region-2 is highlighted in gray, Region-3 (linker domain in LysB-D29) and acyl binding site in PPA esterase and cutinases are highlighted in green. Oxyanion hole and GXP residues are highlighted in pink, Figure S2: Multiple sequence alignment of D29 LysB with relative members of the α/β hydrolase family including *Candida rugosa* lipase (CRL), *Pseudomonas cepacia* lipase (PCL), and human pancreatic lipase (HuPL). Identical residues are labeled by *Asterisk*, conserved residues labeled by *colon* and *full stop* indicate semi-conserved residues. Residues forming the active site are shaded in red. Oxyanion hole and GXP residues are highlighted in pink. Region-1 in CRL and Region-2 in PCL and HuPL (lid domains) are highlighted in yellow. Region-3 (linker domain) in LysB-D29 and other lipases are highlighted in green, Figure S3: (a) Total alignment of three-dimensional structural alignment of 3D structures of LysB-D29 (red color) and its relative members of the α/β hydrolase family including: *Fusarium solani* cutinase (blue), *Humicola insolens* cutinase (purple), *Trichoderma reesei* cutinase (orange), *Penicillium purpureogenum* Acetylxylan esterase (cyan) showing the lid domain of *T. reesei* cutinase (orange), linker domain of LysB-D29 (red). (b) Focus view: showing catalytic triad residues, oxyanion hole residues and GXP residues (each is shown in its color), co-crystalized inhibitor molecule (black), and the rest of protein (gray), Figure S4: Total alignment of three-dimensional structural alignment of 3D structures of LysB-D29 and its relative members of the α/β hydrolase family including: (a) *Pseudomonas cepacia* lipase, (c) *Candida rugosa* lipase, and (e) human pancreatic lipase, showing lid domain (yellow), linker domain of LysB-D29 (red), and acyl binding site in lipases (green =). Focus views of (b) alignment of LysB-D29 to *P*. *cepacia*, (d) alignment of LysB-D29 to *C. rugosa*, and (f) alignment of LysB-D29 to human pancreatic lipase, showing triad residues, oxyanion hole residues, and GXP residues in red (LysB-D29) and green (aligned lipases) and co-crystalized inhibitor molecule (black), Figure S5: Multiple sequence alignment of D29 LysB with its homologous LysB proteins. Identical residues are labeled by *Asterisk*, conserved residues labeled by *colon* and *full stop* indicates semi-conserved residues. Residues forming the active site are shaded in red. Extra His residue is highlighted in yellow. Oxyanion hole and GXP residues are shaded in pink. Residues forming β-strands and α-helices are highlighted in gray. Region-1 constitutes extra N-terminal residues. Region-2 constitutes residues forming hypervariable loop-5. Loops, β-strands and α-helices forming the linker domain are written in red, Figure S6: Ramachandran plots of the generated 30 LysB homology models, Figure S7: Three-dimensional structural alignment of 3D structures of LysB-D29 to members of the seven groups representing homologous LysB models, (a) Gp-1 models, (b) Gp-2A models, (c) Gp-2B models, (d) Gp-2C models, (e) Gp-3 models, (f) Gp-4 models, (g) Gp-5A and B models, (h) Gp-6A models, (i) Gp-6B models, (j) Gp-7A models, (k) Gp-7B model, and (l) Gp-7C models. The catalytic Ser and Asp residues (yellow), the catalytic His residue (each in its model’s color), oxyanion hole and GNP residues (pink) and differences in loops 1–5 are highlighted in each of the model’s color accordingly, Figure S8: 3D conformations of poses of *p*NP ligands upon docking to LysB proteins and other lipases and cutinases. Each diagram illustrates (in order) the overall surface of protein with its ligand, the shape of active site with the docked ligand, the interactions of ligand atoms with different residues of its protein active site. Hydrophilic residues (pink), hydrophobic residues (green), *p*-NP ligands (black). Orientation pose: ligand-protein conformation where the catalytic Ser faces ester ligand’s ester bond (C=O) with no H-bond formation. NDP (no detected pose), neither binding nor orientation pose, were detected. Stars indicate catalytic triad residues of LysB-D29, Figure S9: Three-dimensional conformations of active sites of (a) *F. solani* cutinase, (b) *P. cepacia* lipase, (c) *R. miehei* lipase, (d) *P. purpureogenum* Acetylxylan esterase (e), *T. reesei* cutinase (f), LysB-Hades, and (g) LysB-Ms6 showing the H-bonds between the glycine residue of the GNP motif with members of the catalytic triad: Histidine residues (a–e) and aspartate residues (f–g) (illustrated by black arrows). Co-crystalized inhibitors and *p*-NP ligands (black), Table S1: Evaluation parameters of the generated LysB homology models, Table S2: LysB homologous proteins representatives of the seven groups, chain length, and domain diversity, Table S3: Types of active site conformations of LysB models and the best docking score upon docking of *p*-NP ligands, Table S4: Quantitative measurements and structural alignments of LysB active sites to their counterparts of the α/β hydrolases, Table S5: Specific activities and kinetic properties of LysB enzymes in comparison to relative cutinases and lipases.

## References

[1] Kealey A., Smith R. (2010). Neglected tropical diseases: Infection, modeling, and control. J. Health Care Poor Underserved.

[2] Leung A.N. (1999). Pulmonary tuberculosis: The essentials. Radiology.

[3] WHO (2018). Global Tuberculosis Report 2018.

[4] WHO (2018). Global Antimicrobial Resistance Surveillance System (GLASS) Report: Early Implementation 2017–2018.

[5] Falagas M.E., Karageorgopoulos D.E. (2008). Pandrug resistance (PDR), extensive drug resistance (XDR), and multidrug resistance (MDR) among Gram-negative bacilli: Need for international harmonization in terminology. Clin. Infect. Dis..

[6] Pai M., Memish Z.A. (2016). Antimicrobial resistance and the growing threat of drug-resistant tuberculosis. J. Epidemiol. Glob. Health.

[7] Sani M., Houben E.N.G., Geurtsen J., Pierson J., de Punder K., van Zon M., Wever B., Piersma S.R., Jimenez C.R., Daffe M. (2010). Direct visualization by cryo-EM of the mycobacterial capsular layer: A labile structure containing ESX-1-secreted proteins. PLoS Pathog..

[8] Daffe M., Draper P. (1998). The envelope layers of mycobacteria with reference to their pathogenicity. Adv. Microb. Physiol..

[9] Bhamidi S., Scherman M.S., Rithner C.D., Prenni J.E., Chatterjee D., Khoo K.H., McNeil M.R. (2008). The identification and location of succinyl residues and the characterization of the interior arabinan region allow for a model of the complete primary structure of *Mycobacterium tuberculosis* mycolyl arabinogalactan. J. Biol. Chem..

[10] Daffe M., Reyrat J.-M. (2008). The Mycobacterial Cell Envelope.

[11] Gil F., Catalao M.J., Moniz-Pereira J., Leandro P., McNeil M., Pimentel M. (2008). The lytic cassette of mycobacteriophage Ms6 encodes an enzyme with lipolytic activity. Microbiology.

[12] Gil F., Grzegorzewicz A.E., Catalao M.J., Vital J., McNeil M.R., Pimentel M. (2010). Mycobacteriophage Ms6 LysB specifically targets the outer membrane of *Mycobacterium smegmatis*. Microbiology.

[13] Payne K., Sun Q.A., Sacchettini J., Hatfull G.F. (2009). Mycobacteriophage Lysin B is a novel mycolylarabinogalactan esterase. Mol. Microbiol..

[14] Payne K.M., Hatfull G.F. (2012). Mycobacteriophage Endolysins: Diverse and Modular Enzymes with Multiple Catalytic Activities. PLoS ONE.

[15] Catalao M.J., Pimentel M. (2018). Mycobacteriophage Lysis enzymes: Targeting the mycobacterial cell envelope. Viruses.

[16] Hatfull G.F., Jacobs-Sera D., Lawrence J.G., Pope W.H., Russell D.A., Ko C.C., Weber R.J., Patel M.C., Germane K.L., Edgar R.H. (2010). Comparative genomic analysis of 60 Mycobacteriophage genomes: Genome clustering, gene acquisition, and gene size. J. Mol. Biol..

[17] Grover N., Paskaleva E.E., Mehta K.K., Dordick J.S., Kane R.S. (2014). Growth inhibition of *Mycobacterium smegmatis* by mycobacteriophage-derived enzymes. Enzyme Microb. Technol..

[18] Lai M.J., Liu C.C., Jiang S.J., Soo P.C., Tu M.H., Lee J.J., Chen Y.H., Chang K.C. (2015). Antimycobacterial activities of endolysins derived from a Mycobacteriophage, BTCU-1. Molecules.

[19] Altschul S.F., Madden T.L., Schaffer A.A., Zhang J.H., Zhang Z., Miller W., Lipman D.J. (1997). Gapped BLAST and PSI-BLAST: A new generation of protein database search programs. Nucleic Acids Res..

[20] Motif Search. http://www.genome.jp/tools/motif/.

[21] Sievers F., Higgins D.G. (2018). Clustal Omega for making accurate alignments of many protein sequences. Protein Sci..

[22] Tamura K., Stecher G., Peterson D., Filipski A., Kumar S. (2013). MEGA6: Molecular evolutionary genetics analysis version 6.0. Mol. Biol. Evol..

[23] Krieger E., Vriend G. (2014). YASARA view—Molecular graphics for all devices–From smartphones to workstations. Bioinformatics.

[24] Nurisso A., Daina A., Walker R.C., Orry A.J., Abagyan R. (2012). A practical introduction to molecular dynamics simulations: Applications to homology modeling. Homology Modeling.

[25] Bernstein F.C., Koetzle T.F., Williams G.J., Meyer E.F., Brice M.D., Rodgers J.R., Kennard O., Shimanouchi T., Tasumi M. (1977). The Protein Data Bank. A computer-based archival file for macromolecular structures. Eur. J. Biochem..

[26] Lüthy R., Bowie J.U., Eisenberg D. (1992). Assessment of protein models with three-dimensional profiles. Nature.

[27] Laskowski R.A., Macarthur M.W., Moss D.S., Thornton J.M. (1993). Procheck—A program to check the stereochemical quality of protein structures. J. Appl. Crystallogr..

[28] Colovos C., Yeates T.O. (1993). Verification of protein structures—Patterns of nonbonded atomic interactions. Protein Sci..

[29] Pontius J., Richelle J., Wodak S.J. (1996). Deviations from standard atomic volumes as a quality measure for protein crystal structures. J. Mol. Biol..

[30] Wiederstein M., Sippl M.J. (2007). ProSA-web: Interactive web service for the recognition of errors in three-dimensional structures of proteins. Nucleic Acids Res..

[31] Holm L., Laakso L.M. (2016). Dali server update. Nucleic Acids Res..

[32] Pettersen E.F., Goddard T.D., Huang C.C., Couch G.S., Greenblatt D.M., Meng E.C., Ferrin T.E. (2004). UCSF chimera—A visualization system for exploratory research and analysis. J. Comput. Chem..

[33] (2014). Molecular Operating Environment (MOE 2014.0901).

[34] Tobi D., Bahar I. (2005). Structural changes involved in protein binding correlate with intrinsic motions of proteins in the unbound state. Proc. Natl. Acad. Sci. USA.

[35] Sotriffer C.A. (2011). Accounting for induced-fit effects in docking: What is possible and what is not?. Curr. Top. Med. Chem..

[36] Henry M., Coffey A., O’Mahony J., Sleator R.D. (2011). Comparative modelling of LysB from the mycobacterial bacteriophage Ardmore. Bioeng. Bugs.

[37] Ginalski K. (2006). Comparative modeling for protein structure prediction. Curr. Opin. Struct. Biol..

[38] Rauwerdink A., Kazlauskas R.J. (2015). How the same core catalytic machinery catalyzes 17 different reactions: The serine-histidine-aspartate catalytic triad of alpha/beta-hydrolase fold enzymes. ACS Catal..

[39] PhagesDB. https://phagesdb.org.

[40] Hassan S., Dusthackeer A., Subramanyam B., Ponnuraja C., Sivaramakrishnan G.N., Kumar V. (2010). Lytic efficiency of mycobacteriophages. Open Syst. Biol. J..

[41] Miller S. (2015). Composition for Use in Mycobacteria Therapy. U.S. Patent.

[42] Roussel A., Amara S., Nyyssola A., Mateos-Diaz E., Blangy S., Kontkanen H., Westerholm-Parvinen A., Carriere F., Cambillau C. (2014). A cutinase from *Trichoderma reesei* with a lid-covered active site and kinetic properties of true lipases. J. Mol. Biol..

[43] Grochulski P., Li Y.G., Schrag J.D., Bouthillier F., Smith P., Harrison D., Rubin B., Cygler M. (1993). Insights into interfacial activation from an open structure of *Candida rugosa* lipase. J. Biol. Chem..

[44] Hjorth A., Carriere F., Cudrey C., Woldike H., Boel E., Lawson D.M., Ferrato F., Cambillau C., Dodson G.G., Thim L. (1993). A structural domain (the Lid) found in pancreatic lipases is absent in the guinea-pig (phospho) lipase. Biochemistry.

[45] Kim K.K., Song H.K., Shin D.H., Hwang K.Y., Suh S.W. (1997). The crystal structure of a triacylglycerol lipase from *Pseudomonas cepacia* reveals a highly open conformation in the absence of a bound inhibitor. Structure.

[46] Verger R. (1997). ‘Interfacial activation’of lipases: Facts and artifacts. Trends Biotechnol..

[47] Ferrato F., Carriere F., Sarda L., Verger R. (1997). A critical reevaluation of the phenomenon of interfacial activation. Methods Enzymol..

[48] Maruyama T., Nakajima M., Ichikawa S., Nabetani H., Furusaki S., Seki M. (2000). Oil-water interfacial activation of lipase for interesterification of triglyceride and fatty acid. J. Am. Oil Chem. Soc..

[49] Redondo O., Herrero A., Bello J.F., Roig M.G., Calvo M.V., Plou F.J., Burguillo F.J. (1995). Comparative kinetic study of lipases A and B from *Candida rugosa* in the hydrolysis of lipid *p*-nitrophenyl esters in mixed micelles with Triton X-100. Biochim. Biophys. Acta.

[50] Martinelle M., Holmquist M., Hult K. (1995). On the interfacial activation of *Candida antarctica* lipase-A and lipase-B as compared with *Humicola lanuginosa* lipase. Biochim. Biophys. Acta.

[51] Dominguez de Maria P., Sanchez-Montero J.M., Sinisterra J.V., Alcantara A.R. (2006). Understanding *Candida rugosa* lipases: An overview. Biotechnol. Adv..

[52] Panizza P., Cesarini S., Diaz P., Rodriguez Giordano S. (2015). Saturation mutagenesis in selected amino acids to shift *Pseudomonas* sp. acidic lipase Lip I.3 substrate specificity and activity. Chem. Commun..

[53] Egmond M.R., de Vlieg J. (2000). Fusarium Solani Pisi Cutinase. Biochimie.

[54] Pleiss J., Fischer M., Schmid R.D. (1998). Anatomy of lipase binding sites: The scissile fatty acid binding site. Chem. Phys. Lipids.

[55] Lee L.C., Chen Y.T., Yen C.C., Chiang T.C., Tang S.J., Lee G.C., Shaw J.F. (2007). Altering the substrate specificity of *Candida rugosa* LIP4 by engineering the substrate-binding sites. J. Agric. Food Chem..

[56] de Maria P.D., Carboni-Oerlemans C., Tuin B., Bargeman G., van der Meer A., van Gemert R. (2005). Biotechnological applications of *Candida antarctica* lipase A: State-of-the-art. J. Mol. Catal. B.

[57] Marques S.M., Daniel L., Buryska T., Prokop Z., Brezovsky J., Damborsky J. (2017). Enzyme tunnels and gates as relevant targets in drug design. Med. Res. Rev..

[58] Cygler M., Schrag J.D. (1999). Structure and conformational flexibility of *Candida rugosa* lipase. Biochim. Biophys. Acta.

[59] Stauch B., Fisher S.J., Cianci M. (2015). Open and closed states of *Candida antarctica* lipase B: Protonation and the mechanism of interfacial activation. J. Lipid Res..

[60] Yang J., Koga Y., Nakano H., Yamane T. (2002). Modifying the chain-length selectivity of the lipase from *Burkholderia cepacia* KWI-56 through in vitro combinatorial mutagenesis in the substrate-binding site. Protein Eng..

[61] Derewenda Z.S., Derewenda U., Dodson G.G. (1992). The crystal and molecular-structure of the *Rhizomucor miehei* Triacylglyceride lipase at 1.9-Angstrom resolution. J. Mol. Biol..

[62] Dugi K.A., Dichek H.L., Santamarinafojo S. (1995). Human hepatic and lipoprotein-lipase—The loop covering the catalytic site mediates lipase substrate-specificity. J. Biol. Chem..

[63] Longhi S., Mannesse M., Verheij H.M., DeHaas G.H., Egmond M., KnoopsMouthuy E., Cambillau C. (1997). Crystal structure of cutinase covalently inhibited by a triglyceride analogue. Protein Sci..

[64] Abouhmad A. (2019). Phage–Derived Endolysins as Potential Antibacterials: A Study of Peptidoglycan Hydrolase and Mycolylarabinogalactan Esterase Enzymes. Ph.D. Thesis.

