# Supplementary material for: Comparative Structural Analysis of Different Mycobacteriophage-Derived Mycolylarabinogalactan Esterases (Lysin B)
Source: Biomolecules. 2019 Dec 27;10(1):45. doi: 10.3390/biom10010045 (PMC7022511; doi:10.3390/biom10010045)
Supplement: Supplementary file 1 [file biomolecules-10-00045-s001.zip › Supplementary/Supp. fig. 1.pdf]

```
                                        Region-1(lid domain)          Region-2(N-terminal α-helices)

LysB-D29   1 --------------------------------------------------------------------------------------------------MSKPWLFTVHGTGQ
PPA        1 MHSKFFAASLLGLGAAA------------------------------------------------IPLEGV--MEKRSCPAIHVFGARETTASPGY----------------
FSC        1 MK-FFALTTLLAATASALPTSNPAQ-------------------------------ELEARQLGRTTRDDLINGNSASCRDVIFIYARGSTETGNLGT-LGPSIASNLESAF
HIC        1 -------------------------------------------------------------QLG-AIENGLESGSANACPDAILIFARGSTEPGNMGITVGPALANGLESHI
TRC        1 MRSLAILTTLLAGHAFAYPKPAPQSVNRRDWPSINEFLSELAKVMPIGDTITAACDLISDGEDAAASLFGISETENDPCGDVTVLFARGTCDPGNVGVLVGPWFFDSLQTAL

                                                                                     GXSXG

LysB-D29  15 --PDPLGPGLPADTARDVLDIYRWQPIGN-YPAAAFPMWPSVEKGVAELILQIELKLDADPYADFAMAGYSQGAIVVGQVLKHHILPPTGRL-------HRFLHRLKKVIFW
PPA       47 GSSSTVVNGVLSAYPGSTAEAINYPACGGQSSCGGASYSSSVAQGIAAVASAVNSFNSQCPSTKIVLVGYSQGGEIMDVALCGGGDPNQGYTNTAVQLSSSAVNMVKAAIFM
FSC       80 GKDGVWIQGVGGAYRATLGDN--------------ALPRGTSSAAIREMLGLFQQANTKCPDATLIAGGYSQGAALAAASIE--------------DLDSAIRDKIAGTVLF
HIC       51 --RNIWIQGVGGPYDAALATN--------------FLPRGTSQANIDEGKRLFALANQKCPNTPVVAGGYSQGAALIAAAVS--------------ELSGAVKEQVKGVALF
TRC      113 GSRTLGVKGVP--YPASVQD---------------FL-SGSVQNGI-NMANQIKSVLQSCPNTKLVLGGYSQGSMVVHNAAS--------------NLDAATMSKISAVVLF
                     *:                              :    :      .       * : .   *****. :                             :  . :

             GXP                                              Region-3(linker domain and acyl binding site)

LysB-D29 117 GNPMRQKGFAHSDEWIHPVAAPDTLGILEDRLENLEQY-GFEVRDYAHDGDMYASIKED-------DLHEYEVAIGRIVMKASGFIGGRDSVVAQLIELGQRPITEGIALAG
PPA      159 GDPMFRAGLSYEVGTCA-------AGGFDQRPAGFSCPSAAKIKSYCDASDPYCCNGSN-AATHQGYGSEYGSQALA-----------------------------------
FSC      164 GYTKNLQ-----------------------NRGRIPNYPADRTKVFCNTGDLVCTGSLIVAAPHLAYGPDARGPAPE-----------------------------------
HIC      133 GYTQNLQ-----------------------NRGGIPNYPRERTKVFCNVGDAVCTGTLIITPAHLSYTIEARGEAAR-----------------------------------
TRC      192 GDPYY--------------------------GKPVANFDAAKTLVVCHDGDNICQGGDIILLPHLTYAEDADTAAA------------------------------------
             *                                 .      .    .. .*  .               :

                     Region-3

LysB-D29 221 AIIDALTFFARSRMGDKWPHLYNRYPAVEFLRQI
PPA      228 --------FVKSKLG-------------------
FSC      218 --------FLIEKVRAVRGSA-------------
HIC      187 --------FLRDRIRA------------------
TRC      242 --------FVVPLVSHHHHHH-------------
                     *    :
```

**Figure S1**: Multiple sequence alignment of LysB-D29 with relative members of the α/β hydrolase family including *Penicillium purpureogenum* Acetylxylan esterase (**PPA**), *Fusarium solani* cutinase (**FSC**), *Humicola insolens* cutinase (**HIC**) and *Trichoderma reesei* cutinase (**TRC**). Identical residues are labeled by *asterisk*, conserved residues labeled by *colon* and *full stop* indicating semi-conserved residues. Residues forming the active site are shaded in red color. Region-1 (lid domain) in **TRC** is highlighted in yellow, Region-2 is highlighted in gray, Region-3 (linker domain in LysB-D29) and acyl binding site in **PPA** esterase and all cutinases are highlighted in green. Oxyanion hole and GXP residues are highlighted in pink.
