# Supplementary material for: Comparative Structural Analysis of Different Mycobacteriophage-Derived Mycolylarabinogalactan Esterases (Lysin B)
Source: Biomolecules. 2019 Dec 27;10(1):45. doi: 10.3390/biom10010045 (PMC7022511; doi:10.3390/biom10010045)
Supplement: Supplementary file 1 [file biomolecules-10-00045-s001.zip › Supplementary/Supp. fig. 2.pdf]

```
                                                                              Region-1 (lid domain)
                                                                        ________________________________
LysB-D29    1 MSKPWLFTV-H-------------------------------------------------------------------------------GT----GQPDPLGP-GLPAD
CRL         1 MELALALSLIASVAAAPTATLANGDTITGLNAIINEAFLGIPFAEPPVGNLRFKDPVPYSGSLDGQKFTSYGPSCMQQNPEGTYEENLPKAALDLVMQSKVFEA---VSP
PCL         1 MARTMRSRVVA-------------------------------------------------------------------------------GAVACAMSIAPFAGTTAVMT
HuPL        1 MLPLWTLSLLLGAVAGKEVCY------------------------ERLG--CFSDDSPWSGI------------------------------------------------
              *       :

LysB-D29   26 TARDVLDIYRWQPIGNYPAA---------AF---------------------------------PMWPS-VEKGVAE---------------------------------
CRL       108 SSEDCLTINVVRPPGTKAGA---------NLPV----MLWIFGGGFEVGGTSTF------PPAQ----M-ITKSIAMGKPIIHVSVNYRVSSWGFLAGDEIKAEGSANAG
PCL        32 LATTHAAMAATAPAAGYAAT---------RYPI----ILVHG-----LSGTDKY------AGVLEYWYG-IQEDLQQNGA-----TVYVANLSGFQSDDGPNGRGE----
HuPL       36 -TERPLHILPWSPKDVNTRFLLYTNENPNNFQEVAADSSSISGSNFKTNRKTRFIIHGFIDKGEENWLANVCKNLFKVE-----SVNCICVDWKGGSRTGYTQ-ASQNIR
               :     :    *                                                         : :.:
                                           GXSXG                                GXP
                                           _____                                ___
LysB-D29   60 ------LILQIELKLDA--DPYADFAMAGYSQGAIVVGQVLKHHILPPTGRLHRFLHRL-KKVIFWGNPMRQ-------------------------KGFAH---SDEWI
CRL       194 LKDQRLGMQWVADNIAAFGGDPTKVTIFGESAGSMSVMCHIL----W----------ND-GDNTYKGKPLFR-------------------------AGIMQ---SGAMV
PCL       108 ---Q--LLAYVKTVLAA--TGATKVNLVGHSQGGLSSRYVAA--VAP---------DLV-ASVTTIGTPHRG-------------------------SEFAD------FV
HuPL      139 IVG-AEVAYFVEFLQSAFGYSPSNVHVIGHSLGAHAAGEAGR----RTNGTIGRITGLDPAEPCFQGTPELVRLDPSDAKFVDVIHTDGAPIVPNLGFGMSQVVGHLDFF
                        :     *     :.. : * * *.                           .    *.*                              : .       .
                                Region-2 (lid domain)                                                  Region-3
                         ____________________________________                                   _______________________________
LysB-D29  133 HPVAAPD--------------TLGILEDRLE-NLEQY-----------------------------G---------FEV-RDYAHDGDMYASIKEDDLHEYE----VAIG
CRL       261 PSDAV---DGIYGN------EIFDLLASNAGCGSASDKLACLRGVSSDTLEDATNNTPGFLAYSSLRLS------YLPRPDGVNITDDMYALVREGKYANIP----VIIG
PCL       168 QDVLAYDPTGLSSSVIAAFVNVFGILTSSSH-NTNQDALAALQTLTTARAATYNQN------YPSAG---------LGA-PGSCQTGAPTETVGGNTHLLYS----WAGT
HuPL      244 PNGGV-EMPGCKKNILSQIVDIDGIWEGT------RDFAACNHLRSYKYYTDSIVNPDGFAGFPCASYNVFTANKCFPCPSGGCPQMGHYADRYPGKTNDVGQKFYLDTG
                  .                  .:  .                                                :    .             .

                                                       Region-3 (linker domain and acyl binding site)
                                  ____________________________________________________________________________________________
LysB-D29  185 -------------------R----IVMKASGFIGG---RDSV-----VAQLIE-----------------------LGQRPITEGIALAGAIID-ALTFFA---------
CRL       352 D-----------------QN----DEGTFFGTSSLNVTTDAQAREYFKQSFVHAS--------DAEIDT--L--MTAYPGDITQGSPFDTGILNALTPQFKRISAVLGDL
PCL       257 A-----------------IQ----PTLSVFGVTGA---TDTS-----TLPLVDPA---N----VLDLSTLAL--FGTGTVMINRGSGQNDGLVSKCSALYG---------
HuPL      347 DASNFARWRYKVSVTLSGKKVTGHILVSLFGNKGN-----SKQYEIFKGTLKPDSTHSNEFDSDVDVGDLQMVKFIWYNN-----------VINPTLPRVGAS-------
                                 .       .  *  .      :         :                                        ::.

                           Region-3
               ___________________________________
LysB-D29  231 -----------RSRMGDKWPHLYNRYPAVEFL-----------RQ------------------------------------------------------I----------
CRL       429 GFTLARRYFLNHYTGGTKYSFLSKQLSGLPVLGTFHSNDIVFQDYLLGSGSLIYNNAFIAFATDLDPNTAGLLVKWPEYTSSSQSGNNLMMINALGLYTGKDNFRTAGYD
PCL       320 ----------KVLSTSYKWNHLDEINQLLGVRGAYAEDPVAVIRT------------------------------------------------------HANRLKLAGV-
HuPL      434 -------KIIVETNVGKQFNFCSPETVREEVLLTL---------------------------------------------------------------------------
                             . :: .         .
LysB-D29      -----------
CRL       538 ALFSNPPSFFV
PCL           -----------
HuPL      463 -----TPC---
```

Figure S2: Multiple sequence alignment of LysB-D29 with relative members of the α/β hydrolase family including *Candida rugosa* lipase (**CRL**), *Pseudomonas cepacia* lipase (**PCL**) and Human pancreatic lipase (**HuPL**). Identical residues are labeled by *asterisk*, conserved residues labeled by *colon* and *full stop* indicating semi-conserved residues. Residues forming the active site are shaded in red color. Oxyanion hole and GXP residues are highlighted in pink. Region-1 in **CRL** and Region-2 in **PCL** and **HuPL** (lid domains) are highlighted in yellow. Region-3 (linker domain) in LysB-D29 and other lipases are highlighted in green.
