# Supplementary material for: Comparative Structural Analysis of Different Mycobacteriophage-Derived Mycolylarabinogalactan Esterases (Lysin B)
Source: Biomolecules. 2019 Dec 27;10(1):45. doi: 10.3390/biom10010045 (PMC7022511; doi:10.3390/biom10010045)
Supplement: Supplementary file 1 [file biomolecules-10-00045-s001.zip › Supplementary/Supp. fig. 3.pdf]

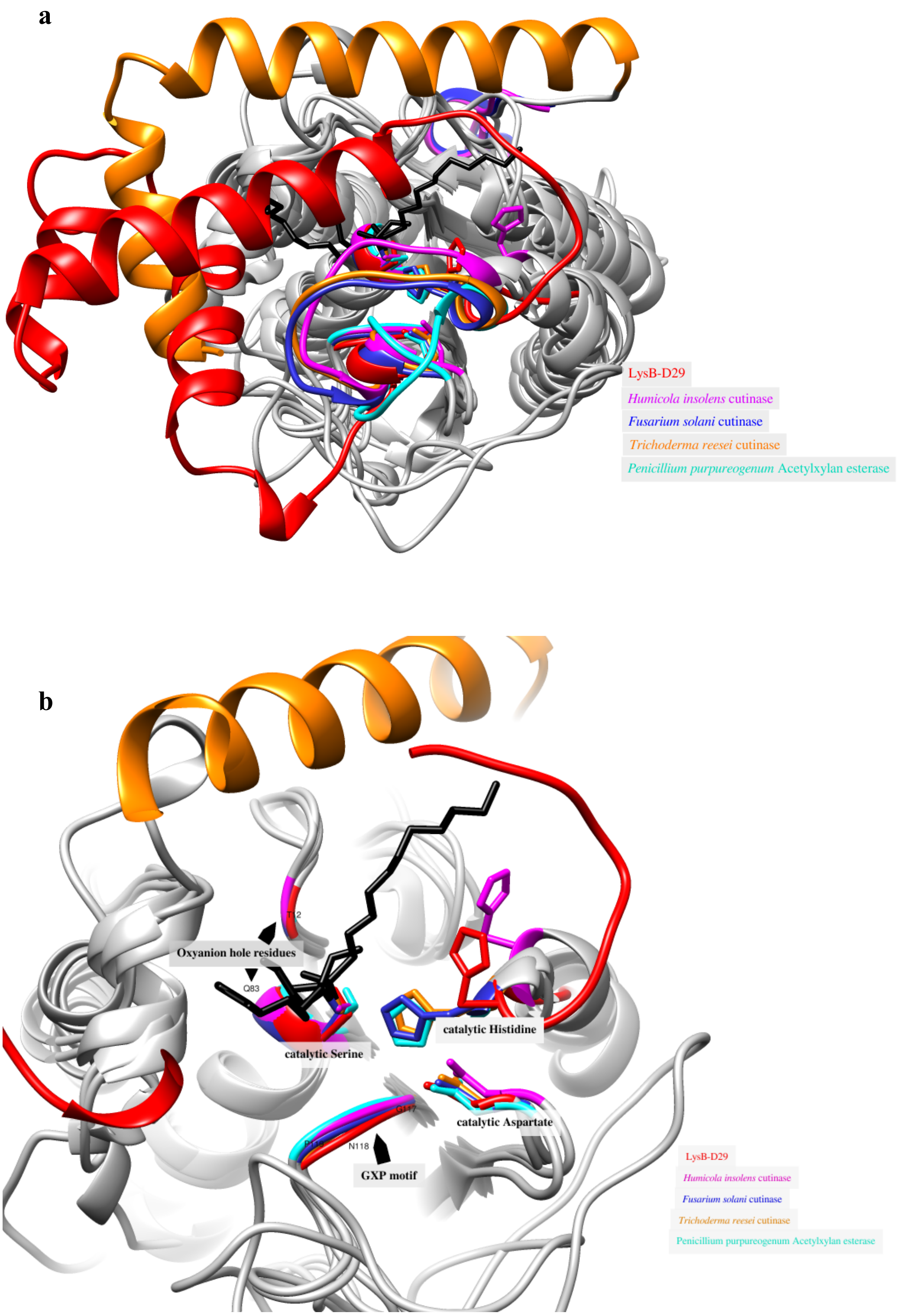

Figure S3. **(a) Total alignment** of three-dimensional structural alignment of 3D structures of LysB-D29 (red color) and its relative members of the α/β hydrolase family including: *Fusarium solani* cutinase (blue color), *Humicola insolens* cutinase (purple color), *Trichoderma reesei* cutinase (orange color), *Penicillium purpureogenum* Acetylxylan esterase (cyan color) showing the lid domain of *T. reesei* cutinase (Orange color), linker domain of LysB-D29 (red color). **(b) Focus view**: showing catalytic triad residues, oxyanion hole residues and GXP residues (each is shown in its color), co-crystalized inhibitor molecule (black), and the rest of protein (gray).
