# Supplementary material for: Comparative Structural Analysis of Different Mycobacteriophage-Derived Mycolylarabinogalactan Esterases (Lysin B)
Source: Biomolecules. 2019 Dec 27;10(1):45. doi: 10.3390/biom10010045 (PMC7022511; doi:10.3390/biom10010045)
Supplement: Supplementary file 1 [file biomolecules-10-00045-s001.zip › Supplementary/Supp. fig. 4.pdf]

a

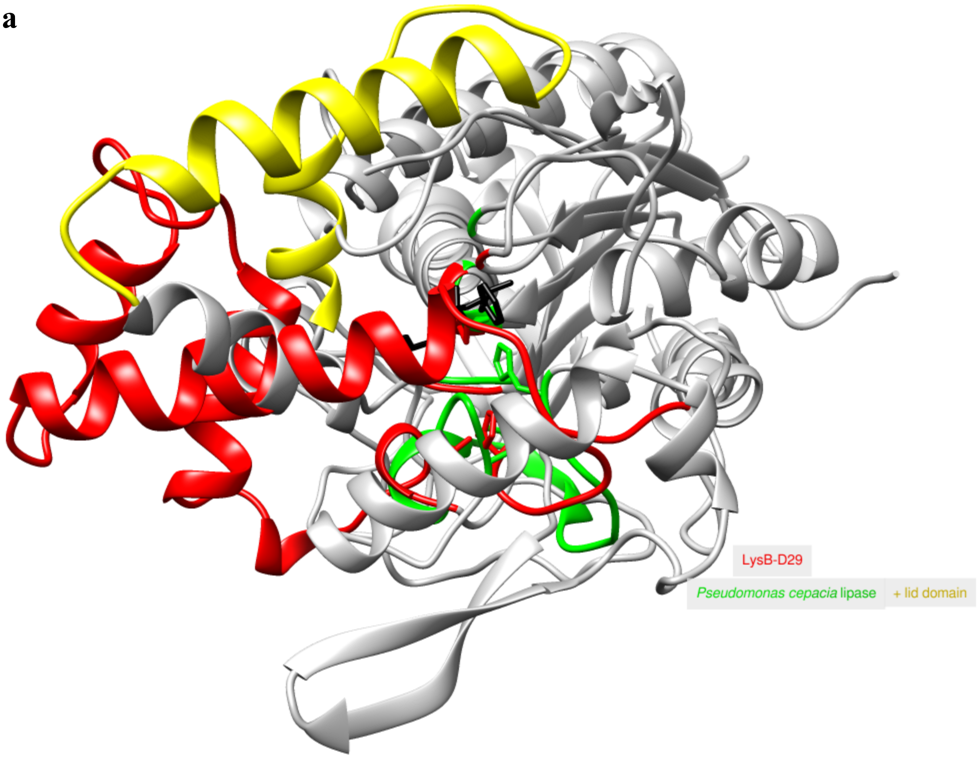
LysB-D29
Pseudomonas cepacia lipase
+ lid domain

b

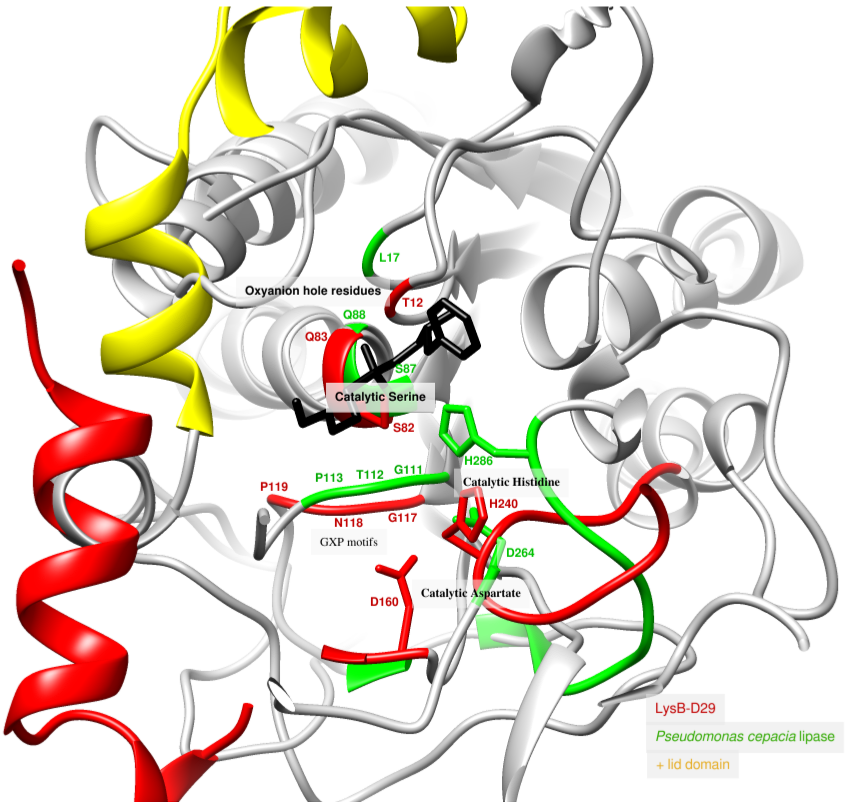
L17
Oxyanion hole residues
T12
Q88
Q83
S87
Catalytic Serine
S82
H286
P119
P113
T112
G111
Catalytic Histidine
N118
G117
H240
GXP motifs
D264
Catalytic Aspartate
D160
LysB-D29
Pseudomonas cepacia lipase
+ lid domain

c

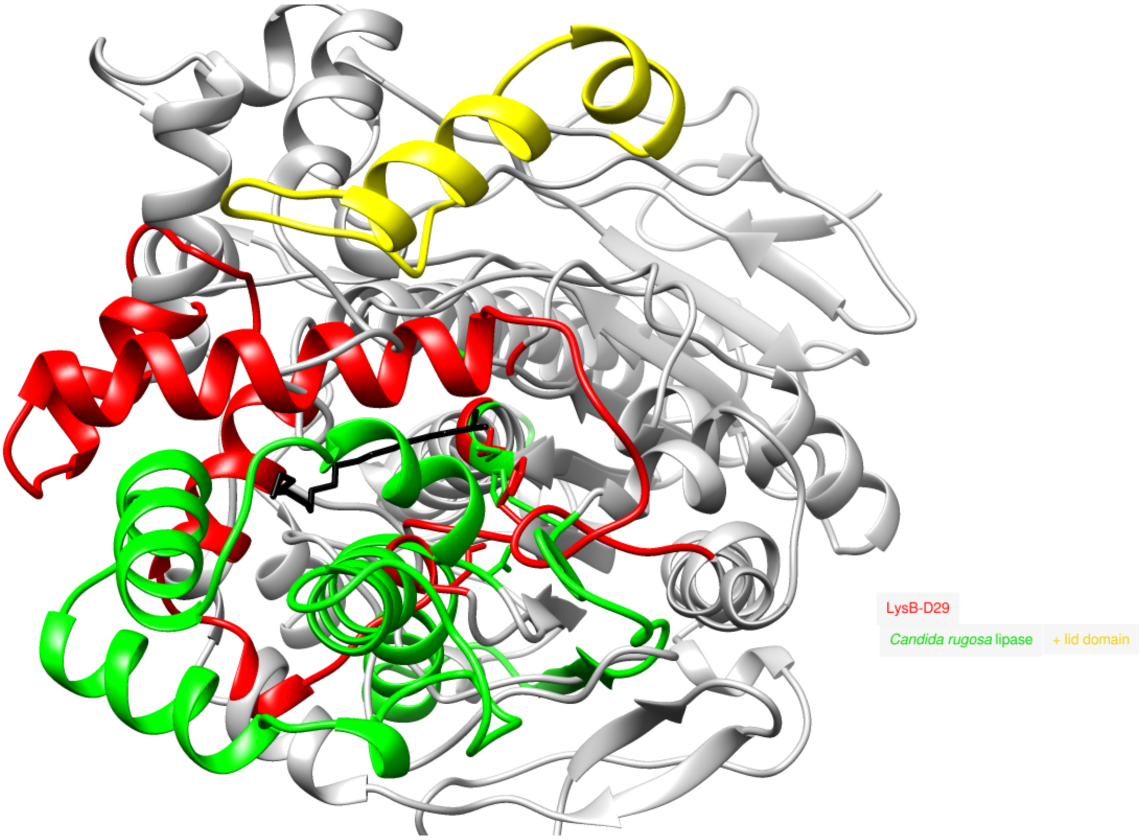
LysB-D29
Candida rugosa lipase
+ lid domain

d

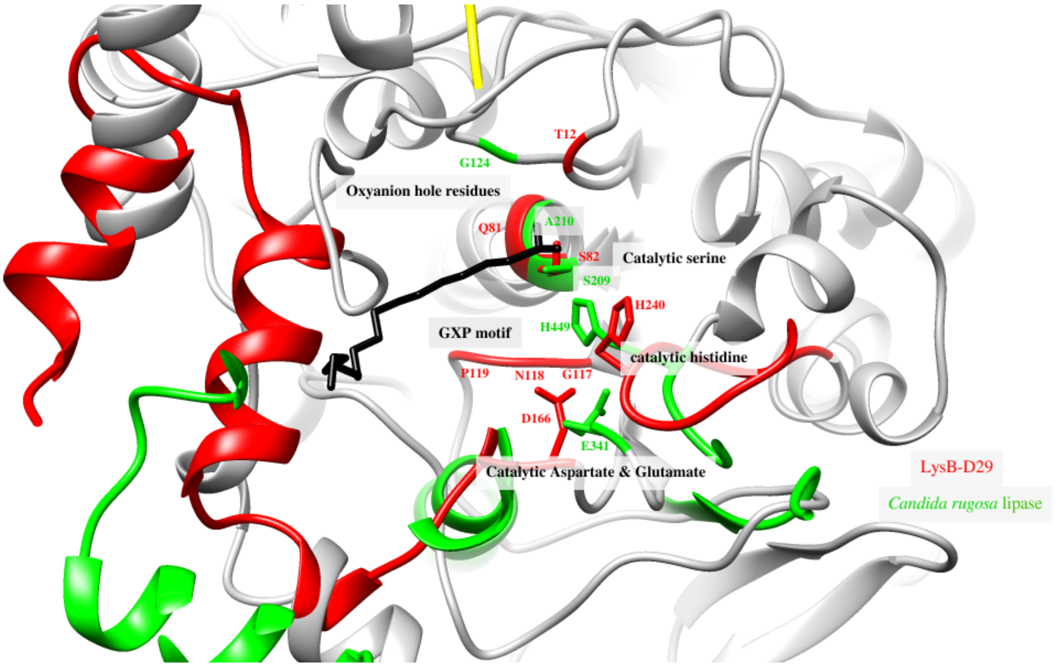
T12
G124
Oxyanion hole residues
Q81
A210
S82
Catalytic serine
S209
H240
H449
GXP motif
catalytic histidine
P119
N118
G117
D166
E341
Catalytic Aspartate & Glutamate
LysB-D29
Candida rugosa lipase

e

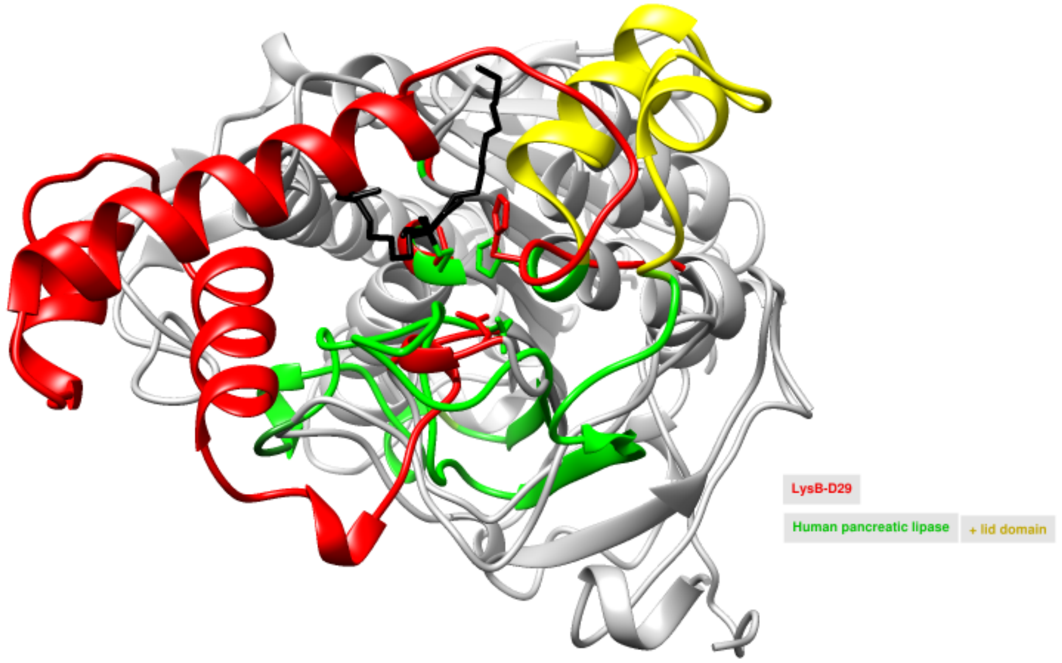

f

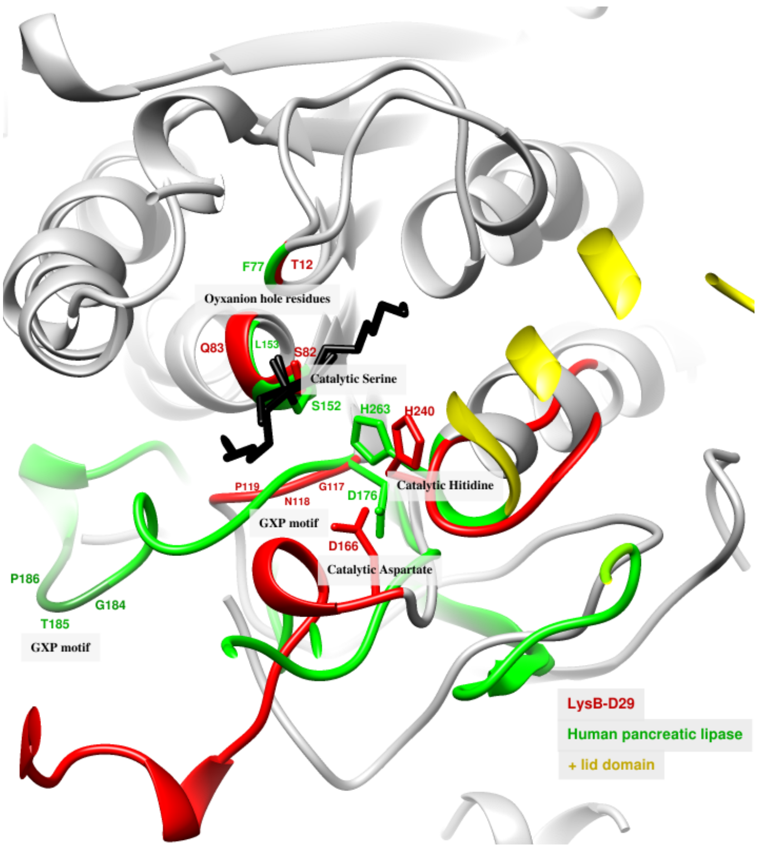

Figure S4. **Total alignment** of three-dimensional structural alignment of 3D structures of LysB-D29 and its relative members of the α/β hydrolase family including: (**a**) *Pseudomonas cepacia* lipase, (**c**) *Candida rugosa* lipase and (**e**) Human pancreatic lipase, showing lid domain (yellow), linker domain of LysB-D29 (red color) and acyl binding site in lipases (green color). **Focus views** of (**b**) alignment of

LysB-D29 to *P. cepacia*, (**d**) alignment of LysB-D29 to *C. rugosa* and (**f**) alignment of LysB-D29 to Human pancreatic lipase, showing triad residues, oxyanion hole residues, GXP residues in red color (LysB-D29) and green color (aligned lipases) and co-crystalized inhibitor molecule (black color).
