# Supplementary material for: Comparative Structural Analysis of Different Mycobacteriophage-Derived Mycolylarabinogalactan Esterases (Lysin B)
Source: Biomolecules. 2019 Dec 27;10(1):45. doi: 10.3390/biom10010045 (PMC7022511; doi:10.3390/biom10010045)
Supplement: Supplementary file 1 [file biomolecules-10-00045-s001.zip › Supplementary/Supp. fig. 5.pdf]

a

```
                                                              Region-1
           ______________________________________________________________________________________________________________
D29        1 ----------------------------------------------------------------------------------------------------M------SKP
SWU1       1 ----------------------------------------------------------------------------------------------------M------SKP
Echild     1 MSLQLG-----------SNGDLVNRWIRVMKARFASYA-----GKLRED----AYFGLDDQAVQREYETRTHQ----------TPDGIVTDGDLAY--LLP------AKP
RedRock    1 MSLKVG-----------SSGEVVSRWIRVMKARFVSYA-----GKLAVD----GYFGLDDAEVQREYERRTGQ----------TPDGIVTDGDLAY--LLP------RKP
Goose      1 MALKVG-----------SSGPLVNAWIRTMNARFESYSKAYDGGPLRED----AYFGYDDAEVQREYERRTGQ----------PVDGEVSDHDLVALGLIT------EKP
Twister    1 MPLKLG-----------SSGELVNAWIRTMNSRFESYSKAYDGGPLRED----AYFGYDDAEVQREYERRTGQ----------PVDGEVSDHDLVALGLIT------EKP
Sheen      1 -----------------------------------------------------------------------------------------------------------MTR
Timshel    1 MILKVG-----------STGQVVSDWQAEMLERYPSYAKAADGGPLKVD----GYFGYDDEAVQVEYEFRTGQ----------PIDREVSQGDLDYLGIKTDS---FKGR
Heldan     1 ----------------------------------------------------------------------------------------------------------MSDR
BabyRay    1 ----------------------------------------------------------------------------------------------------------MSDR
BTCU-1     1 ----------------------------------------------------------------------------------------------------------MSDR
Anubis     1 MPLRVGSND-------ANTGGLVSRWQKTMLARFQAYAKAYDGGPLRVD----GYFGYDDADVQREYERRTHQ----------TVDGEVSDADLRALGLEA------AKR
Bxz2       1 MPLRVGSND-------ANTGGLVSRWQKTMLARYAAYAKAYDGGPLRVD----GYFGYDDADVQREYERRTHQ----------VVDGEVSDADLRALGLEA------AKR
Severus    1 MPLRLG-----------DRNDTVREWRYIMSKRFSGYARALGPLPT-DT----NEFGPRAKSWQEEYERRTGQ----------PVDGEVSDLDLFALGVSVSPHAPGPTR
Zakai      1 --MAVGPDGKWIGYGLGDSGIPVLTARQKLKAKF-SYAKH-----LRET----TLYDNELLPVLIEYQLRKNVEFAKAGKKLLRTDGILDYATQVAIGVVI--PVALVKP
Wildcat    1 ----MRVAGQWVGWGLGDIDPKVQDMKRFLKRKF-SYARES----LDDS----EVYDETMMRVVMQMQANY---------GDLDITGVMNYATQVRSGYLKVEK--PPLP
Ardmore    1 ----MRIDGQYVGLGPGDRSDEIRKIKAFMRRKF-SYAATLA---DT------EFYDEAMTAVVAEMQSRYNT--AGHLRDGLYIPGIINAETKYVMGYLSRPV-IDTRP
Ms6        1 ----MRIDGQYVGLGPGDRSDEIRKIKAFMRRKF-SYAATLA---DT------EFYDEAMTAVVAEMQSRYNT--AGQLRDGLYIPGIINAETKYVMGYLSRPV-IDTRP
Hades      1 ----MRIDGQYVGLGPGDSSEEIRRIKTFMRKKFASYAGDLA---DT------PLYDEQMTAAVAEMQARYNT--AGQLASGLYIPGIVGAETKYVMGYLPRPV-VDTRP
Saal       1 ----MRIDGQYVGLGLGDSSDEIRKIKAFMRRKFASYAGDLA---DT------PLYDEAMTAAVAEMQARYSA--AGQLRAGLYIPGIVGAETKYVMGYLPRPV-VDTRP
Pumpkin    1 ---------------------------------------------------------------------------------------MTTYGELKALRLG----VKYVRH
Graduation 1 -----------MPLKLGDRNPTVRRWREVMAARFAGYARVHGPLPTDTDEFGP---------RAEAWQTEYE------SRTFQPLDGIVSDDD---LRALGIPAPEDTRP
MrMagoo    1 --------MAWIGWQEGMEGLPVLAAIGELRAKF-SYGKQ-----LELT----QKFTPELTQAIKTFQVKKG---------GLRTDGVLDYATQAALGVPE-----ALKP
Dylan      1 ----MRIGGEYVGLGLGDASEEIGRIRDFMRRKFRSYAGNLP---DTRNALGLPLFDEPMTVAVTEMQGRYLQ--SGELRDGLFIFGIINAETKYVMGYLERPPGPDTRP
DS6A       1 --------MTWIGWQQGMAGEPVAAAKRELRRKF-SYAKH-----LDDS----DVFDLELLAVLIEYQQKKNAT---GYTPRLREDGVLDWATQVAIGTVQTGP-PPKAG
Palestino  1 ------------MLKLGSSGLMVSAWTAVMRLRFASYALGVNGQPIKVD----GYFGYDEEKVQKEYQLRTGQ----------FPSGQVSREDLHRLGLLP---------
Obama12    1 ------------MLKLGSNGLMVAAWTAVMRIRFASYALGVNGLPIKVD----GYFGYDEEKVQKEYQRRTGQ----------FPSGQVSREDLHRLGLLP---------
Omega      1 ---------------------------------------------------------------------------------------MSLWPEIR---AMLNGEFYVPAG
Larva      1 ----------------------------------------------------------------------------------------------------------MSKP
Enkosi     1 ----------------------------------------------------------------------------------------------------------MSKP
```

b

```
                 β1      Loop-1        α1             β2                             α2                            β3          α3
D29          5 WLFTVHGTGQPDPLGPGLPADTARDVL----DIYRWQPIGN-YPAA----AFPMWPSVEKGVAELILQIEL------KLDADPYADFAMAGYSQGAIVVGQVLKHHILPP
SWU1         5 WLFTVHGTGQPDPLGPGLPADTARDVL----DIYRWQPIGN-YPAA----AFPMWPSVEKGVAELILQIEL------KLDADPYADFALAGYSQGAIVVGQVLKHHIINP
Echild      73 WLFTVHGTGMPDPLGPGLPADVARDVL----DIYRWQPIGN-YPAA----AFPMKPSYDKAIAELVLQIDL------KLAGN-NDEFSMAGYSQGAIAVAYVLKHEILDP
RedRock     73 WLFTVHGTGMPDPTGPGLPADVARDVL----DIYNWQPIGN-YPAA----AFPMKPSYDKAIAELVLQIDQ------KLAGN-DDEFSMAGYSQGAIAVSYVLKHELINP
Goose       80 WLFTVHGTAQPDPLGPGLPADTAKQVL----DLYRWQPIGN-YPAK----AFPMWPSIMKGYDELVKQIEE------KLSRD-NSDFAMAGYSQGAVVVGQVLKHEIINP
Twister     80 WLFTVHGTAQPDPLGPGLPADTAKQVL----DLYRWQPIGN-YPAK----AFPMWPSIMQGYAELVKQIEE------KLSRD-NSDFALAGYSQGAVVVGQVLKHEIINP
Sheen        4 WLYTVHGTGMPDPFGPGLPADTAREVL----DLYTWQPIGN-YPAA----AFPMWPSIQAGRRELVSQIDS-----------KPGEINMAGYSQGAAVCGQVLKHDIMSP
Timshel     83 WLFTVHGTGMADPFGPGLPADTARDVE----DIYDWQPIGN-YPAA----AFPMWPSITKGVGELRNQIAS-----------KPGEINMAGYSQGAVVCGQVLKHDIMSP
Heldan       5 WLFTVHGTGQPDPLGPGLPADTARQVL----DLYRWQPIGN-YPAS----AFPMWRSVMDGVRELRVQLRR------V---RPEDEVSLAGYSQGAMVVAYVLKYDIMNP
BabyRay      5 WLFTVHGTGQPDPLGPGLPADTARQVL----DLYRWQPIGN-YPAS----AFPMWRSVMDGVRELRVQLRR------V---RPEDEVNLAGYSQGAMVVAYVLKYDIMNP
BTCU-1       5 WLFTVHGTGQPDPLGPGLPADTARQVL----DLYRWQPIGN-YPAS----AFPMWRSVMEGVRELRVQLRR------V---RPEDTVNLAGYSQGAMVVAYVLKYDIMNP
Anubis      84 WLFTVHGTGMPDPLGPGLPADTARAVL----DKYTWQPIGN-YPAA----VFPMWPSIQAGRTELRNQIAA-----------KAGEVNLAGYSQGAVVVGQVLKHDIMDP
Bxz2        84 WLFTVHGTGQADPLGPGLPADTARAVL----DKYTWQPIGN-YPAR----AFPMWSSIMDGARELRSQIAS-----------KSGEVNLAGYSQGAVVVGQVLKHDIMDP
Severus     85 WLFTVHGTGMADPLGPGLPADTARACL----DKYTWQPIGN-YPAS----VWPMWPSIQKGRTELRAQIAA-----------KQGEINMAGYSQGAVVVGQVLKHDIIDP
Zakai       97 LLFTVHGTGQPVPDGPGLPADTARAVL----DKWEWQPIGN-YPAD----AFPMWPSILEGVAELRDQIRK------ALAANPRRKLGFAGYSQGAVVVSLVLKYDFMAQ
Wildcat     87 TLYTVHGTGVSMWD--GPPADCARRLL----DKYRWQPVGN-YPAS----AFPMWPSIQAGCLELNRLIES-----------TPGKFTLAGYSQGAMVTSIVYKYDLLDP
Ardmore     94 VLFTVCGTGVPWWV--GPDADTARAVE----DQYLWQPIGY--PAA----PFPMGRSITAGITEAHNQANR------WRERIETHGTALAGYSQGAVVLSELWMNHIAPE
Ms6         94 VLFTVCGTGVPWWV--GPDADTARAVE----DQYLWQPIGY--PAA----PFPMGRSITAGITEAHNQANR------WRERIETHGTALAGYSQGAVVLSELWMNHIAPE
Hades       95 VLITVCGTGVPWWI--GPDADTARAVE----DKYLWQPIGY--PAA----PFPMGKSITAAITETHNQANR------WRQRIETHGAALAGYSQGAVVVSELWMNHIAPE
Saal        95 VLITVCGTGVPWWI--GPDADTARAVE----DKYLWQPIGY--PAA----PFPMGKSIAAAITETHNQANR------WRERIETHGAALAGYSQGAVVVSELWMNHIAPE
Pumpkin     20 TLFTVAGTWADMWS--GYPADVARLVD---EDLFRWQPVW--YPASFGPVGNPLGRSYQESVQDGVKELI-------RLINATPGTFALVGYSQGAEVVSRVLLEI---L
Graduation  82 VLLTVSGTGVPWWI--GPDADVARRLG----DVYLWRPVGPPYTAQ----AFPMGPSVANGVTEATRILEE------ERQRIERYGLSMIGYSQGAIVTSELWEYHIKPV
MrMagoo     79 WVFTVAGTGAGWD--AGYPADVARAVL----DLFRWWGI-V-YPAK----PFPMGDSVDVGIAELLKQMKL------KLDRYPAAKFVLIGYSQGAIVTSMVWKRYIKGK
Dylan      102 LFITVCGTGVPWWV--GPDADTARACE----DKLFWQPIGY--PAK----NFPMGKSIAAGIDQCHVQFNRADPGFMHRQRIERNGVVLGGYSQGAVVVSELWENNIKPA
DS6A        89 TLFTVQGTGVDMWT--GPPADTARAVE----GDWEWQPIGN-YPAS----PFPMWASIWQGIEELRFQIRR------HATEKPGKAIALAGFSQGAVVVSWVYKWDIALP
Palestino   76 TLLSIHGTGQADPFGIGYPADIARRVL----DLYWWQPVGN-WPAK----AVPMNGSVDAGERECVRLISN--------PLIVPGPTAFVDYSQGSVIGGRVRNRM----
Obama12     76 TLISIHGTGQADPFGIGYPADIARRVL----DLYWWQPLGN-YPAK----AVPMNGSVDQGEREGVRLISD--------PLIVPGPTAFVDYSQGSVIGGRLRNRM----
Omega       21 TIITANGTGVPDGAGPGFGADLARELERVAEGMWIWKWIN--YPAA----VMPMRPSINTLRLALKEMIRT-----------TPGKIVLSAYSQSAVAFAYVWRDDILNP
Larva        5 MLLTASGTGADMWT--GYPADLARRV----EDLYYFQPIY--YGPNGIPATVPMGQSAKSGVDMGVSLVLQAE---ARPAREVPDGYALCGYSQGGMVTSALLDEF---R
Enkosi       5 VLLTASGTGADMWS--GYPADLARRV----EDLYYFQPIY--YGPGGIPATVPMGASAQSGVDMGVRLVLECE---ARPSRDVPDGYALCGYSQGGMVVSALLDEF---R
                . :  **        *  ** *:       .   :  :             *:  *                               :  :**..   . :
```

c

```
                  α4     β4                  Loop-2                           α5          β5     Loop-3   α6 L-4 α7            α8
D29          100 TGRLHRF--LHRLKKVIFWGNPMRQKGFAHSDEWIHPVAAPDTL-----GILE-DRLENLE-----QYGFEVRDYAHD------GDMYASIKED--------DMHEYEVA
SWU1         100 RGRLHRF--LHRLRKVIFWGNPMRQKGFAHTDEWIHPVAASDTM-----GILE-DRLENLE-----QYSFEVRDYAHD------GDMYASIKED--------DMHEYEVA
Echild       167 KGRLHKY--VNRLKKVVMWGNPMRQKGFAHFDEWIHPVAAPDTM-----GILE-DRLENLEQA-MQQYGFEVRDYAHD------GDMYASIKED--------DMHEYEVA
RedRock      167 KGRLHKY--VHRLKKVVLWGNPMRQKGFAHFDEWIHPVAGPDTM-----GILE-DRLEALEQA-MKVYGFEVRDYAHD------GDMYASIKED--------DMHEYEVA
Goose        168 KGRLHKY--LHRLKKVVFWGNPMRQKGIAHFDEWIHPVAGPDTM-----GILE-DRLENLE-----QYDFEVRDYAHD------GDMYASIKED--------DMHEYQVA
Twister      168 NGRLHKF--LHRLKKVVFWGNPMRQKGIAHFDEWIHPVAGPDTM-----GILE-DRLENLE-----QYDFEVRDYAHD------GDMYASIKED--------DMHEYQVA
Sheen         94 TGRLHHR--LGDVRKVVFWGNPMRQQGIAHSDEWVHPVASPDTM-----GILE-DRLEGLE-----NAPFEVRDYAHQ------GDMYASIKAD--------DMHEYQVA
Timshel      173 SGSLHHR--LKDVRKVVFWGNPMRQQGIAHTDEWIHPVASPSTM-----GILE-DRLEGLE-----DAPFEVRDYAHE------GDMYASIKAD--------DMHEYEVA
Heldan        97 AGEFHYL--LHQVRKVVFWGNPMRQRGIAHDDRWIHRIAAPDTH-----GIME-DRLEGLE-----HAPFEVRDYAHD------EDMYASIRDD--------DMHEYQIA
BabyRay       97 AGEFHYL--LHQVRKVVFWGNPMRQQGIAHDDRWIHRIAAPDTH-----GIME-DRLEGLE-----HAPFEVRDYAHD------EDMYTSIRDD--------DMHEYQIA
BTCU-1        97 AGEFHYL--LHQVRKVVFWGNPMRQRGIAHDDRWIHRIAAPDTH-----GIME-DRLEGLE-----HAPFEVRDYAHD------EDMYASIRDD--------DMHEYQIA
Anubis       174 KGSLHHR--LGDVRKVVMWGNPMRQKGIAHDDRWIHKVAGPDTY-----GILE-DRLEGLD-----KAPFEVRDYAHE------DDMFASVRDD--------DIHEYQIA
Bxz2         174 KGSLHHR--LGDVRKVVLWGNPMRQRGIAHFDEWIHPVAGPDSY-----GILD-DRLEGLE-----KAPFEIRDYAHA------GDMYASITDG--------DKDEYKIA
Severus      175 AGPLHHR--LGDVRKVVFWGNPMRQQGIQHDDQWIHEIAPADTH-----GILTFDLLEGLE----RFPHIQVRDYAHK------GDMYACNTDD--------QAAEYKRA
Zakai        192 GGEFHDQYEAGQVLAVVTWGNPMRERGVQHGDGWKE-TAPADTE-----GILE-DRLEGTP--------AFWREYAHK------GDMYAACELTG------VQRGDNKRA
Wildcat      175 MGRLHHR--LPDFMGGVTWGNPMREMGKAWTDGV-GAVAGQNNG-----GIAE-DRLVGTP--------WNWRDYAHK------GDLYTDCEFD--------DEGEYKRS
Ardmore      186 DGSLRWM--KPHVRKAVTWGNPNRELGHVWADHGGSPMAPSNTQ-----GVSS-N-------G-MRNTPDWWRDYAHQ------GDLYACTEPG--------DTQEVRNA
Ms6          186 DGSLRWM--KPHVRKAVTWGNPNRELGHVWADHGGSPMAPSNTQ-----GVSS-N-------G-MRNTPDWWRDYAHQ------GDLYACTEPG--------DTQEVRNA
Hades        187 TGSLHWM--KPHIEKAVTWGNPNRELGHVWADHGGSPMAPSNTQ-----GVSS-N-------G-MRDTPPWWRDYAHQ------GDLYACTEPG--------DTQEVRNA
Saal         187 DGSLHWM--KPHIEKAVTWGNPNRELGHVWADHGGSPRAPSNTQ-----GVSS-N-------G-MRDTPPWWRDYAHQ------GDLYACTEPG--------KSGEIITS
Pumpkin      113 FGSLRHR--LKDFIGGGCFGNPYRAKGVSYPGSGL----PTSGH-----GIAP-VNLAPDI-----LPAEMWEEWWNE------GDLYAQNLDG--------DSGEMKTA
Graduation   176 TGRLHWV--KDHVRGAVTFGNPMRETGKVWPNPGGQL-PSAKSH-----GIAD-Q-------L-MVDTPDWWRNYAHK------GDLYTDCE-G--------DSGEMKTA
MrMagoo      173 --DL-----EKRIIGSITFGNPCRELGVANGNVAE-GIPIPEGR-----GIGD-DRLQSTP--------EWWYDFAHGANSPFGRDIYTDTPDD--------DTGEMMTA
Dylan        200 NGSLHWA--KDYVVKAVTWGNPNREVGAVWPDYGGSPMASLTSQ-----GVSS-T-------G-MRDTPSWWRNYAHA------GDLYAAAEPG--------DSQQDKNA
DS6A         182 GGMLHDL--LPQVAAGVTWGNPMAEKGKFHGNRFAG-WPVPAGM-----GINR-DRLENTP--------ALWLDFGHGRNSPWGQDIYCDRPDN--------LAGQDMEL
Palestino    169 ----RRKELRGELVAAASFGNPMRLRGHYAGNVD------PGGE-----GIDPRQELAAE---------PFRVELAAK------GDLYTTCPGG--------QSGEMERA
Obama12      165 ----RRKELRGELVAAASFGNPMRLRGHYAGNVD------PGGE-----GIDPRQELAAE---------PFRIELAAK------GDLYTTCPGG--------QSGEMERA
Omega        110 DGELHDR--LDDIVAVILYGNPVRAPGIAYGNELGGQSAPGKLNGHVTGGIAGPDCLRPEECVHPVTGRRIVLDFANA------GDLYAAAPVGAEPWVKETEVGHNETL
Larva        101 TGRLKSV--GHKLMAGSTFGNPWRELDAD------------GGR-----GISD-RRI---A-----GTPDFWVDEFDP------GDIYANVPNN--------DVGADMTA
Enkosi       101 TGRLRHL--NGKLMAGSTFGNPWRELDSN------------GGR-----GISD-RRI---V-----GTPDFWVDEFDP------GDIYANVPNN--------DVGDDMTA
                               .      :***    .                    *:                      :         *::
```

d

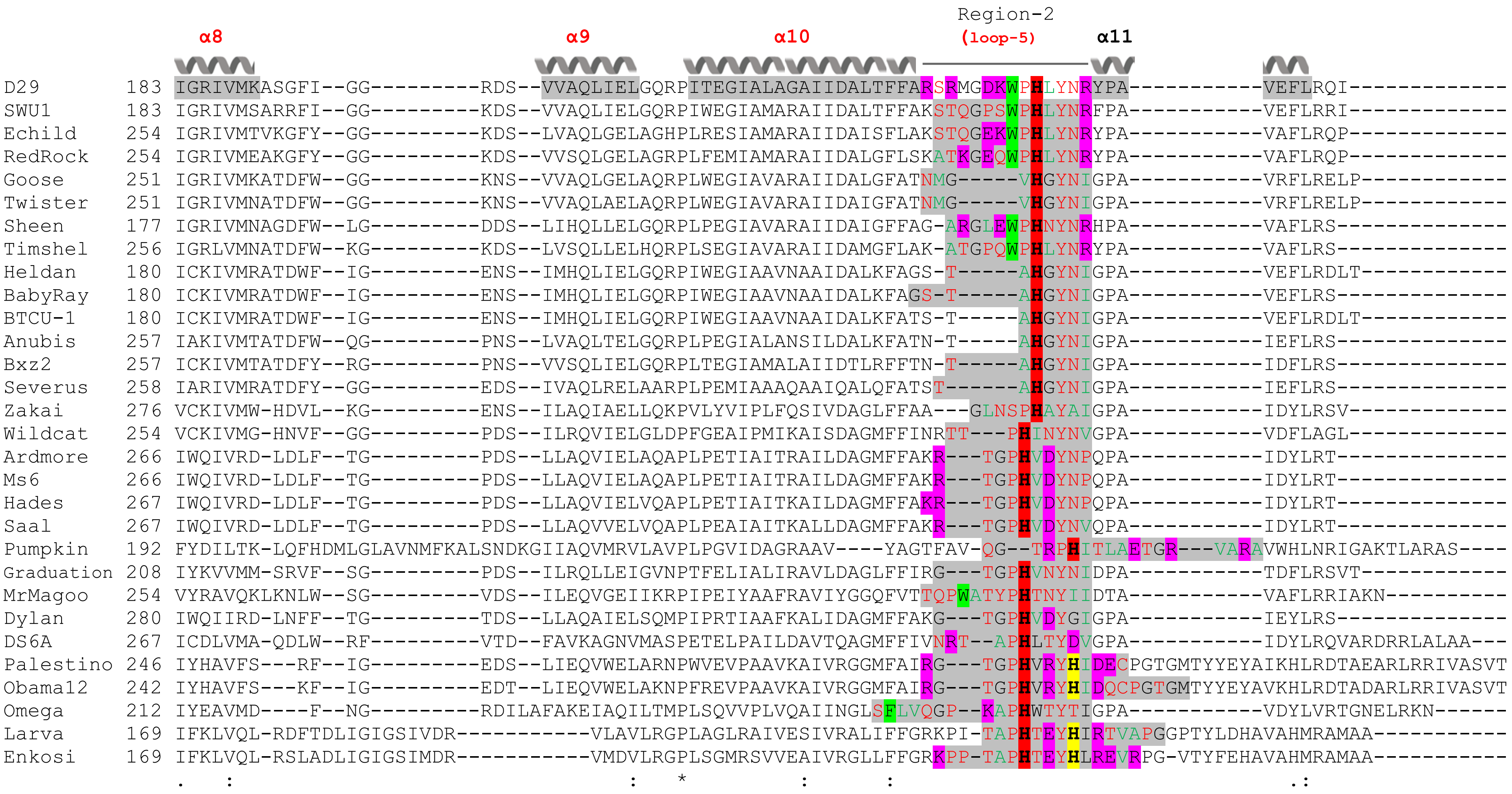

Figure S3: Multiple sequence alignment of LysB-D29 with its homologous lysB proteins. Identical residues are labeled by *Asterisk*, conserved residues labeled by *colon* and *full stop* indicates semi-conserved residues. Residues forming the active site are shaded in red color. Extra His residues are highlighted in yellow color. Oxyanion hole and GXP residues are shaded in pink. Residues forming β-strands and α-helices are highlighted in gray. Region-1 constitutes extra N-terminal residues. Region-2 constitutes residues forming hypervariable loop-5. Loops, β-strands and α-helices forming linker domain are in red. Residues forming loop-5 in each LysB model are highlighted in gray. Polar non-charged residues are in red, polar charged residues are highlighted in pink. Non-polar aliphatic residues are in green, non-polar aromatic residues are highlighted in green.
