# Supplementary material for: Comparative Structural Analysis of Different Mycobacteriophage-Derived Mycolylarabinogalactan Esterases (Lysin B)
Source: Biomolecules. 2019 Dec 27;10(1):45. doi: 10.3390/biom10010045 (PMC7022511; doi:10.3390/biom10010045)
Supplement: Supplementary file 1 [file biomolecules-10-00045-s001.zip › Supplementary/Supp. fig. 6.pdf]

LysB-D29

PROCHECK

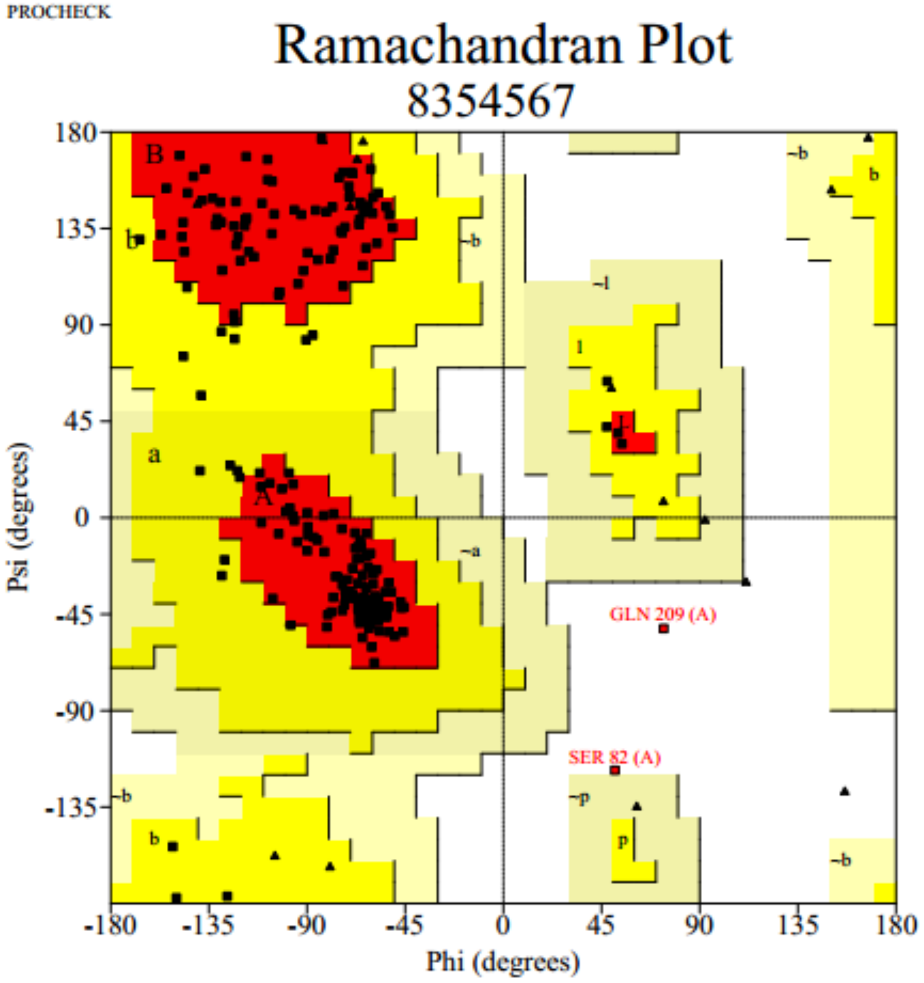

Plot statistics

Residues in most favoured regions [A,B,L]	184	88.0%
Residues in additional allowed regions [a,b,l,p]	23	11.0%
Residues in generously allowed regions [~a,~b,~l,~p]	0	0.0%
Residues in disallowed regions	2	1.0%
	----	------
Number of non-glycine and non-proline residues	209	100.0%
Number of end-residues (excl. Gly and Pro)	2	
Number of glycine residues (shown as triangles)	23	
Number of proline residues	18	

Total number of residues	252	

Based on an analysis of 118 structures of resolution of at least 2.0 Angstroms and R-factor no greater than 20%, a good quality model would be expected to have over 90% in the most favoured regions.

8354567_01.ps

1- LysB-Chy5

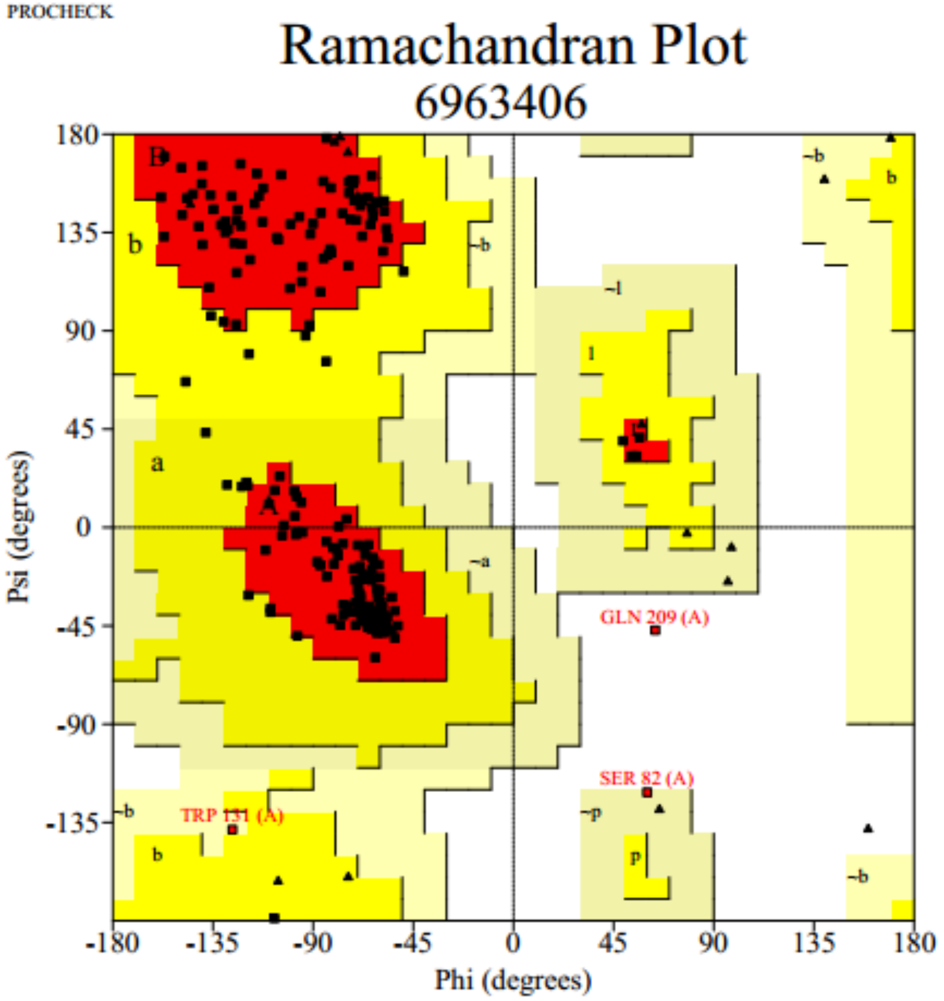

Plot statistics

Residues in most favoured regions [A,B,L]	194	91.9%
Residues in additional allowed regions [a,b,l,p]	14	6.6%
Residues in generously allowed regions [~a,~b,~l,~p]	2	0.9%
Residues in disallowed regions	1	0.5%
	----	------
Number of non-glycine and non-proline residues	211	100.0%
Number of end-residues (excl. Gly and Pro)	2	
Number of glycine residues (shown as triangles)	23	
Number of proline residues	18	

Total number of residues	254	

Based on an analysis of 118 structures of resolution of at least 2.0 Angstroms and R-factor no greater than 20%, a good quality model would be expected to have over 90% in the most favoured regions.

6963406_01.ps

2- LysB-SWU-1

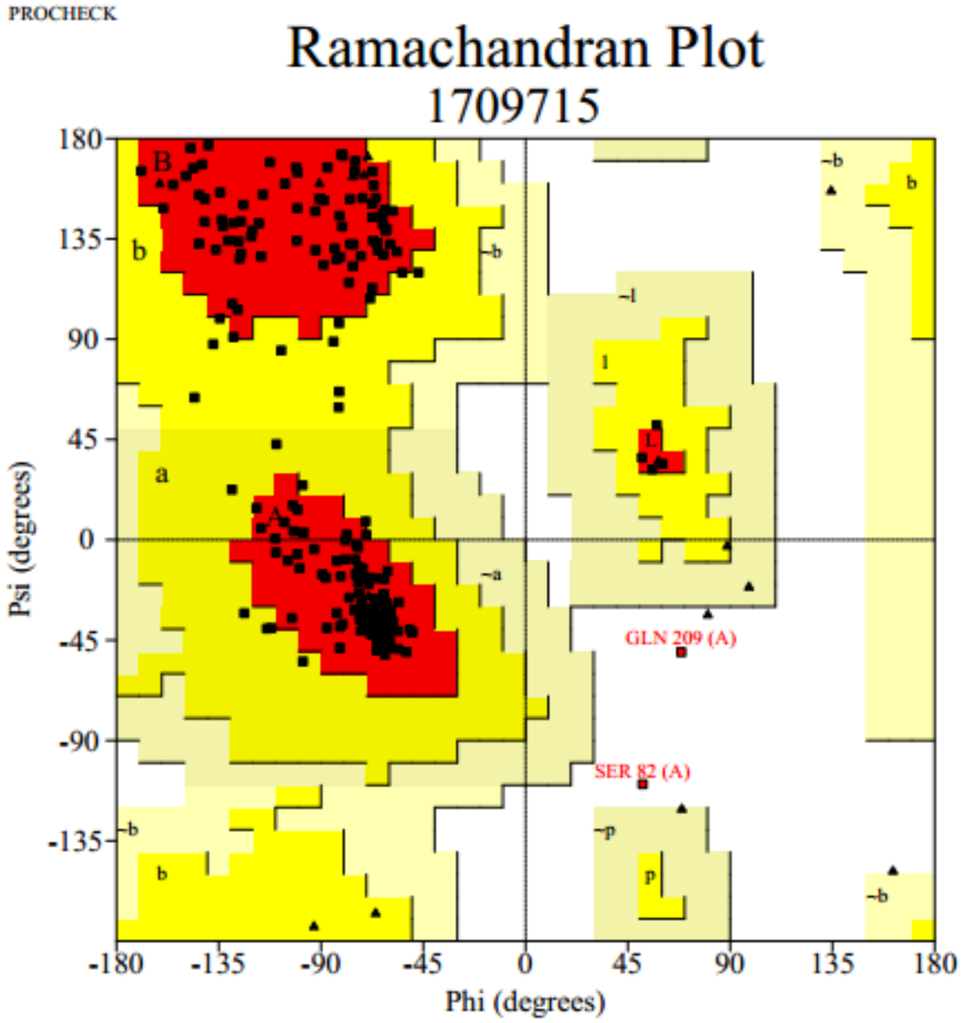

Plot statistics

Residues in most favoured regions [A,B,L]	194	90.2%
Residues in additional allowed regions [a,b,l,p]	19	8.8%
Residues in generously allowed regions [~a,~b,~l,~p]	0	0.0%
Residues in disallowed regions	2	0.9%
	----	------
Number of non-glycine and non-proline residues	215	100.0%
Number of end-residues (excl. Gly and Pro)	2	
Number of glycine residues (shown as triangles)	20	
Number of proline residues	17	

Total number of residues	254	

Based on an analysis of 118 structures of resolution of at least 2.0 Angstroms and R-factor no greater than 20%, a good quality model would be expected to have over 90% in the most favoured regions.

1709715_01.ps

3- LysB-Echild

PROCHECK

Ramachandran Plot

6412088

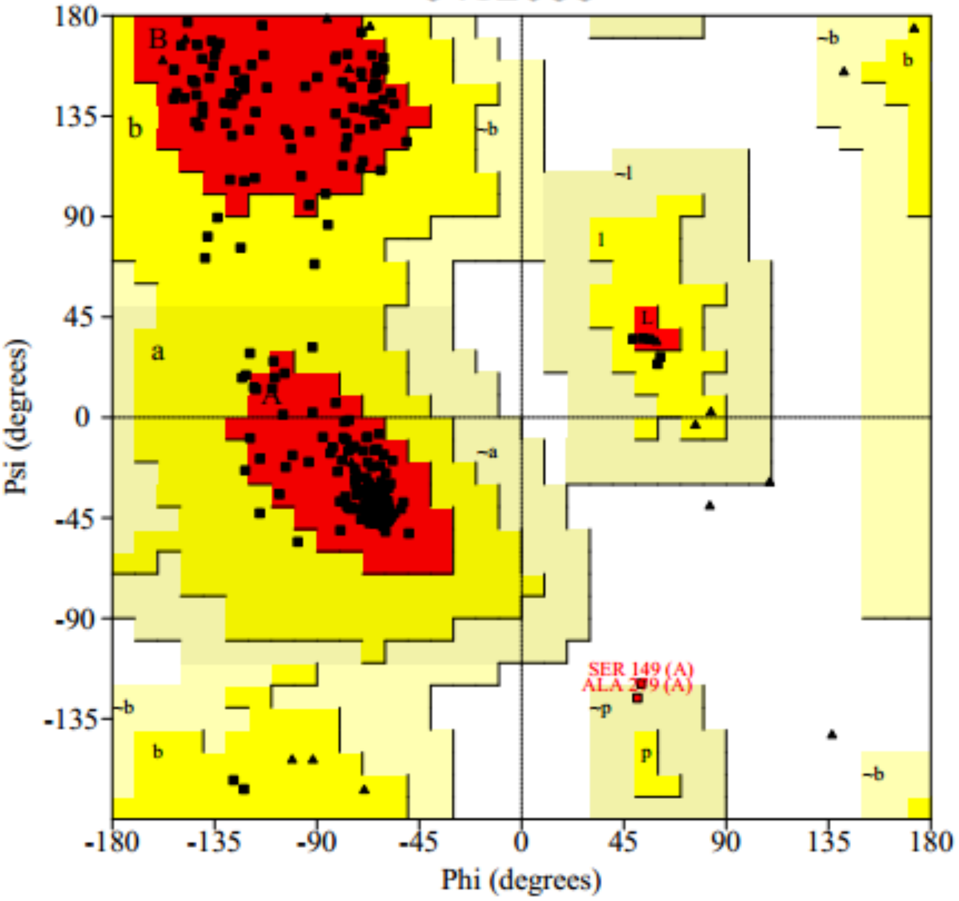

Plot statistics

Residues in most favoured regions [A,B,L]	205	91.1%
Residues in additional allowed regions [a,b,l,p]	18	8.0%
Residues in generously allowed regions [~a,~b,~l,~p]	1	0.4%
Residues in disallowed regions	1	0.4%
	----	------
Number of non-glycine and non-proline residues	225	100.0%
Number of end-residues (excl. Gly and Pro)	1	
Number of glycine residues (shown as triangles)	22	
Number of proline residues	18	

Total number of residues	266	

Based on an analysis of 118 structures of resolution of at least 2.0 Angstroms and R-factor no greater than 20%, a good quality model would be expected to have over 90% in the most favoured regions.

6412088_01.ps

4-LysB-Redrock

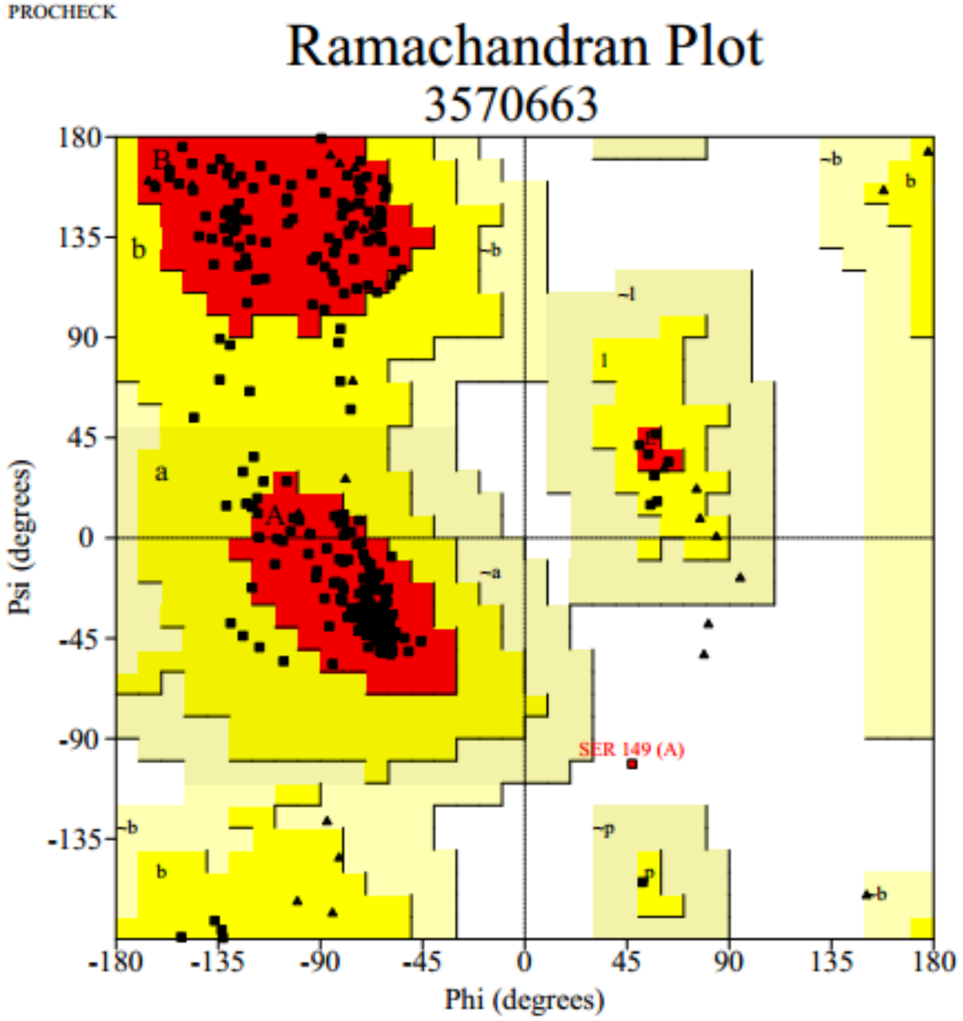

Plot statistics

Residues in most favoured regions [A,B,L]	241	88.0%
Residues in additional allowed regions [a,b,l,p]	32	11.7%
Residues in generously allowed regions [~a,~b,~l,~p]	0	0.0%
Residues in disallowed regions	1	0.4%
	----	------
Number of non-glycine and non-proline residues	274	100.0%
Number of end-residues (excl. Gly and Pro)	1	
Number of glycine residues (shown as triangles)	31	
Number of proline residues	19	

Total number of residues	325	

Based on an analysis of 118 structures of resolution of at least 2.0 Angstroms and R-factor no greater than 20%, a good quality model would be expected to have over 90% in the most favoured regions.

3570663_01.ps

5-LysB-Goose

PROCHECK

Ramachandran Plot

8623335

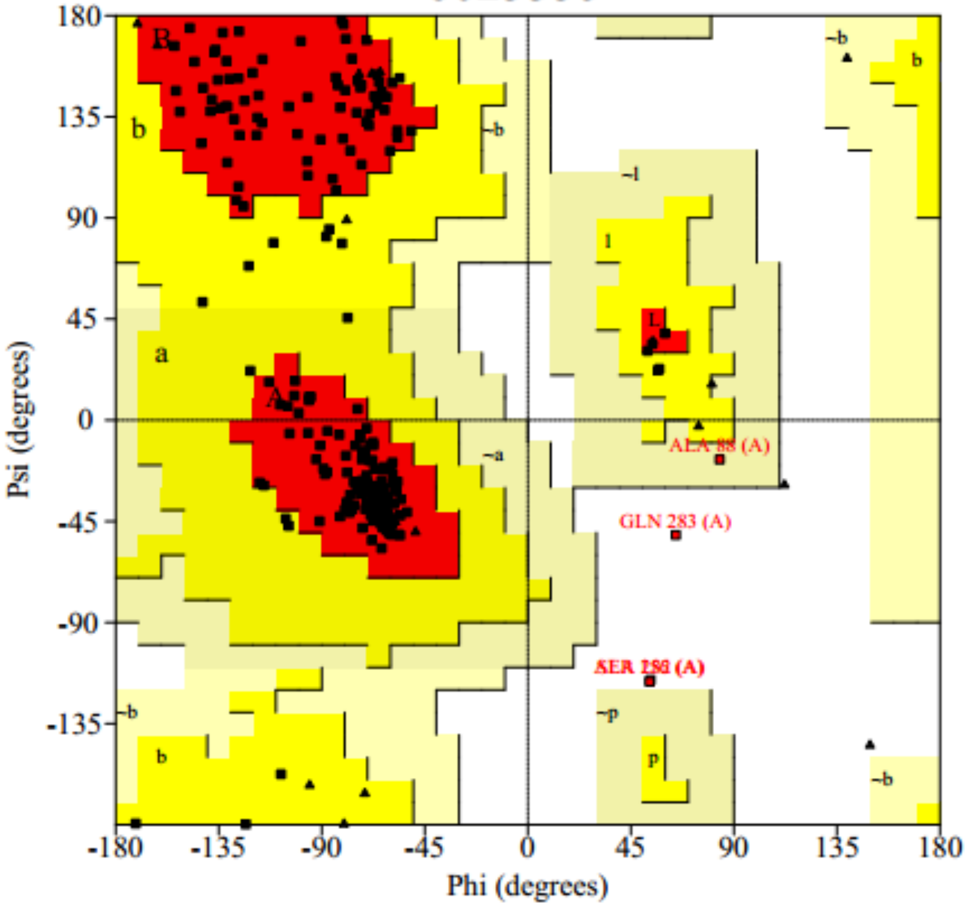

Plot statistics

Residues in most favoured regions [A,B,L]	197	90.8%
Residues in additional allowed regions [a,b,l,p]	16	7.4%
Residues in generously allowed regions [~a,~b,~l,~p]	1	0.5%
Residues in disallowed regions	3	1.4%
	----	------
Number of non-glycine and non-proline residues	217	100.0%
Number of end-residues (excl. Gly and Pro)	1	
Number of glycine residues (shown as triangles)	24	
Number of proline residues	16	

Total number of residues	258	

Based on an analysis of 118 structures of resolution of at least 2.0 Angstroms and R-factor no greater than 20%, a good quality model would be expected to have over 90% in the most favoured regions.

8623335_01.ps

6- LysB-Twister

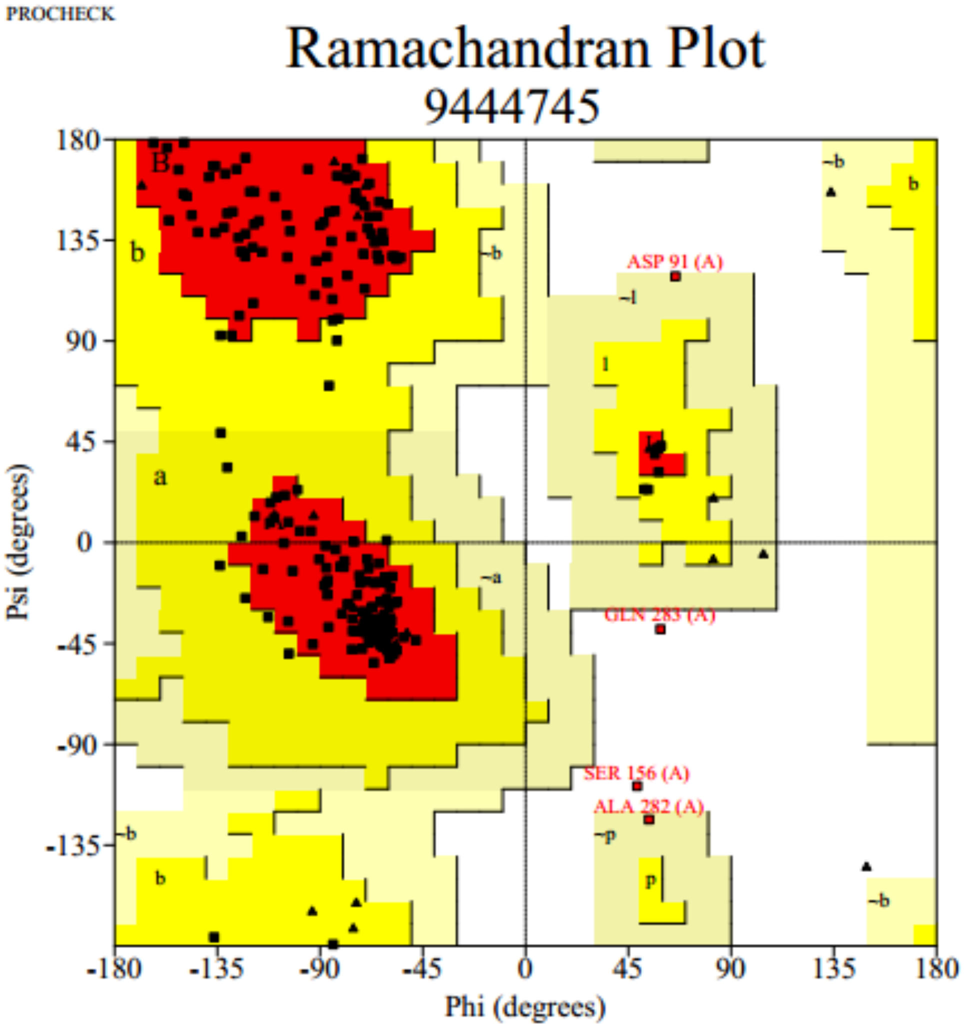

Plot statistics

Residues in most favoured regions [A,B,L]	196	89.9%
Residues in additional allowed regions [a,b,l,p]	18	8.3%
Residues in generously allowed regions [~a,~b,~l,~p]	2	0.9%
Residues in disallowed regions	2	0.9%
	----	------
Number of non-glycine and non-proline residues	218	100.0%
Number of end-residues (excl. Gly and Pro)	1	
Number of glycine residues (shown as triangles)	23	
Number of proline residues	16	

Total number of residues	258	

Based on an analysis of 118 structures of resolution of at least 2.0 Angstroms and R-factor no greater than 20%, a good quality model would be expected to have over 90% in the most favoured regions.

9444745_01.ps

7- LysB-Sheen

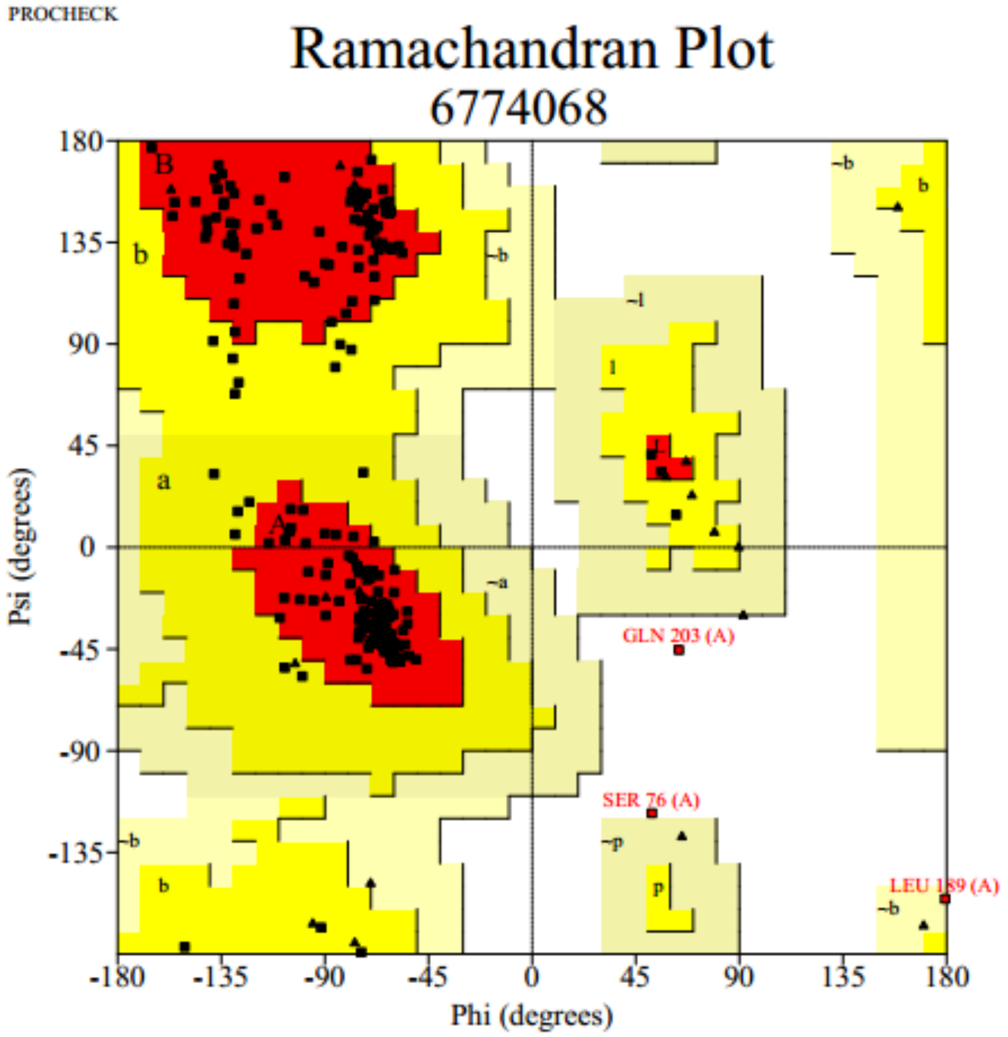

Plot statistics

Residues in most favoured regions [A,B,L]	176	87.6%
Residues in additional allowed regions [a,b,l,p]	22	10.9%
Residues in generously allowed regions [~a,~b,~l,~p]	1	0.5%
Residues in disallowed regions	2	1.0%
	----	------
Number of non-glycine and non-proline residues	201	100.0%
Number of end-residues (excl. Gly and Pro)	2	
Number of glycine residues (shown as triangles)	25	
Number of proline residues	18	

Total number of residues	246	

Based on an analysis of 118 structures of resolution of at least 2.0 Angstroms and R-factor no greater than 20%, a good quality model would be expected to have over 90% in the most favoured regions.

6774068_01.ps

8- LysB-Timshel

PROCHECK

Ramachandran Plot

9538420

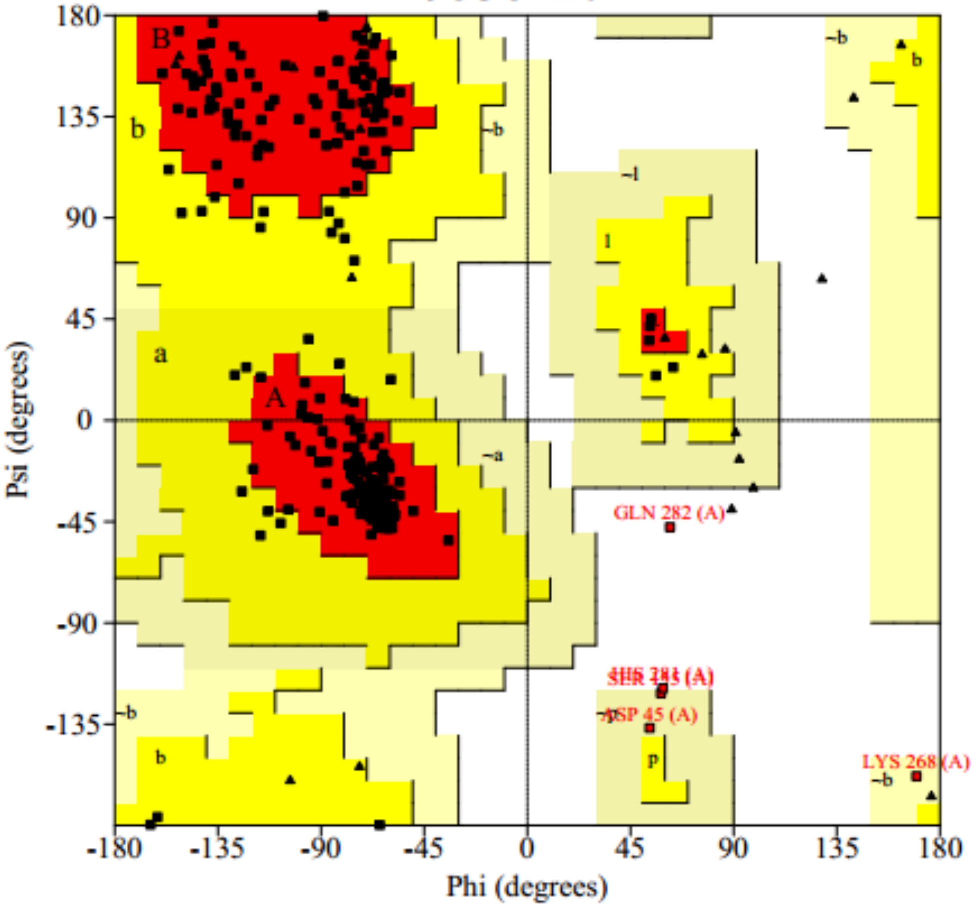

Plot statistics

Residues in most favoured regions [A,B,L]	241	88.9%
Residues in additional allowed regions [a,b,l,p]	25	9.2%
Residues in generously allowed regions [~a,~b,~l,~p]	3	1.1%
Residues in disallowed regions	2	0.7%
	----	------
Number of non-glycine and non-proline residues	271	100.0%
Number of end-residues (excl. Gly and Pro)	2	
Number of glycine residues (shown as triangles)	32	
Number of proline residues	20	

Total number of residues	325	

Based on an analysis of 118 structures of resolution of at least 2.0 Angstroms and R-factor no greater than 20%, a good quality model would be expected to have over 90% in the most favoured regions.

9538420_01.ps

9- LysB-Heldan

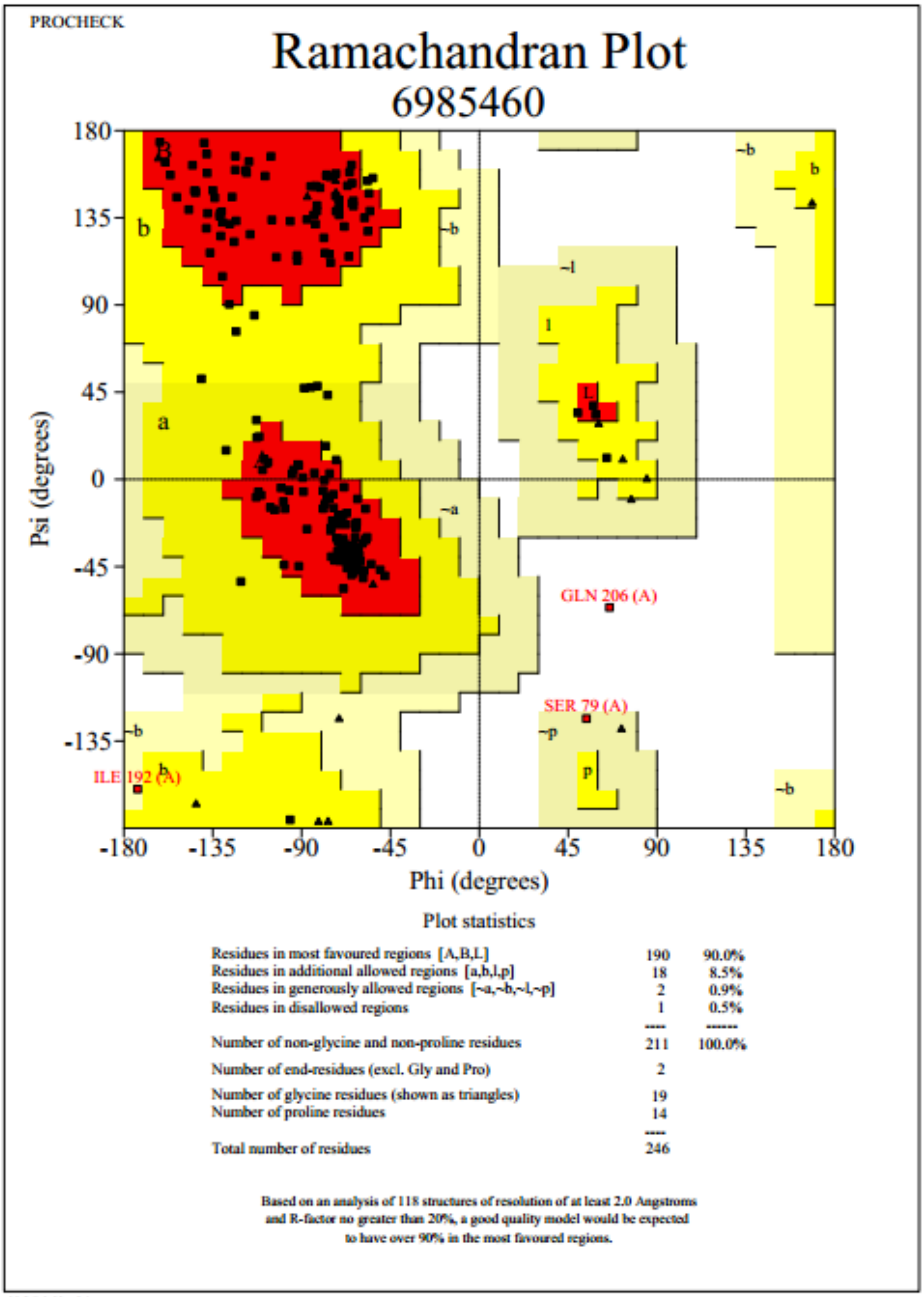

6985460_01.ps

10- LysB-Babyray

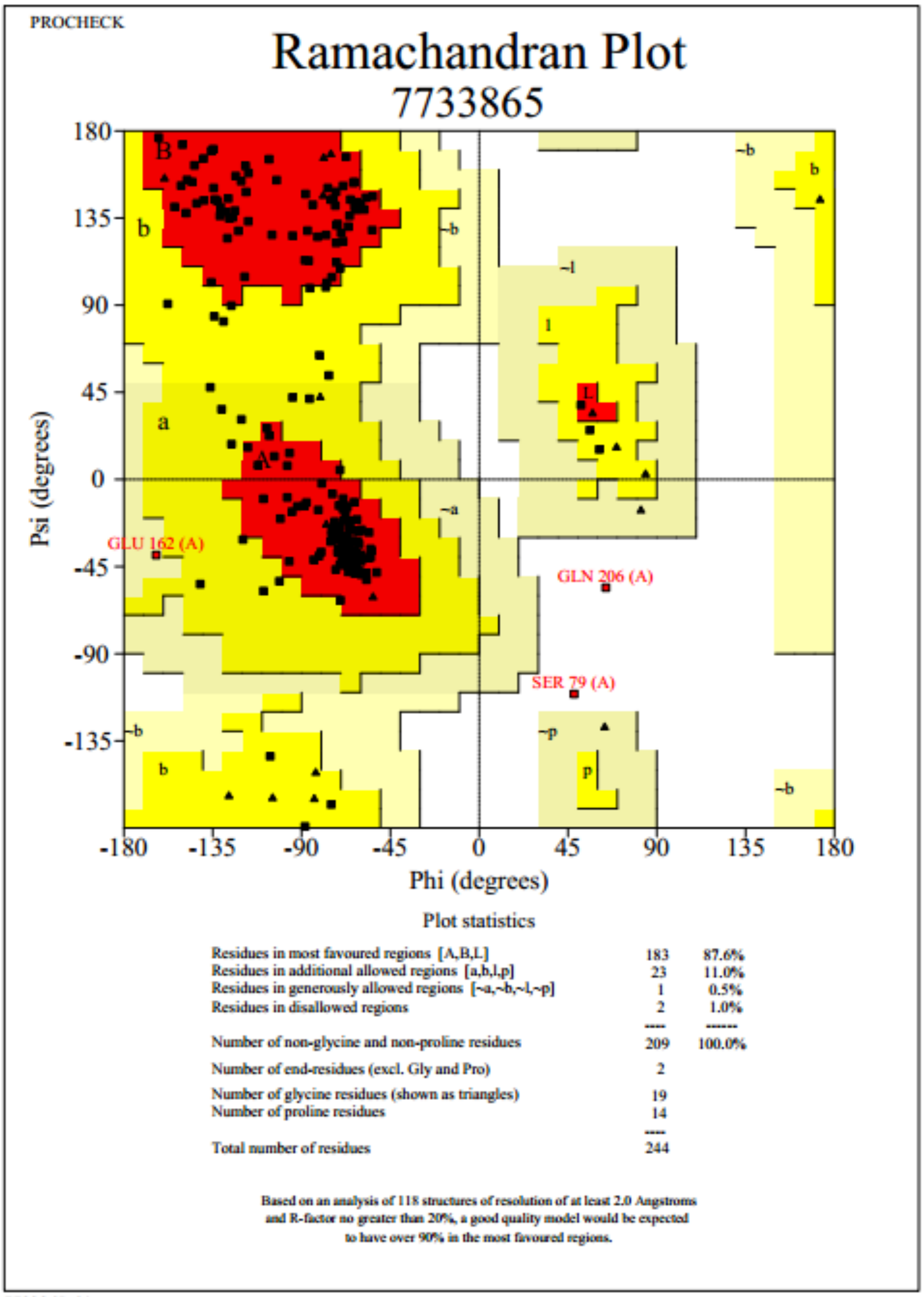

7733865_01.ps

11- LysB-BTCU-1

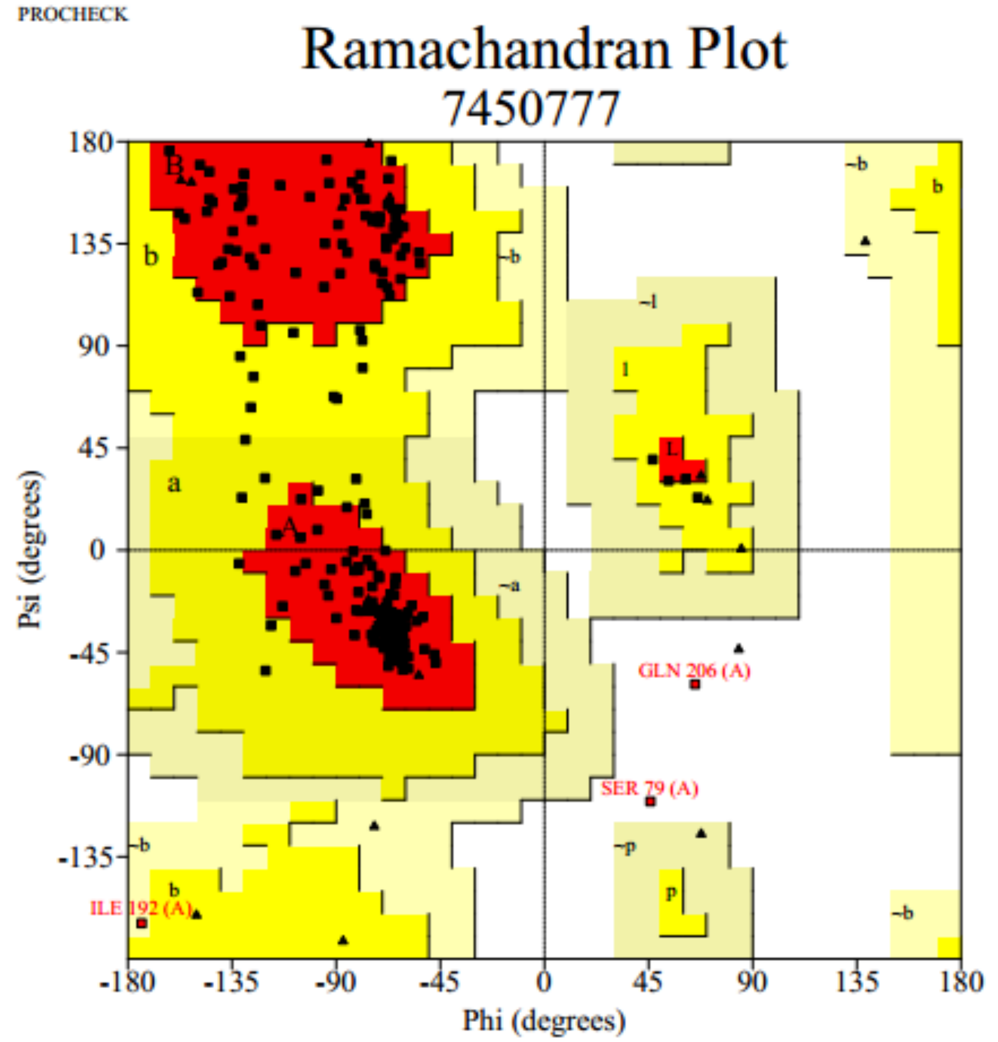

Plot statistics

Residues in most favoured regions [A,B,L]	189	89.2%
Residues in additional allowed regions [a,b,l,p]	20	9.4%
Residues in generously allowed regions [~a,~b,~l,~p]	1	0.5%
Residues in disallowed regions	2	0.9%
	----	------
Number of non-glycine and non-proline residues	212	100.0%
Number of end-residues (excl. Gly and Pro)	2	
Number of glycine residues (shown as triangles)	18	
Number of proline residues	14	

Total number of residues	246	

Based on an analysis of 118 structures of resolution of at least 2.0 Angstroms and R-factor no greater than 20%, a good quality model would be expected to have over 90% in the most favoured regions.

7450777_01.ps

12- LysB-Anubis

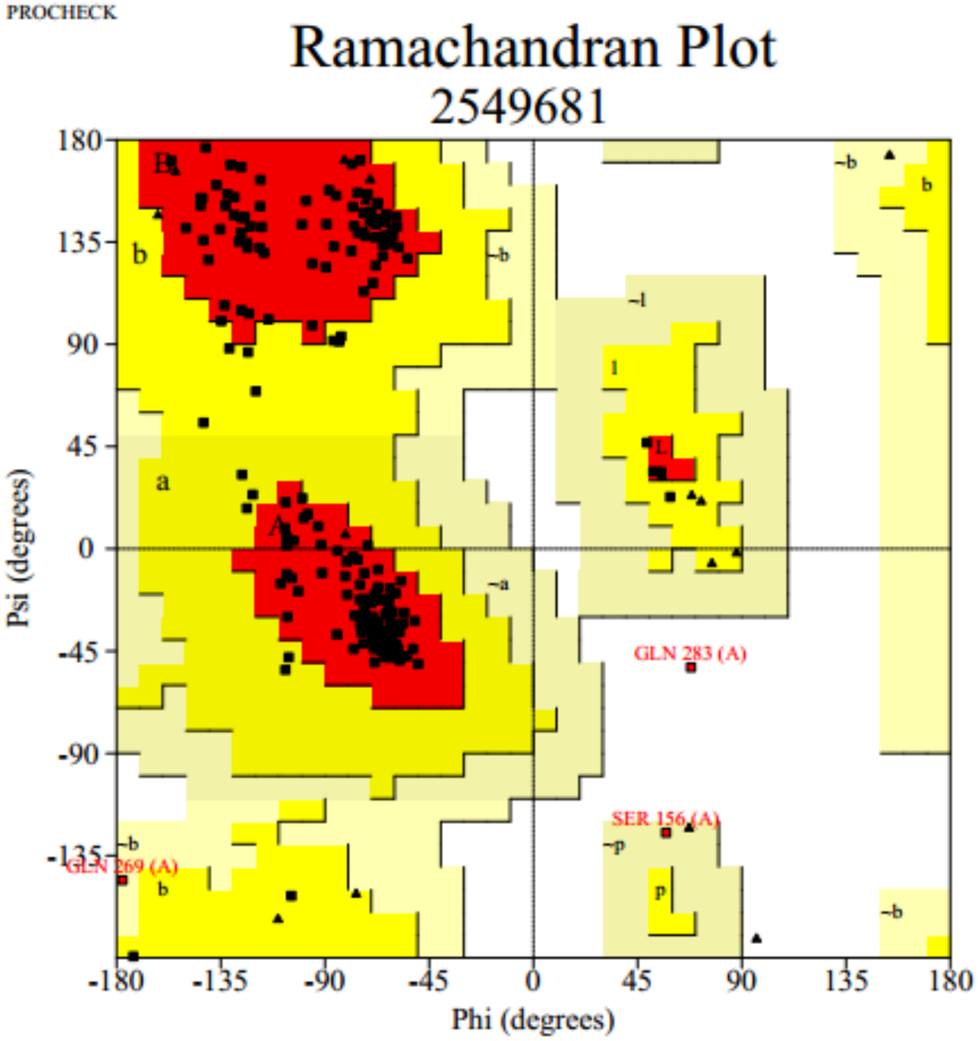

Plot statistics

Residues in most favoured regions [A,B,L]	190	90.5%
Residues in additional allowed regions [a,b,l,p]	17	8.1%
Residues in generously allowed regions [~a,~b,~l,~p]	2	1.0%
Residues in disallowed regions	1	0.5%
	----	------
Number of non-glycine and non-proline residues	210	100.0%
Number of end-residues (excl. Gly and Pro)	2	
Number of glycine residues (shown as triangles)	23	
Number of proline residues	16	

Total number of residues	251	

Based on an analysis of 118 structures of resolution of at least 2.0 Angstroms and R-factor no greater than 20%, a good quality model would be expected to have over 90% in the most favoured regions.

2549681_01.ps

13- LysB-BXZ2

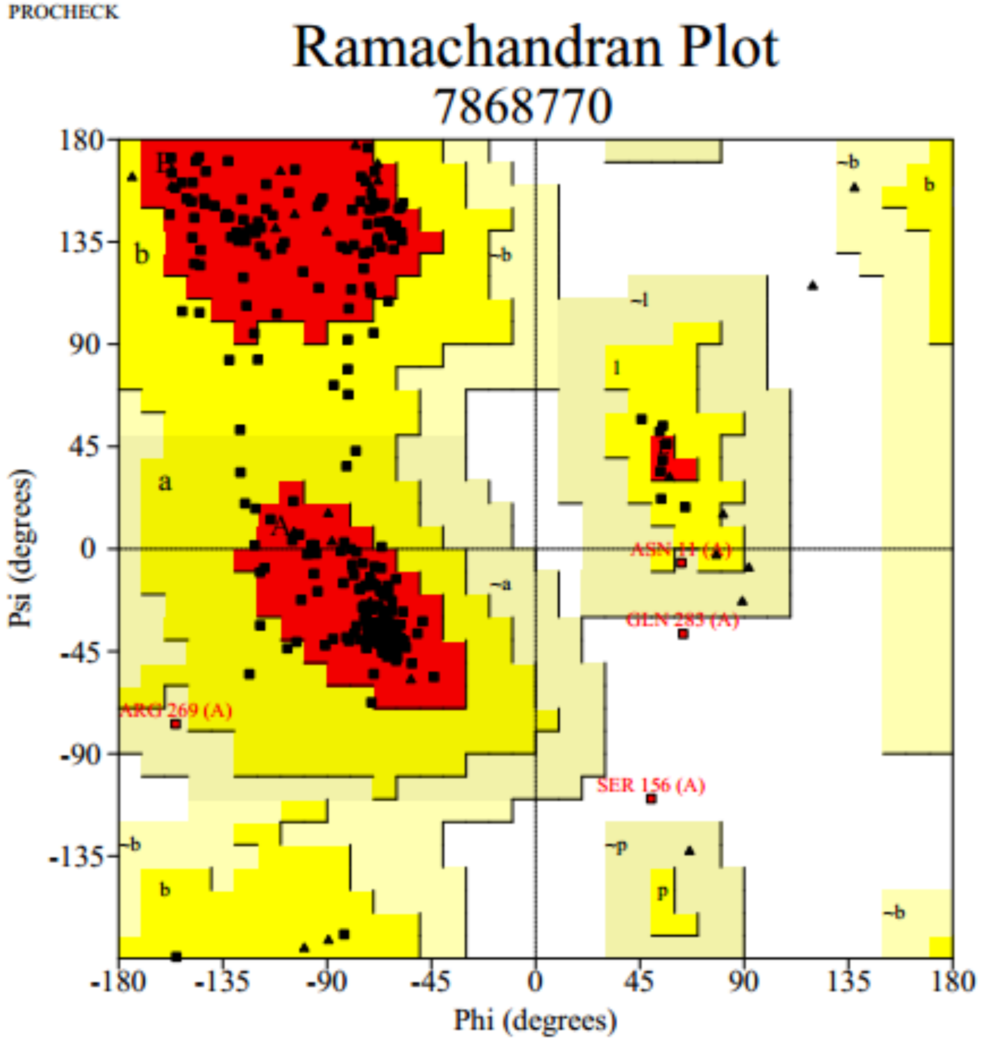

Plot statistics

Residues in most favoured regions [A,B,L]	237	87.8%
Residues in additional allowed regions [a,b,l,p]	29	10.7%
Residues in generously allowed regions [~a,~b,~l,~p]	2	0.7%
Residues in disallowed regions	2	0.7%
	----	------
Number of non-glycine and non-proline residues	270	100.0%
Number of end-residues (excl. Gly and Pro)	2	
Number of glycine residues (shown as triangles)	33	
Number of proline residues	16	

Total number of residues	321	

Based on an analysis of 118 structures of resolution of at least 2.0 Angstroms and R-factor no greater than 20%, a good quality model would be expected to have over 90% in the most favoured regions.

7868770_01.ps

14- LysB-Severus

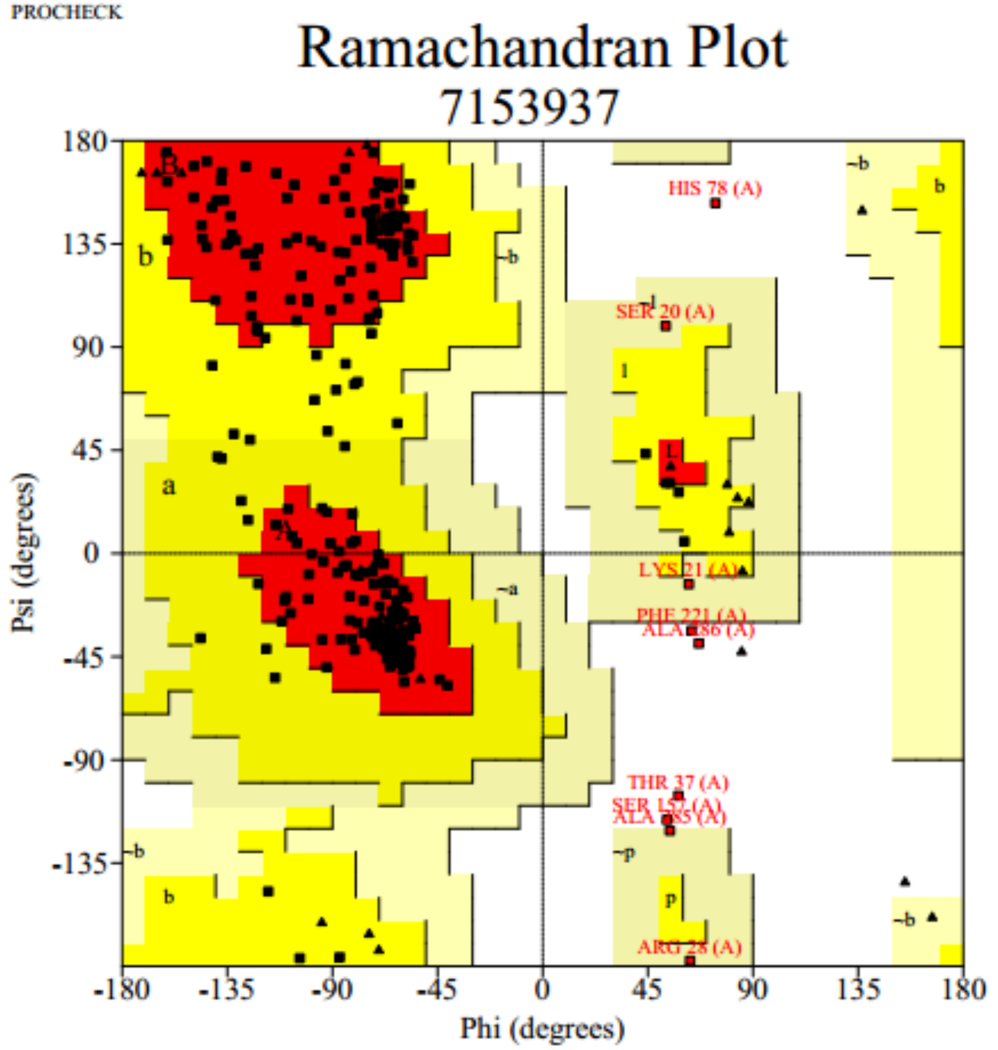

Plot statistics

Residues in most favoured regions [A,B,L]	232	85.9%
Residues in additional allowed regions [a,b,l,p]	29	10.7%
Residues in generously allowed regions [~a,~b,~l,~p]	4	1.5%
Residues in disallowed regions	5	1.9%
	----	------
Number of non-glycine and non-proline residues	270	100.0%
Number of end-residues (excl. Gly and Pro)	2	
Number of glycine residues (shown as triangles)	29	
Number of proline residues	23	

Total number of residues	324	

Based on an analysis of 118 structures of resolution of at least 2.0 Angstroms and R-factor no greater than 20%, a good quality model would be expected to have over 90% in the most favoured regions.

7153937_01.ps

15- LysB-Zakai

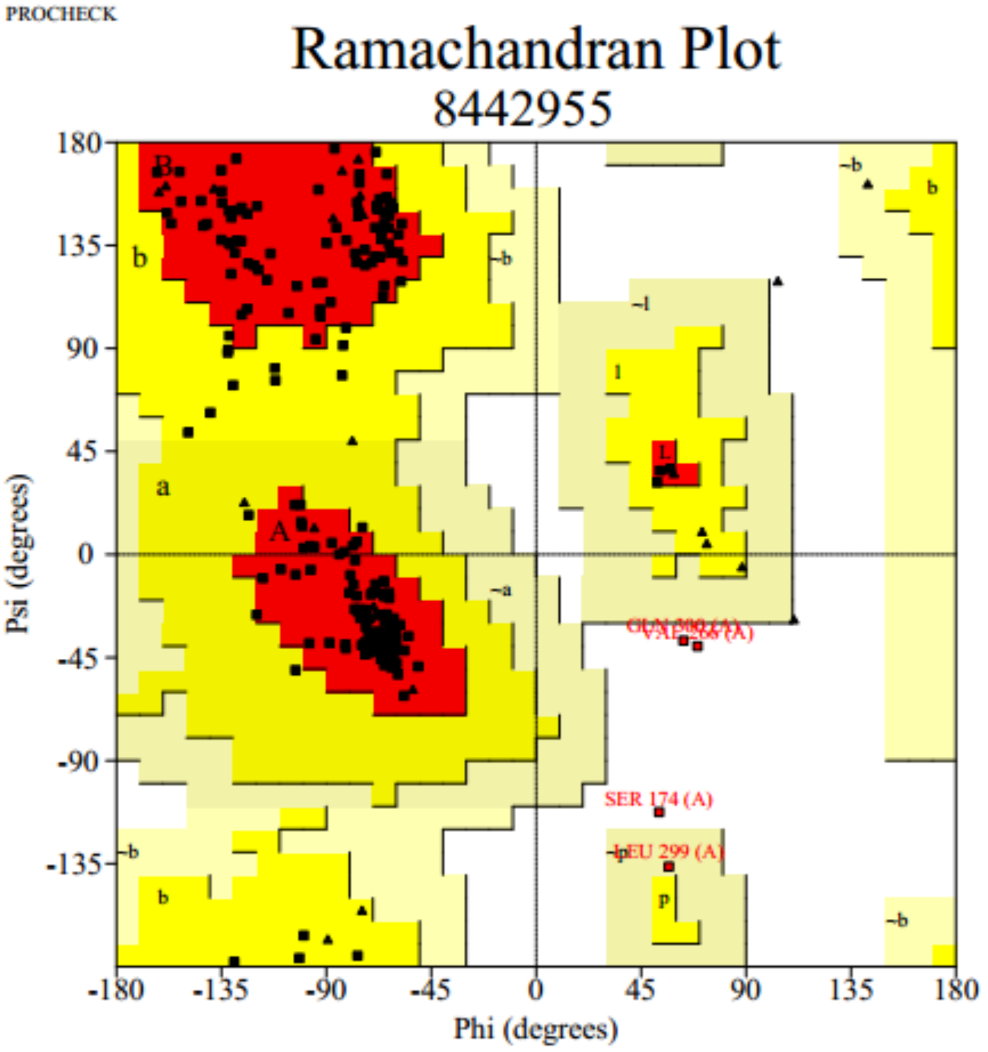

Plot statistics

Residues in most favoured regions [A,B,L]	190	89.2%
Residues in additional allowed regions [a,b,l,p]	19	8.9%
Residues in generously allowed regions [~a,~b,~l,~p]	1	0.5%
Residues in disallowed regions	3	1.4%
	----	------
Number of non-glycine and non-proline residues	213	100.0%
Number of end-residues (excl. Gly and Pro)	2	
Number of glycine residues (shown as triangles)	26	
Number of proline residues	18	

Total number of residues	259	

Based on an analysis of 118 structures of resolution of at least 2.0 Angstroms and R-factor no greater than 20%, a good quality model would be expected to have over 90% in the most favoured regions.

8442955_01.ps

16- LysB-Wildcat

PROCHECK

Ramachandran Plot

9101217

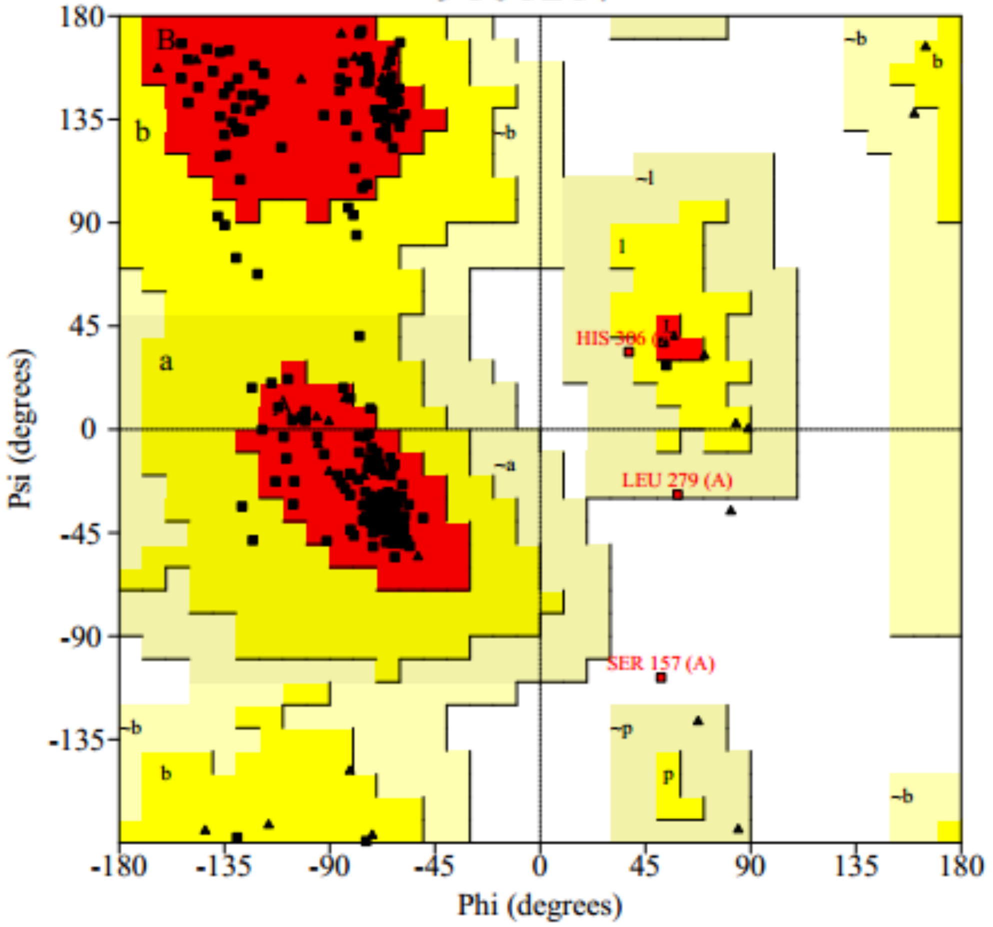

Plot statistics

Residues in most favoured regions [A,B,L]	191	91.4%
Residues in additional allowed regions [a,b,l,p]	15	7.2%
Residues in generously allowed regions [~a,~b,~l,~p]	2	1.0%
Residues in disallowed regions	1	0.5%
	----	------
Number of non-glycine and non-proline residues	209	100.0%
Number of end-residues (excl. Gly and Pro)	2	
Number of glycine residues (shown as triangles)	31	
Number of proline residues	19	

Total number of residues	261	

Based on an analysis of 118 structures of resolution of at least 2.0 Angstroms and R-factor no greater than 20%, a good quality model would be expected to have over 90% in the most favoured regions.

9101217_01.ps

17- LysB-Ardmore

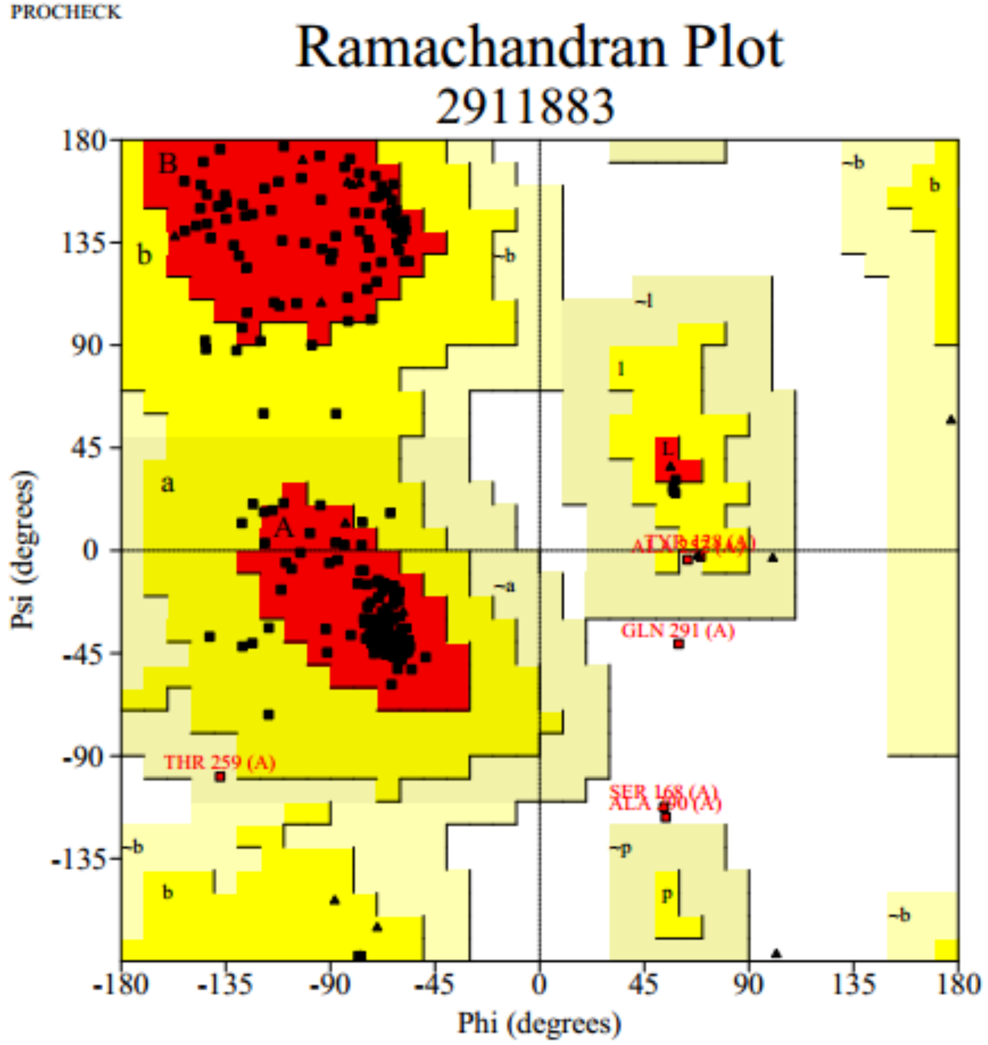

Plot statistics

Residues in most favoured regions [A,B,L]	182	87.9%
Residues in additional allowed regions [a,b,l,p]	19	9.2%
Residues in generously allowed regions [~a,~b,~l,~p]	3	1.4%
Residues in disallowed regions	3	1.4%
	----	------
Number of non-glycine and non-proline residues	207	100.0%
Number of end-residues (excl. Gly and Pro)	1	
Number of glycine residues (shown as triangles)	22	
Number of proline residues	21	

Total number of residues	251	

Based on an analysis of 118 structures of resolution of at least 2.0 Angstroms and R-factor no greater than 20%, a good quality model would be expected to have over 90% in the most favoured regions.

2911883_01.ps

18- LysB-Ms6

PROCHECK

Ramachandran Plot

5081248

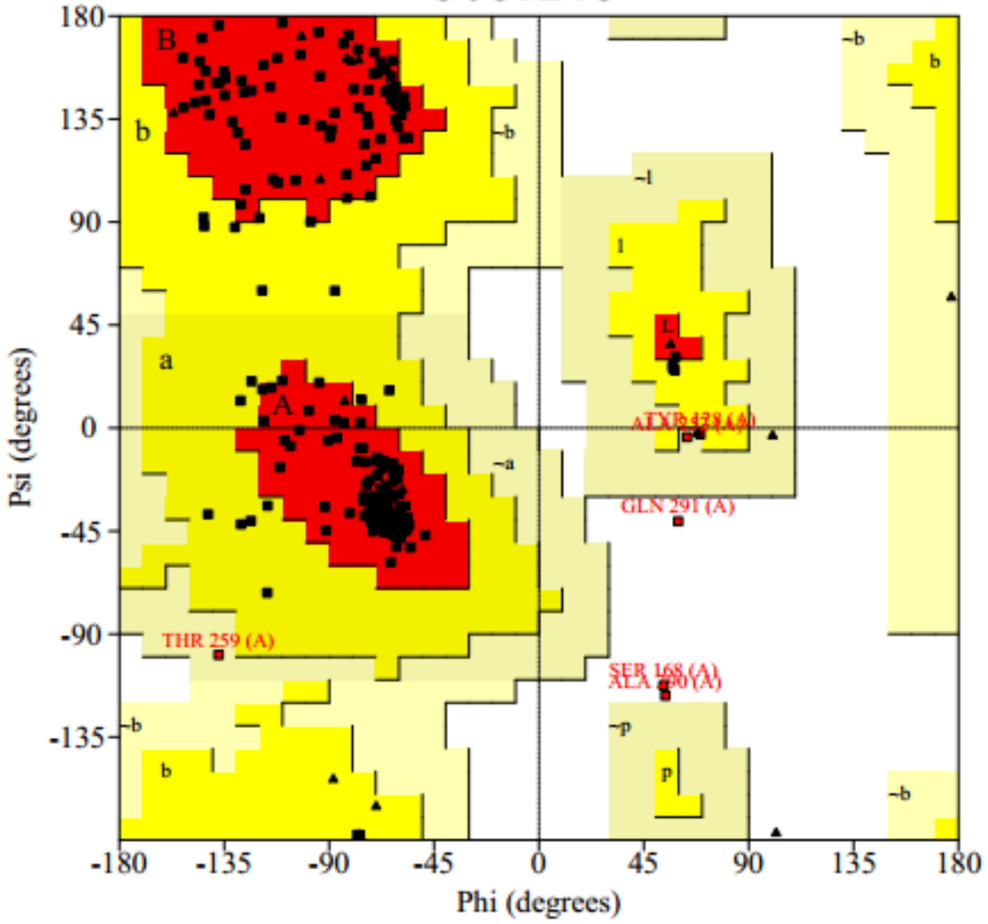

Plot statistics

Residues in most favoured regions [A,B,L]	182	87.9%
Residues in additional allowed regions [a,b,l,p]	19	9.2%
Residues in generously allowed regions [~a,~b,~l,~p]	3	1.4%
Residues in disallowed regions	3	1.4%
	----	------
Number of non-glycine and non-proline residues	207	100.0%
Number of end-residues (excl. Gly and Pro)	1	
Number of glycine residues (shown as triangles)	22	
Number of proline residues	21	

Total number of residues	251	

Based on an analysis of 118 structures of resolution of at least 2.0 Angstroms and R-factor no greater than 20%, a good quality model would be expected to have over 90% in the most favoured regions.

5081248_01.ps

19- LysB-Hades

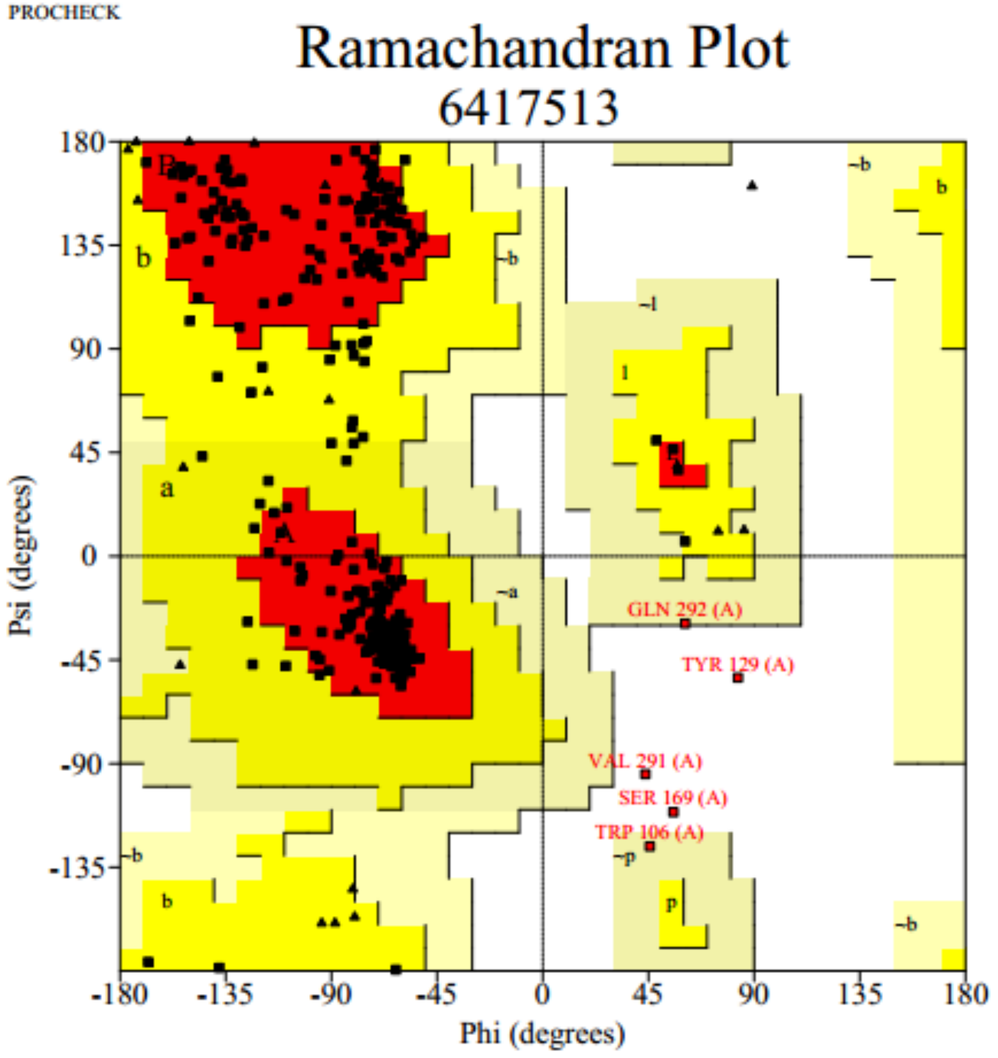

Plot statistics

Residues in most favoured regions [A,B,L]	239	86.9%
Residues in additional allowed regions [a,b,l,p]	31	11.3%
Residues in generously allowed regions [~a,~b,~l,~p]	2	0.7%
Residues in disallowed regions	3	1.1%
	----	------
Number of non-glycine and non-proline residues	275	100.0%
Number of end-residues (excl. Gly and Pro)	2	
Number of glycine residues (shown as triangles)	30	
Number of proline residues	26	

Total number of residues	333	

Based on an analysis of 118 structures of resolution of at least 2.0 Angstroms and R-factor no greater than 20%, a good quality model would be expected to have over 90% in the most favoured regions.

6417513_01.ps

20- LysB-Saal

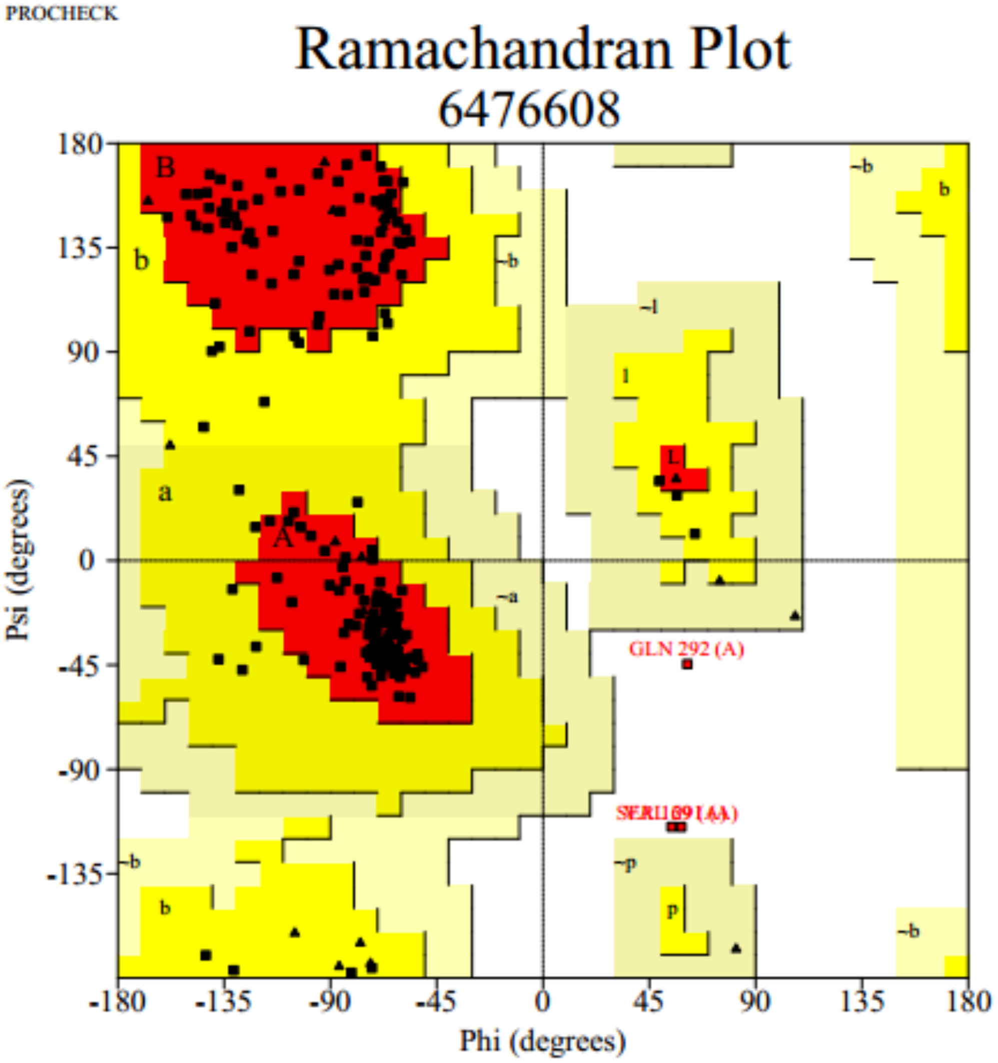

Plot statistics

Residues in most favoured regions [A,B,L]	184	88.9%
Residues in additional allowed regions [a,b,l,p]	20	9.7%
Residues in generously allowed regions [~a,~b,~l,~p]	0	0.0%
Residues in disallowed regions	3	1.4%
	----	------
Number of non-glycine and non-proline residues	207	100.0%
Number of end-residues (excl. Gly and Pro)	1	
Number of glycine residues (shown as triangles)	21	
Number of proline residues	22	

Total number of residues	251	

Based on an analysis of 118 structures of resolution of at least 2.0 Angstroms and R-factor no greater than 20%, a good quality model would be expected to have over 90% in the most favoured regions.

6476608_01.ps

21- LysB-Pumpkin

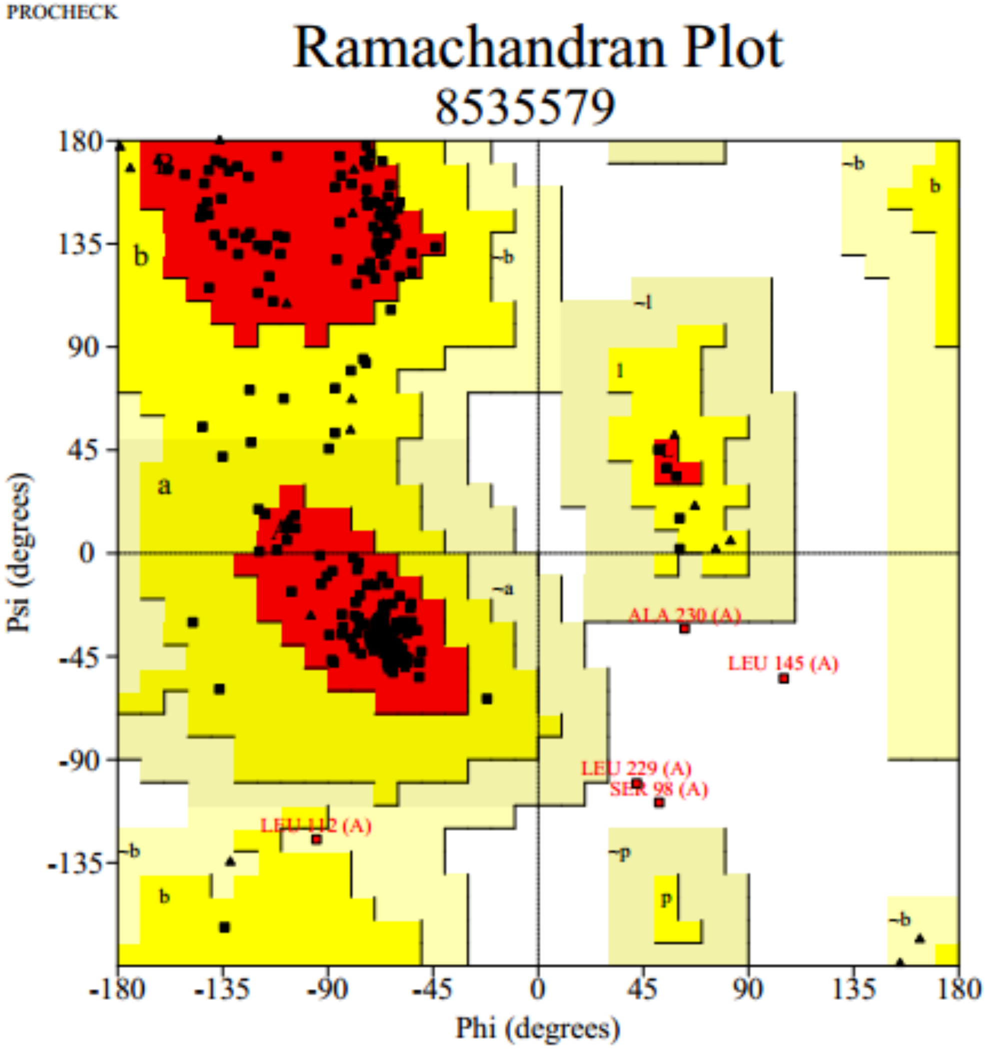

Plot statistics

Residues in most favoured regions [A,B,L]	205	89.5%
Residues in additional allowed regions [a,b,l,p]	19	8.3%
Residues in generously allowed regions [~a,~b,~l,~p]	1	0.4%
Residues in disallowed regions	4	1.7%
	----	------
Number of non-glycine and non-proline residues	229	100.0%
Number of end-residues (excl. Gly and Pro)	2	
Number of glycine residues (shown as triangles)	31	
Number of proline residues	15	

Total number of residues	277	

Based on an analysis of 118 structures of resolution of at least 2.0 Angstroms and R-factor no greater than 20%, a good quality model would be expected to have over 90% in the most favoured regions.

8535579_01.ps

22- LysB-Graduation

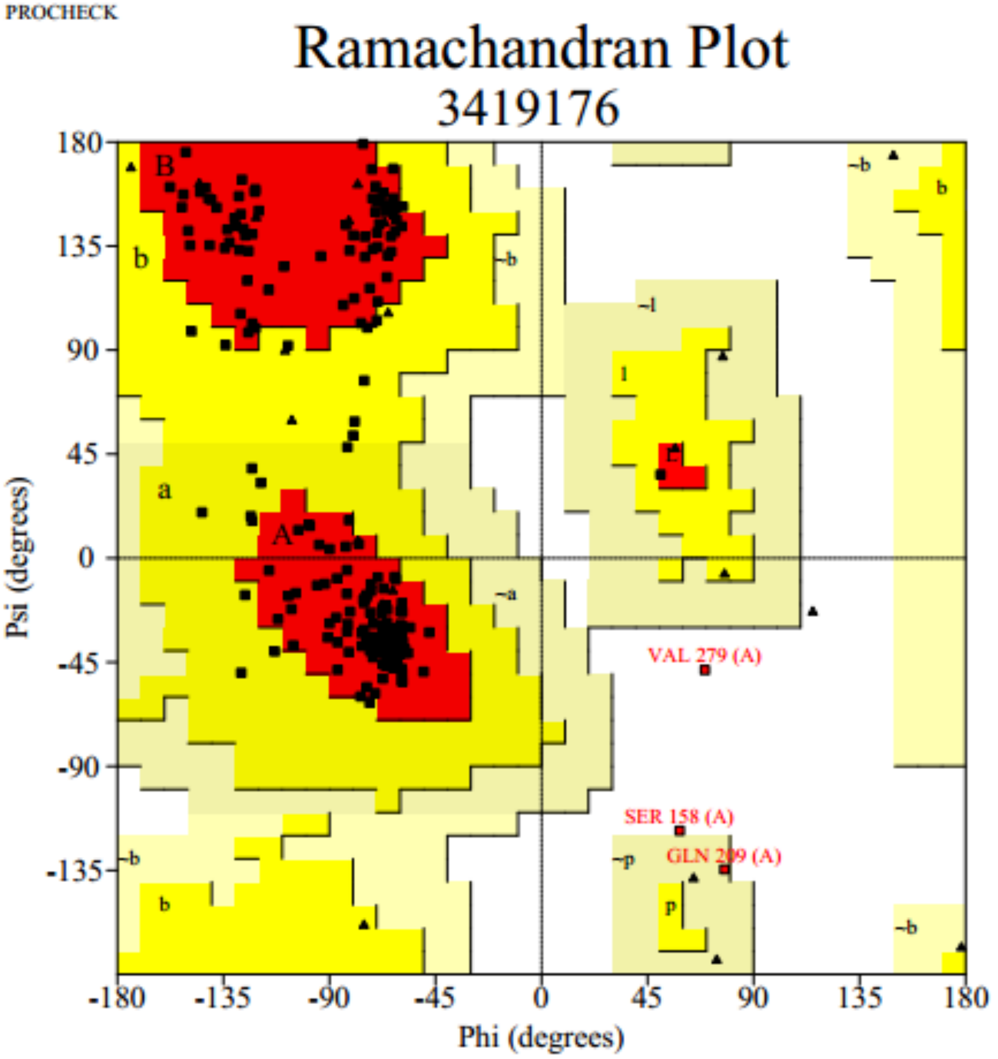

Plot statistics

Residues in most favoured regions [A,B,L]	198	90.8%
Residues in additional allowed regions [a,b,l,p]	17	7.8%
Residues in generously allowed regions [~a,~b,~l,~p]	1	0.5%
Residues in disallowed regions	2	0.9%
	----	------
Number of non-glycine and non-proline residues	218	100.0%
Number of end-residues (excl. Gly and Pro)	2	
Number of glycine residues (shown as triangles)	27	
Number of proline residues	21	

Total number of residues	268	

Based on an analysis of 118 structures of resolution of at least 2.0 Angstroms and R-factor no greater than 20%, a good quality model would be expected to have over 90% in the most favoured regions.

3419176_01.ps

23- LysB-Mrmagoo

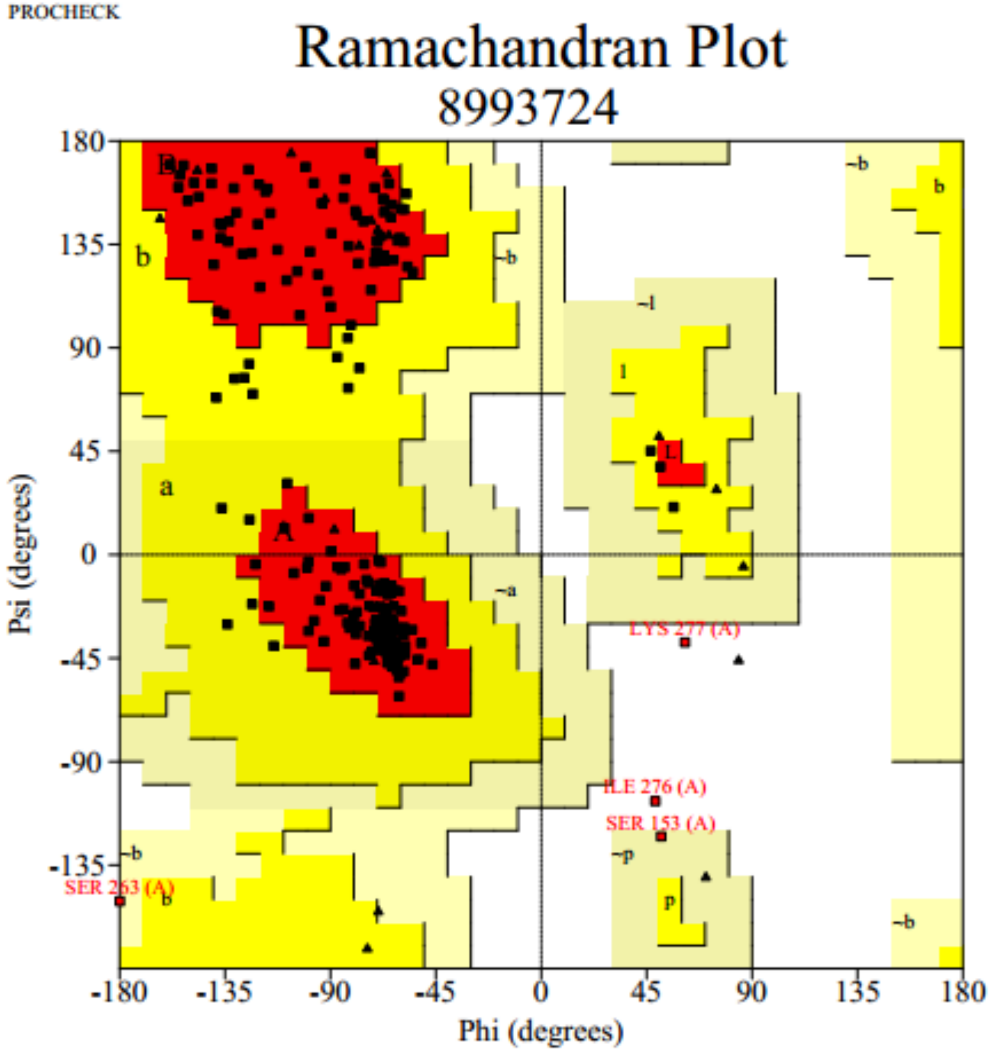

Plot statistics

Residues in most favoured regions [A,B,L]	194	90.2%
Residues in additional allowed regions [a,b,l,p]	17	7.9%
Residues in generously allowed regions [~a,~b,~l,~p]	2	0.9%
Residues in disallowed regions	2	0.9%
	----	------
Number of non-glycine and non-proline residues	215	100.0%
Number of end-residues (excl. Gly and Pro)	2	
Number of glycine residues (shown as triangles)	26	
Number of proline residues	17	

Total number of residues	260	

Based on an analysis of 118 structures of resolution of at least 2.0 Angstroms and R-factor no greater than 20%, a good quality model would be expected to have over 90% in the most favoured regions.

8993724_01.ps

24- LysB-Dylan

PROCHECK

Ramachandran Plot

1234491

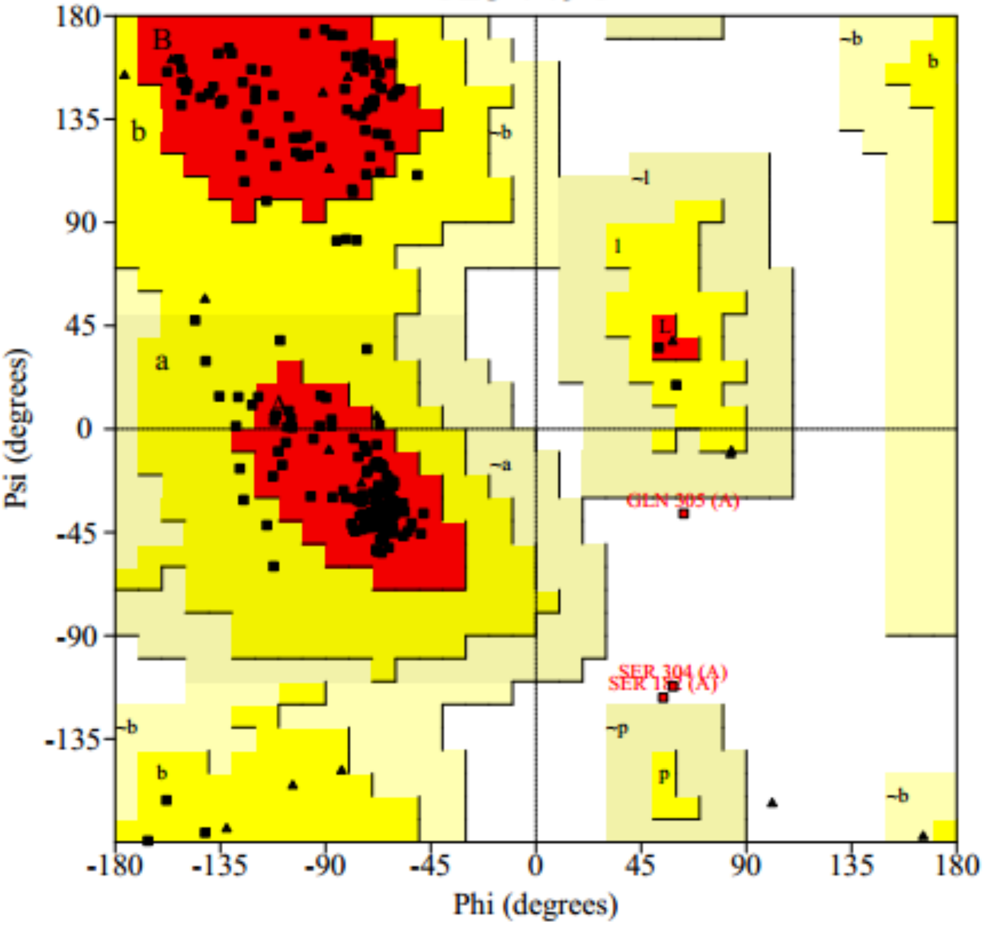

Plot statistics

Residues in most favoured regions [A,B,L]	183	88.0%
Residues in additional allowed regions [a,b,l,p]	22	10.6%
Residues in generously allowed regions [~a,~b,~l,~p]	0	0.0%
Residues in disallowed regions	3	1.4%
	----	------
Number of non-glycine and non-proline residues	208	100.0%
Number of end-residues (excl. Gly and Pro)	2	
Number of glycine residues (shown as triangles)	27	
Number of proline residues	20	

Total number of residues	257	

Based on an analysis of 118 structures of resolution of at least 2.0 Angstroms and R-factor no greater than 20%, a good quality model would be expected to have over 90% in the most favoured regions.

1234491_01.ps

25- LysB-DS6A

PROCHECK

Ramachandran Plot

7718541

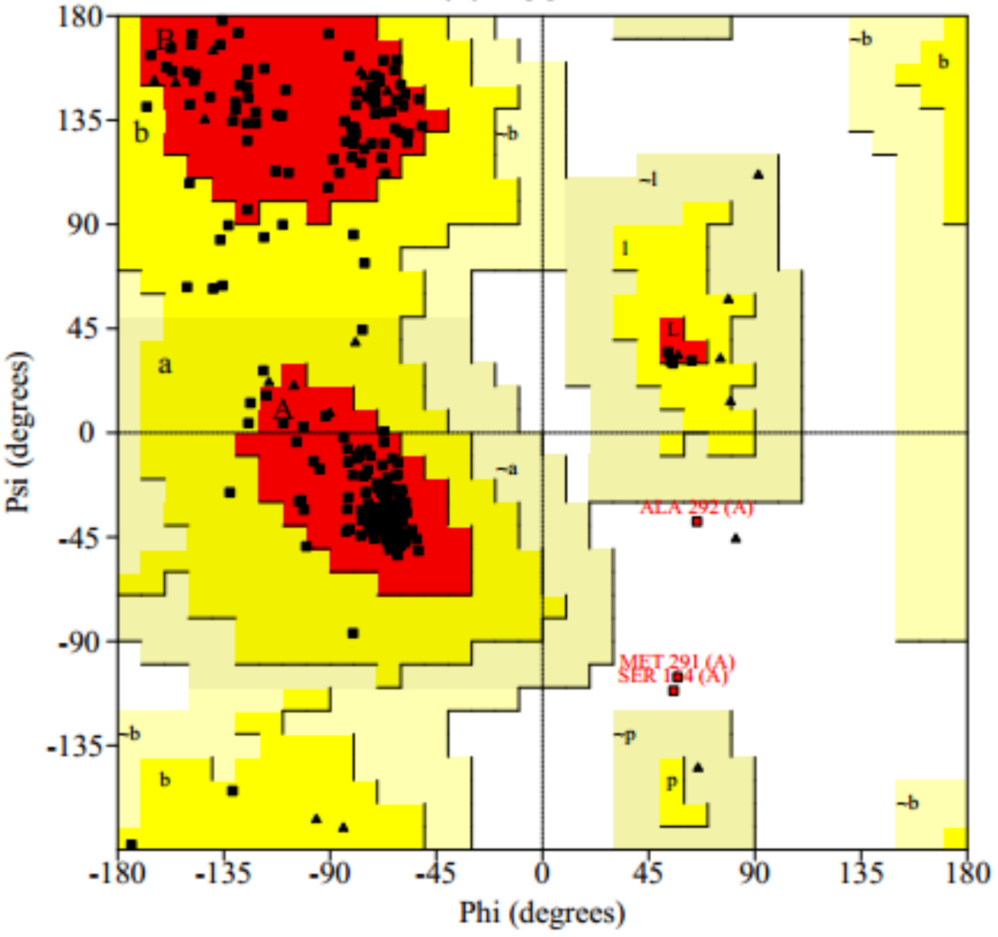

Plot statistics

Residues in most favoured regions [A,B,L]	195	89.0%
Residues in additional allowed regions [a,b,l,p]	21	9.6%
Residues in generously allowed regions [~a,~b,~l,~p]	0	0.0%
Residues in disallowed regions	3	1.4%
	----	------
Number of non-glycine and non-proline residues	219	100.0%
Number of end-residues (excl. Gly and Pro)	2	
Number of glycine residues (shown as triangles)	28	
Number of proline residues	22	

Total number of residues	271	

Based on an analysis of 118 structures of resolution of at least 2.0 Angstroms and R-factor no greater than 20%, a good quality model would be expected to have over 90% in the most favoured regions.

7718541_01.ps

26- LysB-Palestino

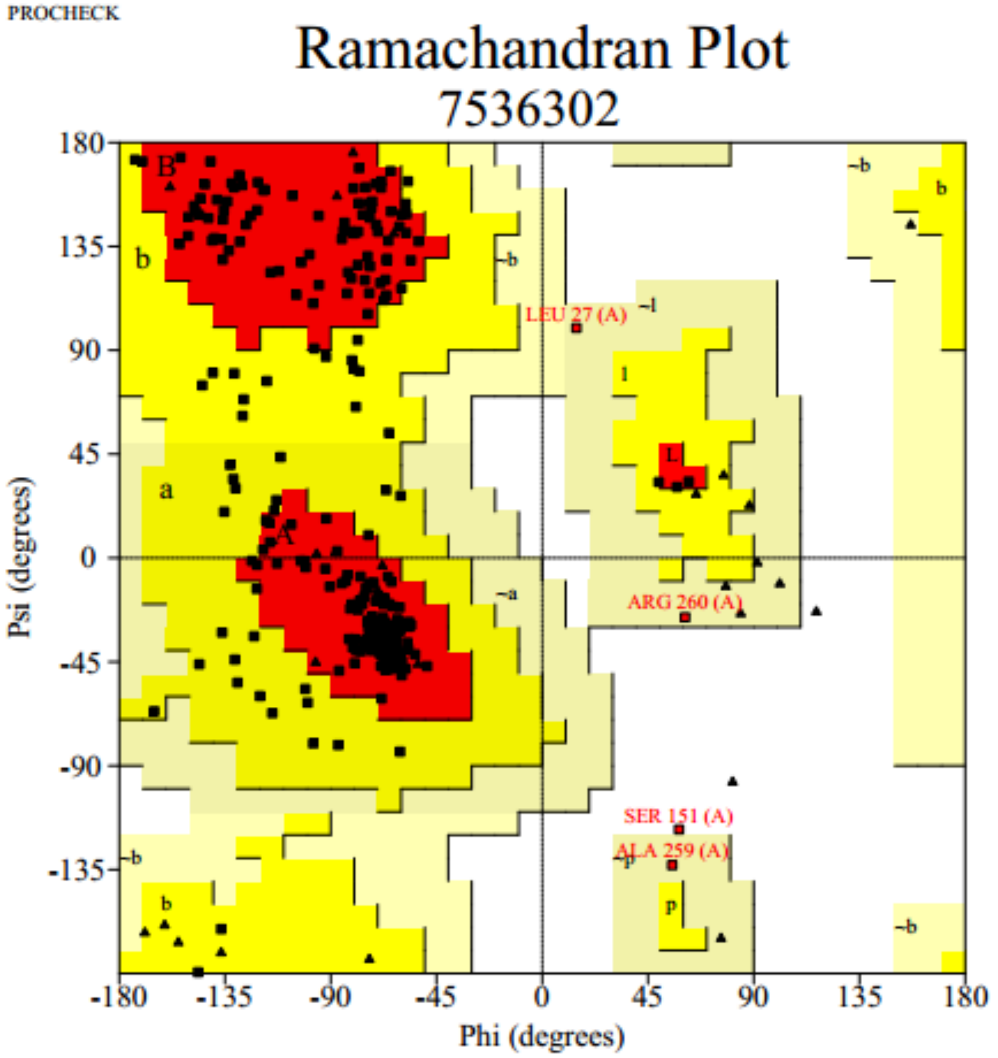

Plot statistics

Residues in most favoured regions [A,B,L]	217	82.8%
Residues in additional allowed regions [a,b,l,p]	41	15.6%
Residues in generously allowed regions [~a,~b,~l,~p]	3	1.1%
Residues in disallowed regions	1	0.4%
	----	------
Number of non-glycine and non-proline residues	262	100.0%
Number of end-residues (excl. Gly and Pro)	2	
Number of glycine residues (shown as triangles)	38	
Number of proline residues	20	

Total number of residues	322	

Based on an analysis of 118 structures of resolution of at least 2.0 Angstroms and R-factor no greater than 20%, a good quality model would be expected to have over 90% in the most favoured regions.

7536302_01.ps

27- LysB-Obama12

PROCHECK

Ramachandran Plot

2887118

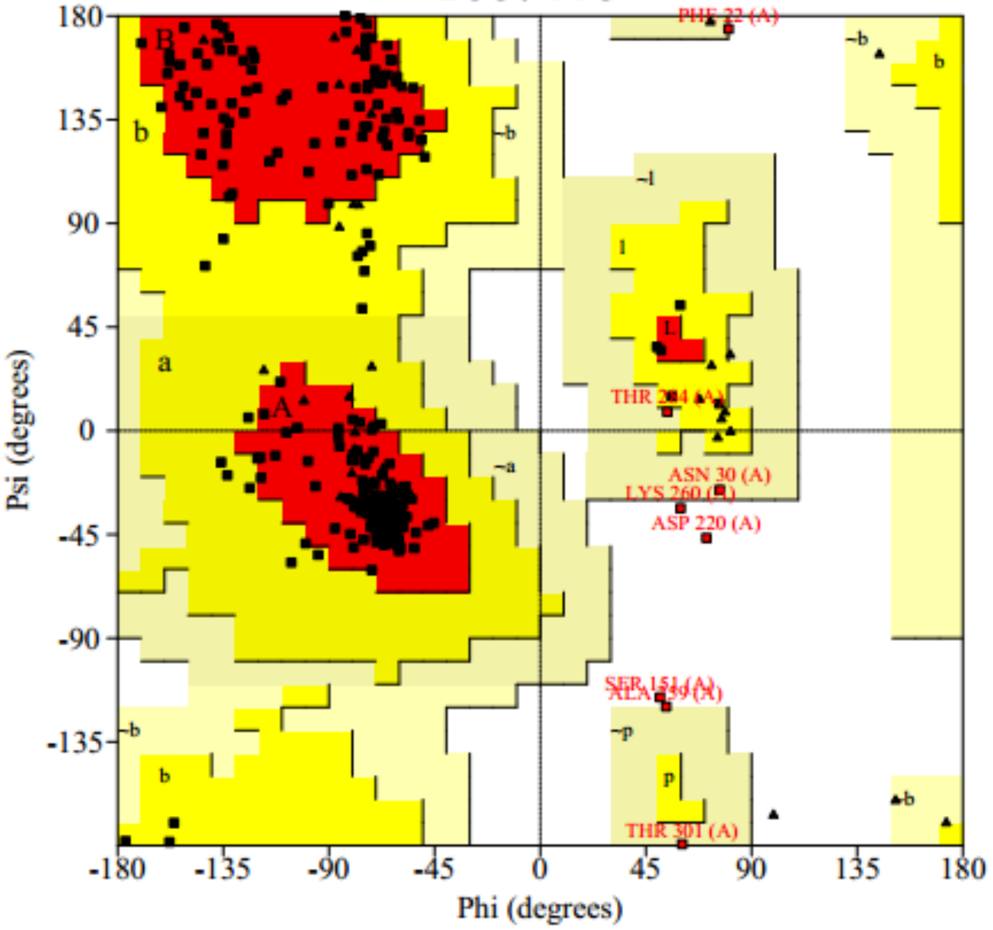

Plot statistics

Residues in most favoured regions [A,B,L]	231	87.2%
Residues in additional allowed regions [a,b,l,p]	26	9.8%
Residues in generously allowed regions [~a,~b,~l,~p]	3	1.1%
Residues in disallowed regions	5	1.9%
	----	------
Number of non-glycine and non-proline residues	265	100.0%
Number of end-residues (excl. Gly and Pro)	2	
Number of glycine residues (shown as triangles)	39	
Number of proline residues	20	

Total number of residues	326	

Based on an analysis of 118 structures of resolution of at least 2.0 Angstroms and R-factor no greater than 20%, a good quality model would be expected to have over 90% in the most favoured regions.

28- LysB-Omega

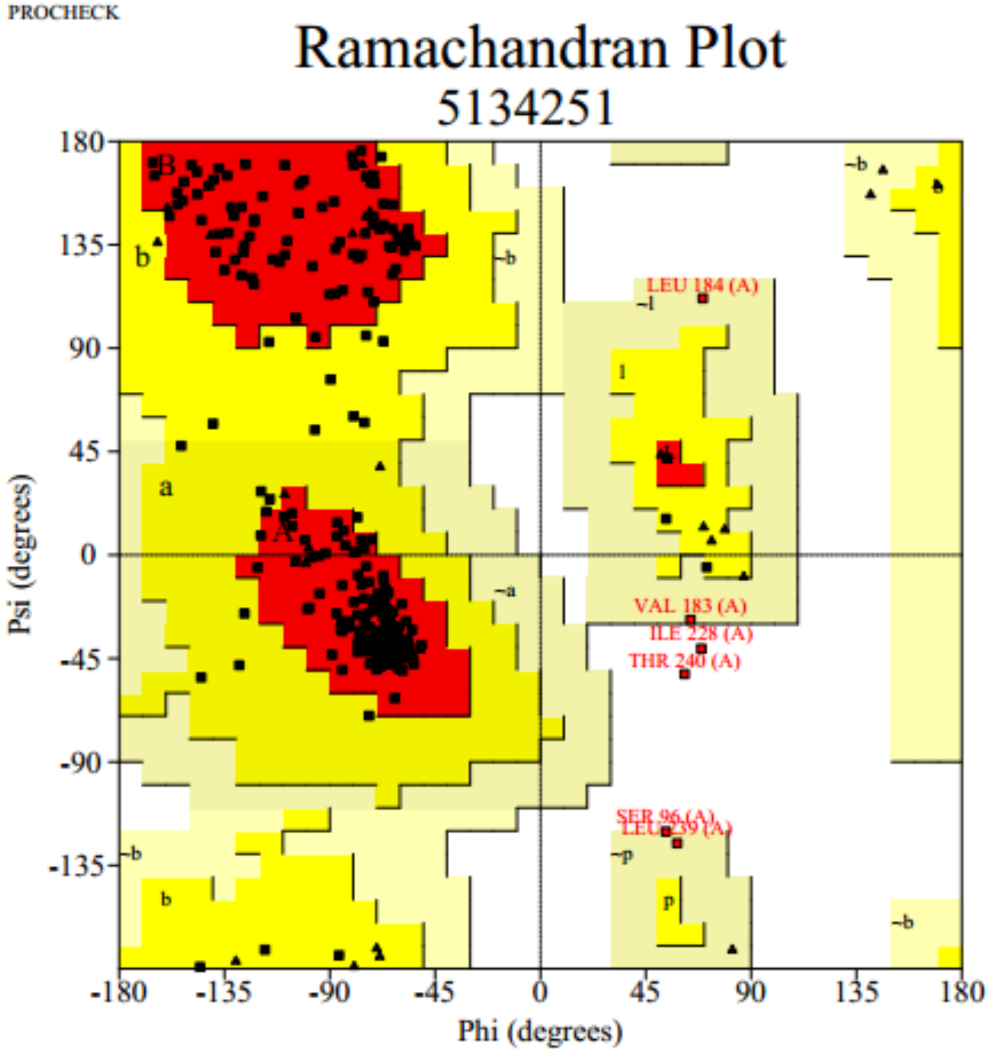

Plot statistics

Residues in most favoured regions [A,B,L]	210	89.0%
Residues in additional allowed regions [a,b,l,p]	20	8.5%
Residues in generously allowed regions [~a,~b,~l,~p]	4	1.7%
Residues in disallowed regions	2	0.8%
	----	------
Number of non-glycine and non-proline residues	236	100.0%
Number of end-residues (excl. Gly and Pro)	2	
Number of glycine residues (shown as triangles)	30	
Number of proline residues	22	

Total number of residues	290	

Based on an analysis of 118 structures of resolution of at least 2.0 Angstroms and R-factor no greater than 20%, a good quality model would be expected to have over 90% in the most favoured regions.

5134251_01.ps

29- LysB-Larva

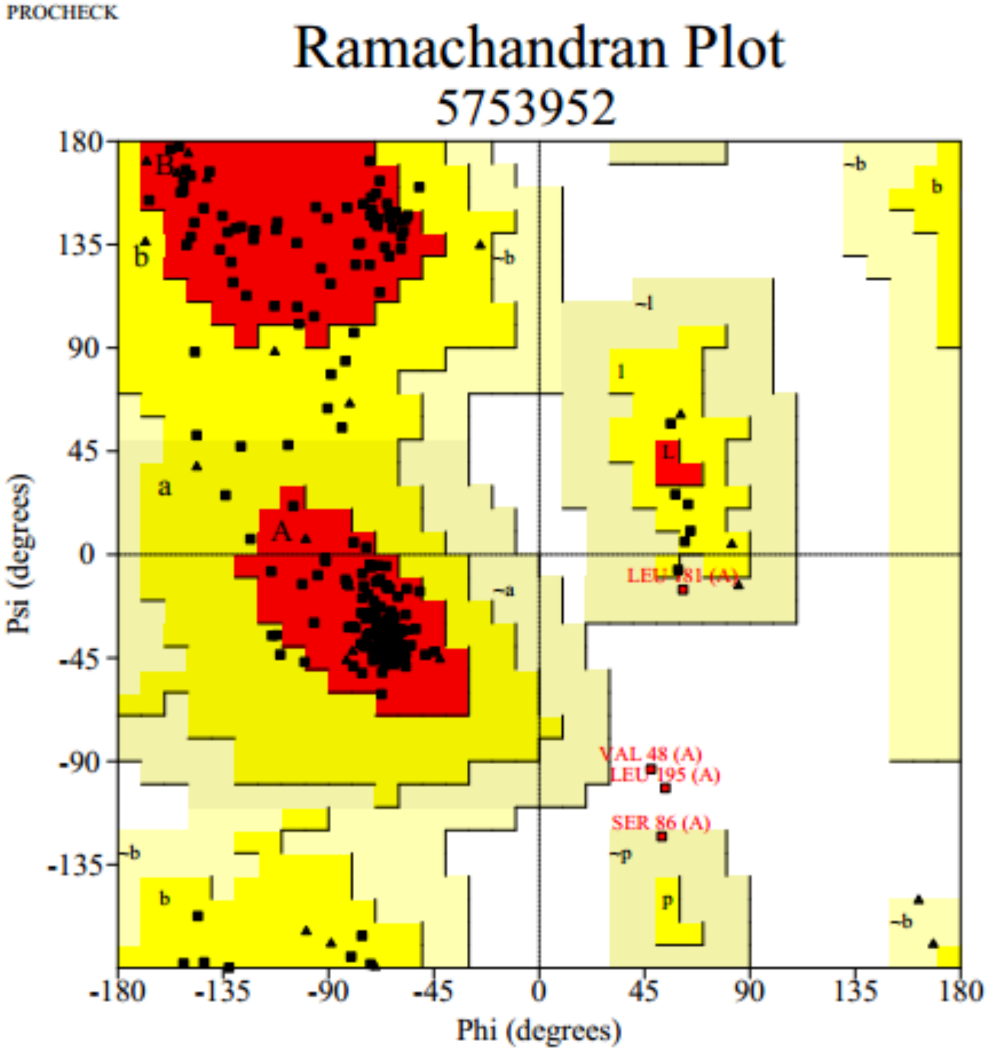

Plot statistics

Residues in most favoured regions [A,B,L]	173	84.4%
Residues in additional allowed regions [a,b,l,p]	28	13.7%
Residues in generously allowed regions [~a,~b,~l,~p]	2	1.0%
Residues in disallowed regions	2	1.0%
	----	------
Number of non-glycine and non-proline residues	205	100.0%
Number of end-residues (excl. Gly and Pro)	2	
Number of glycine residues (shown as triangles)	29	
Number of proline residues	17	

Total number of residues	253	

Based on an analysis of 118 structures of resolution of at least 2.0 Angstroms and R-factor no greater than 20%, a good quality model would be expected to have over 90% in the most favoured regions.

5753952_01.ps

30- LysB-Enkosi

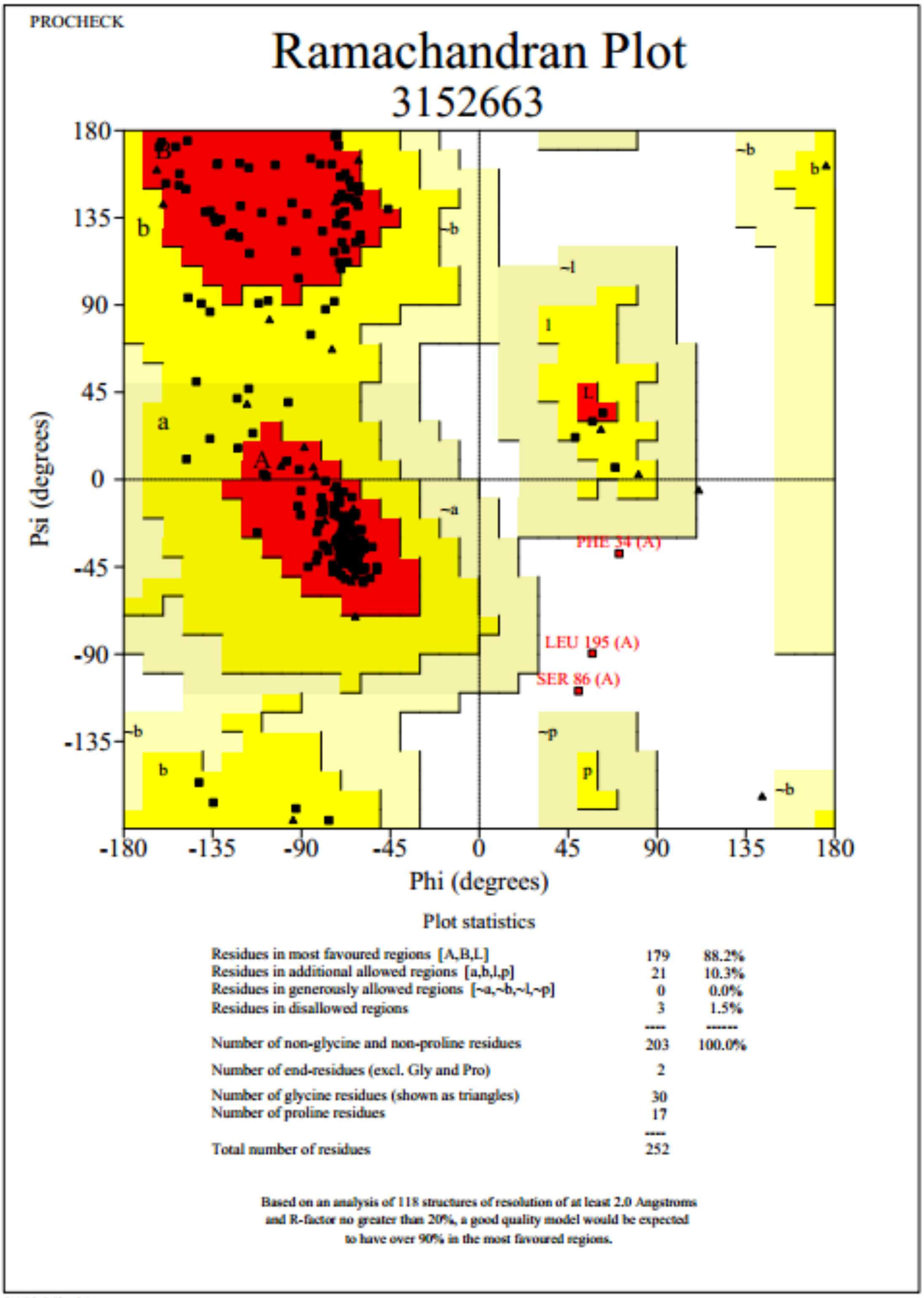

3152663_01.ps

Figure S6: Ramachandran plots of the generated 30 LysB homology models.
