# Supplementary material for: Comparative Structural Analysis of Different Mycobacteriophage-Derived Mycolylarabinogalactan Esterases (Lysin B)
Source: Biomolecules. 2019 Dec 27;10(1):45. doi: 10.3390/biom10010045 (PMC7022511; doi:10.3390/biom10010045)
Supplement: Supplementary file 1 [file biomolecules-10-00045-s001.zip › Supplementary/Supp. fig. 7.pdf]

a

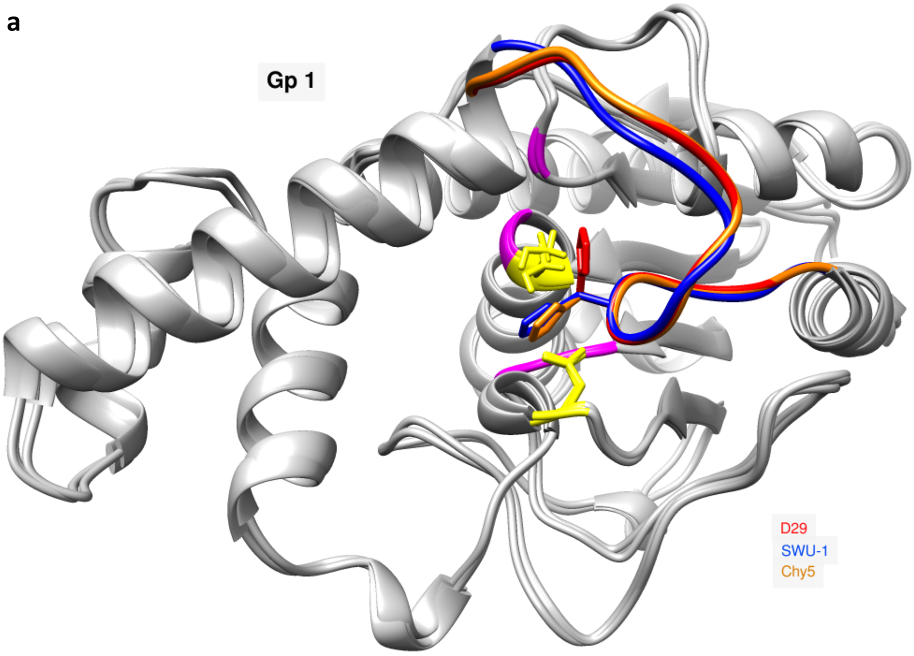

b

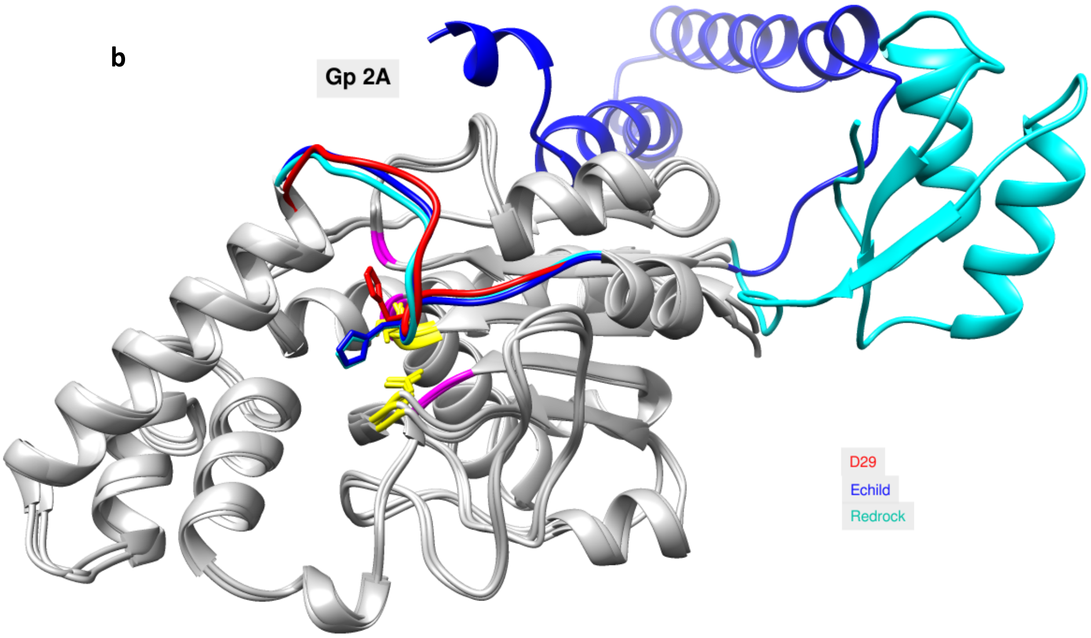

c

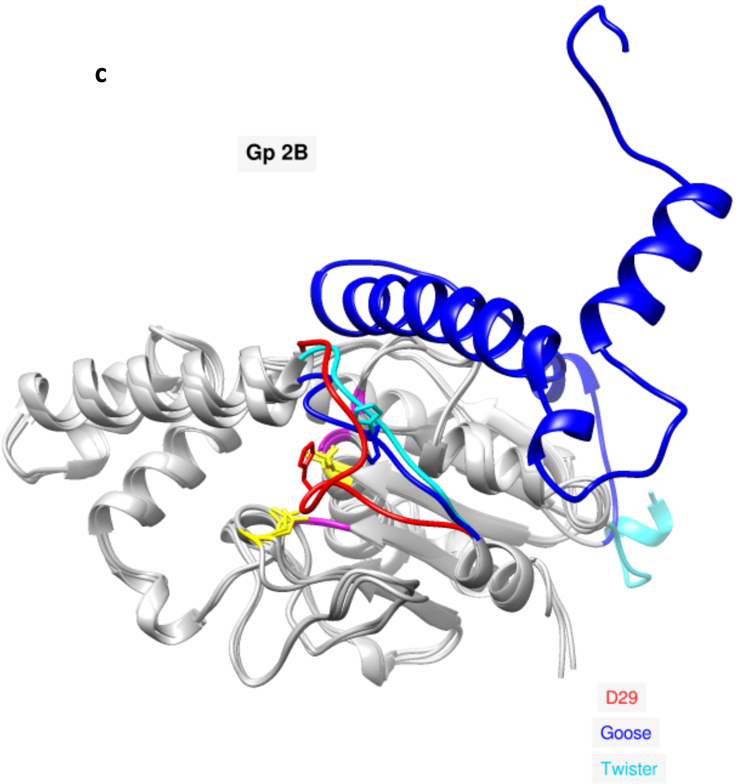

d

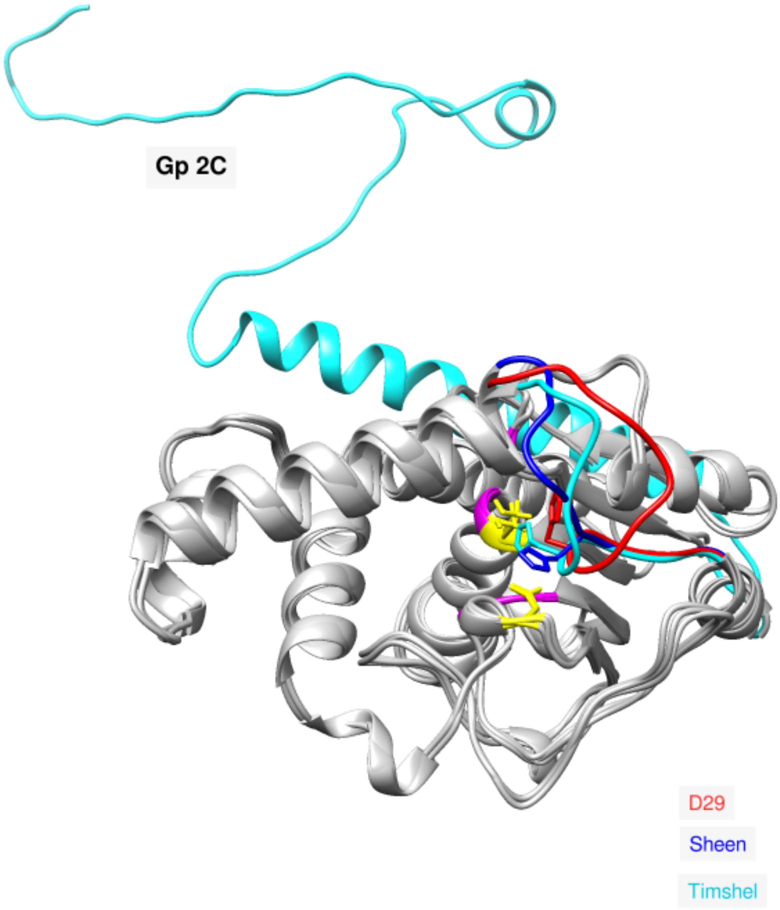

e

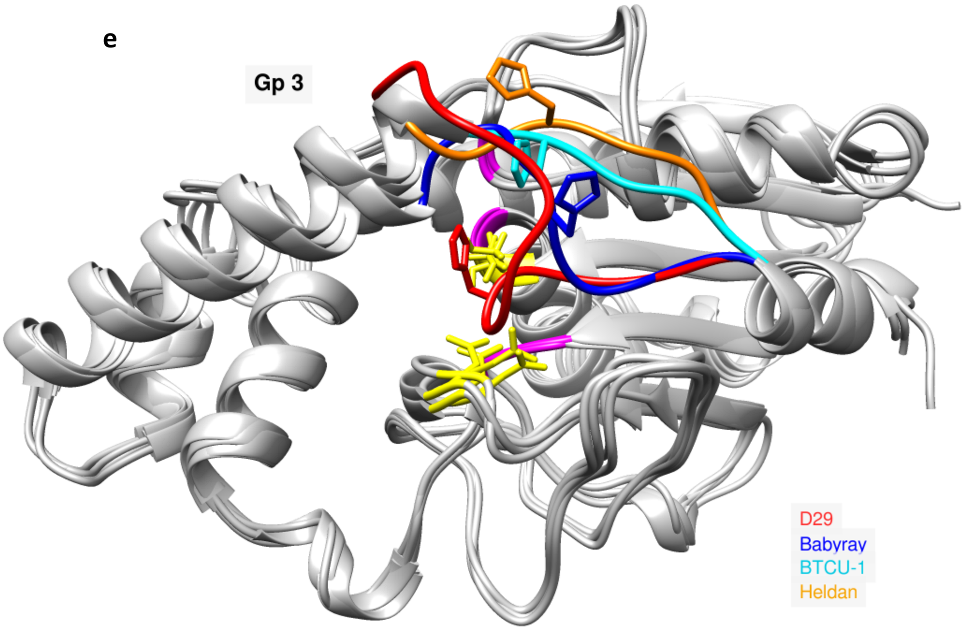

f

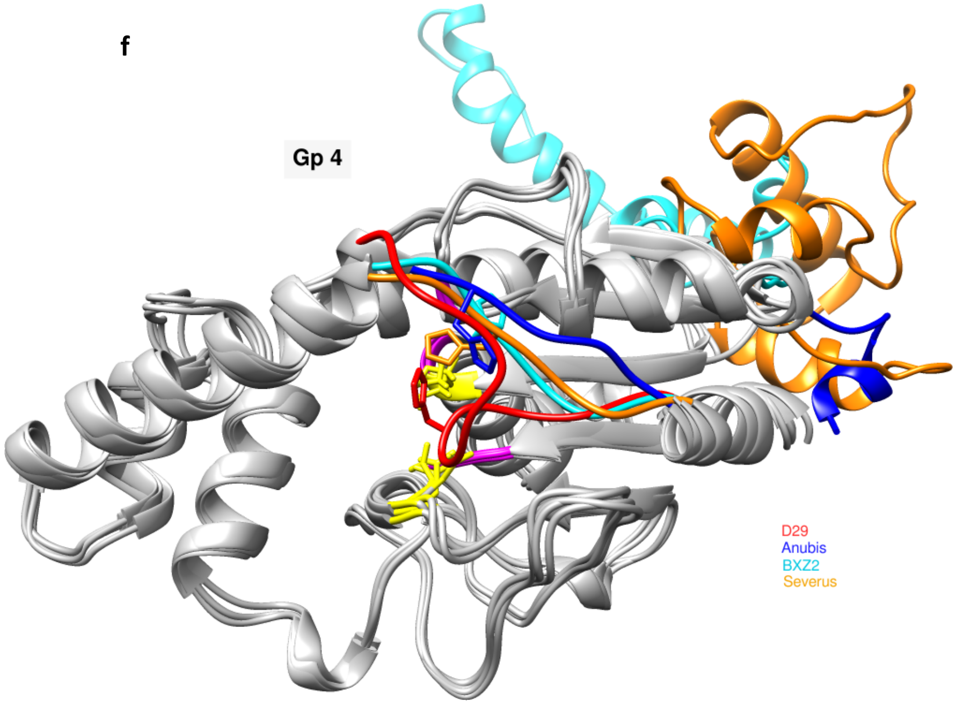

g

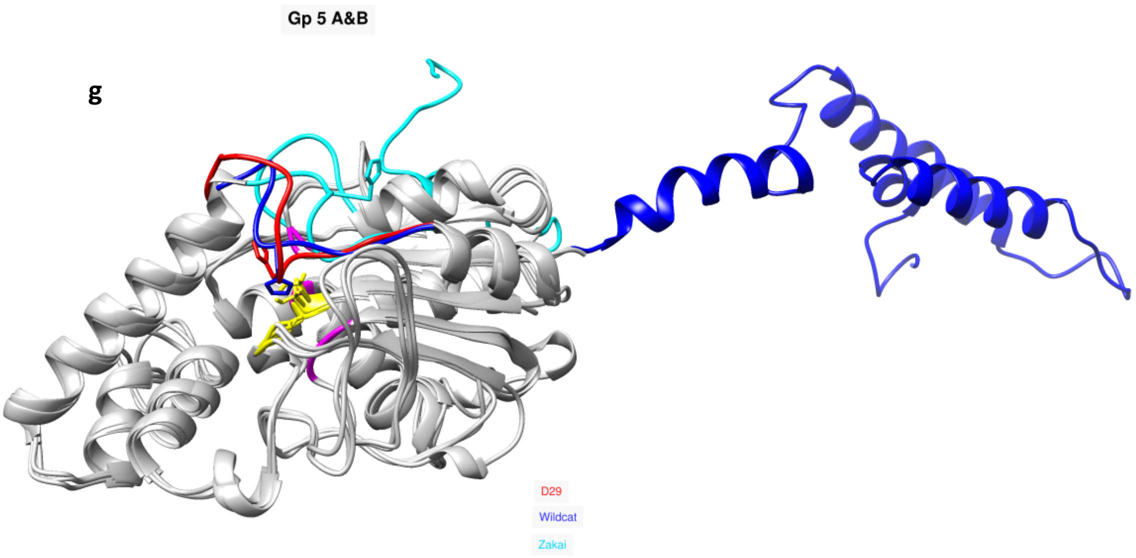

h

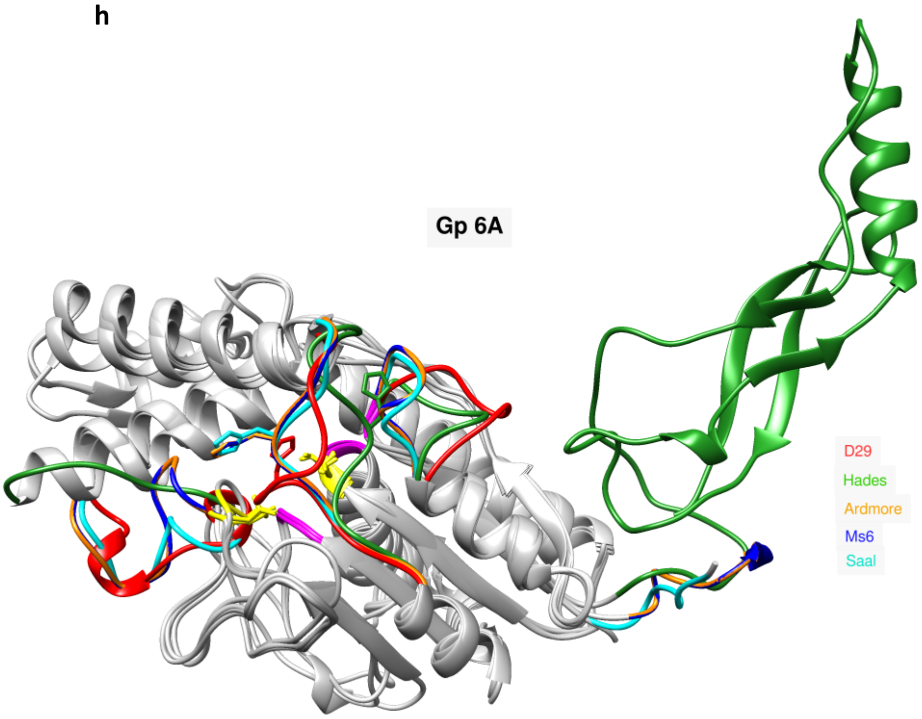

i

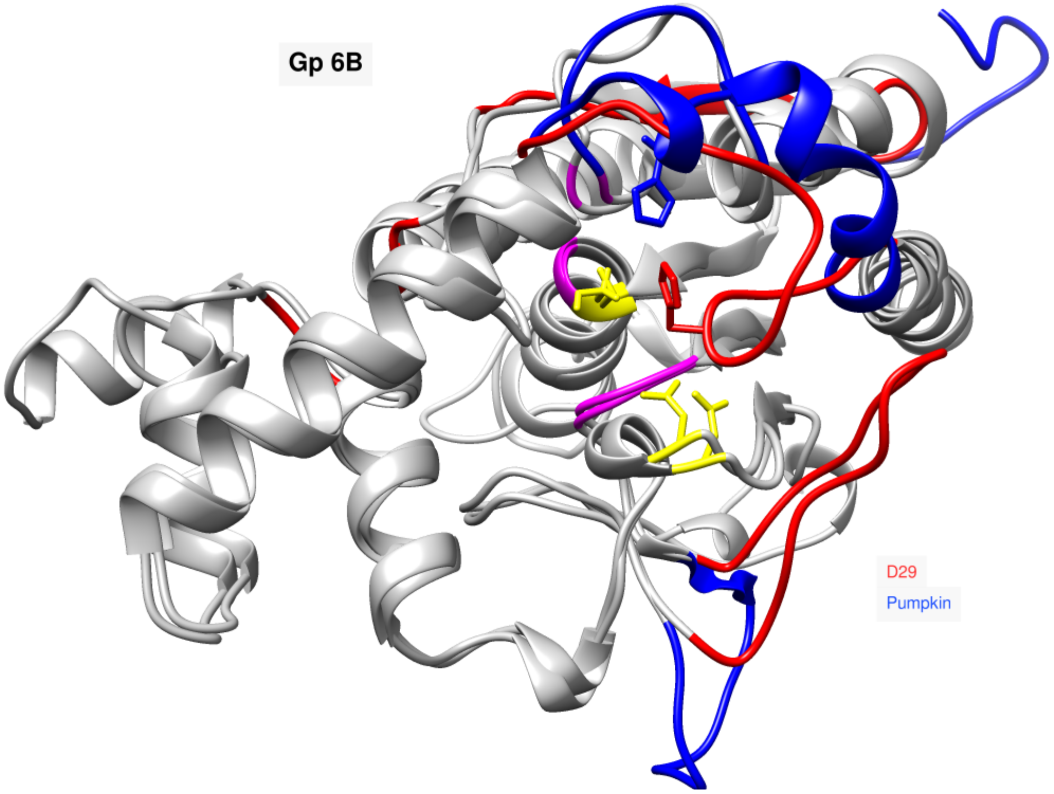

j

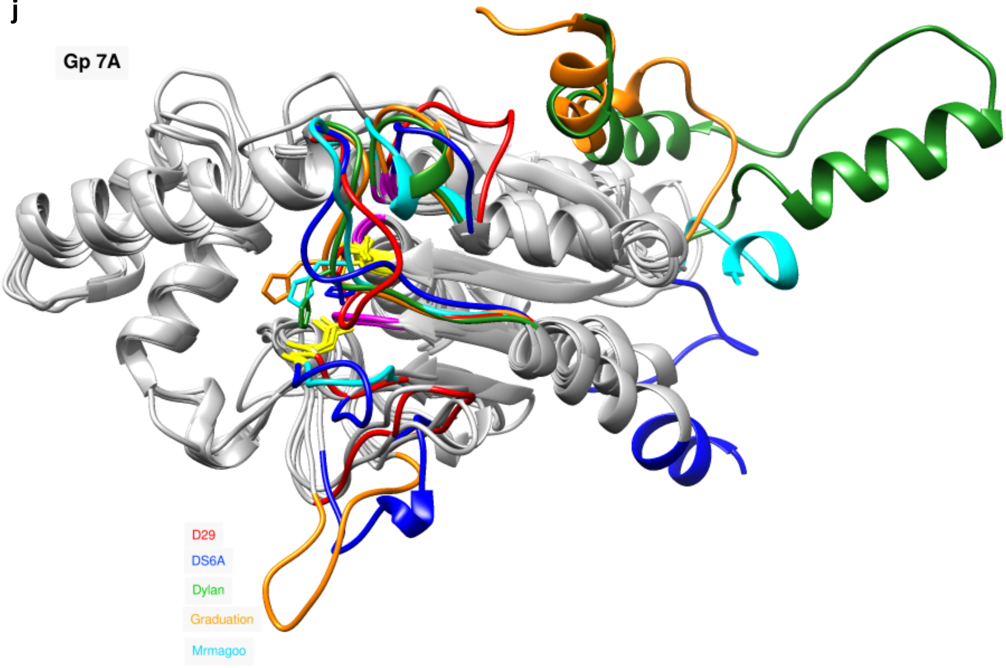

k

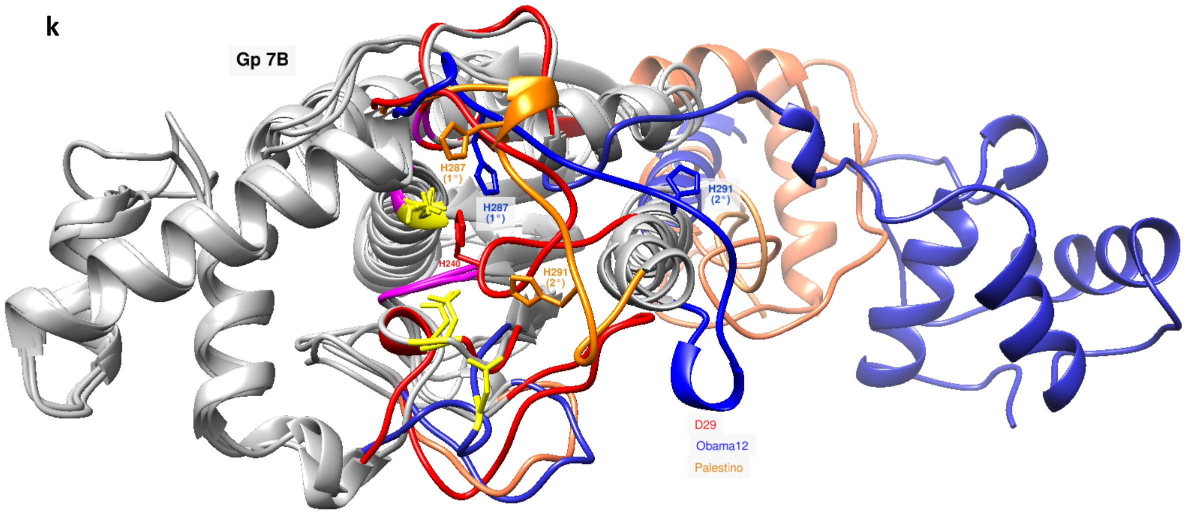
Gp 7B
H287
(1°)
H287
(1°)
H240
H291
(2°)
H291
(2°)
D29
Obama12
Palestino

l

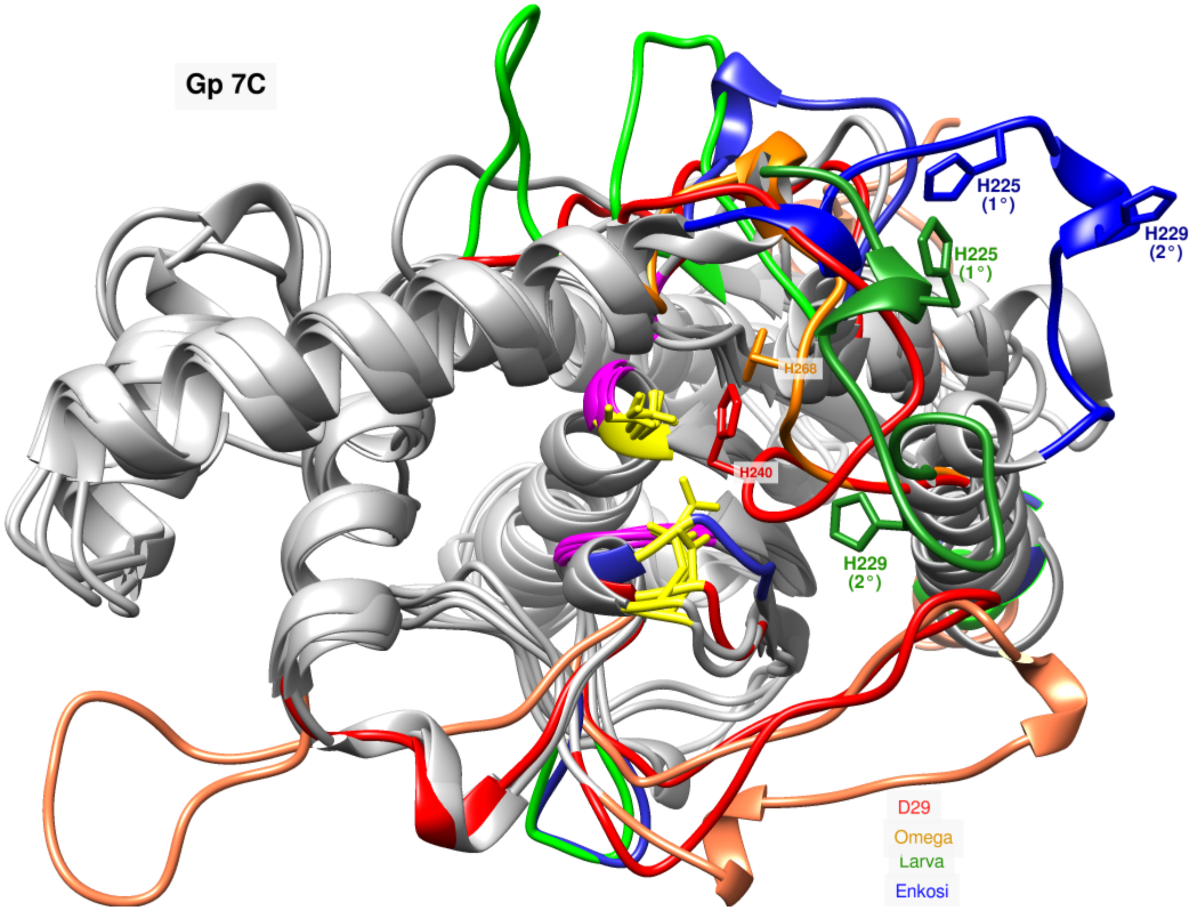
Gp 7C
H225
(1°)
H229
(2°)
H225
(1°)
H268
H240
H229
(2°)
D29
Omega
Larva
Enkosi

Figure S7. Three-dimensional structural alignment of 3D structures of LysB-D29 to members of the seven groups representing its orthologous LysB models, (**a**) Gp-1 models, (**b**) Gp-2A models, (**c**) Gp-2B models, (**d**) Gp-2C models, (**e**) Gp-3 models, (**f**) Gp-4 models, (**g**) Gp-5A and B models, (**h**) Gp-6A models, (**i**) Gp-6B models, (**j**) Gp-7A models, (**k**) Gp-7B model and (**l**) Gp-7C models. The catalytic Ser and Asp residues (yellow color), the catalytic His residue (each in its model's color), oxyanion hole and GNP residues (pink color) and differences in loops 1–5 are highlighted in each of the model's color accordingly.
