# Supplementary material for: Comparative Structural Analysis of Different Mycobacteriophage-Derived Mycolylarabinogalactan Esterases (Lysin B)
Source: Biomolecules. 2019 Dec 27;10(1):45. doi: 10.3390/biom10010045 (PMC7022511; doi:10.3390/biom10010045)
Supplement: Supplementary file 1 [file biomolecules-10-00045-s001.zip › Supplementary/Supp. fig. 8.pdf]

# I. Tunnel conformation

## A. D29 like orientation

1- *Candida rugosa* lipase: Orientation pose 17, *p*-NPP, S= -8.5

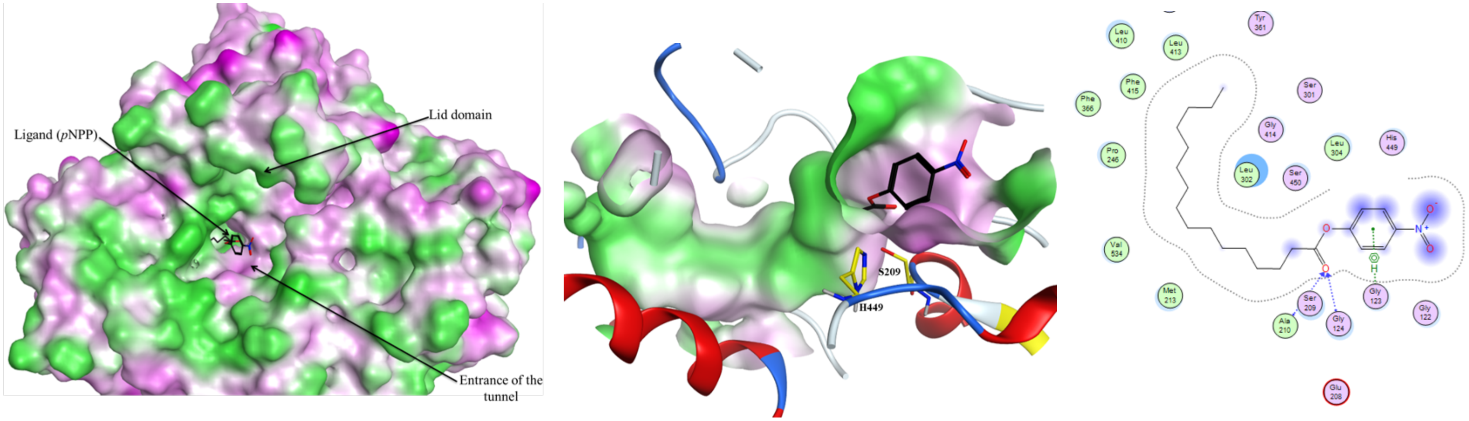


2-LysB-D29: Pose 163, *p*-NPS, S = -8.07

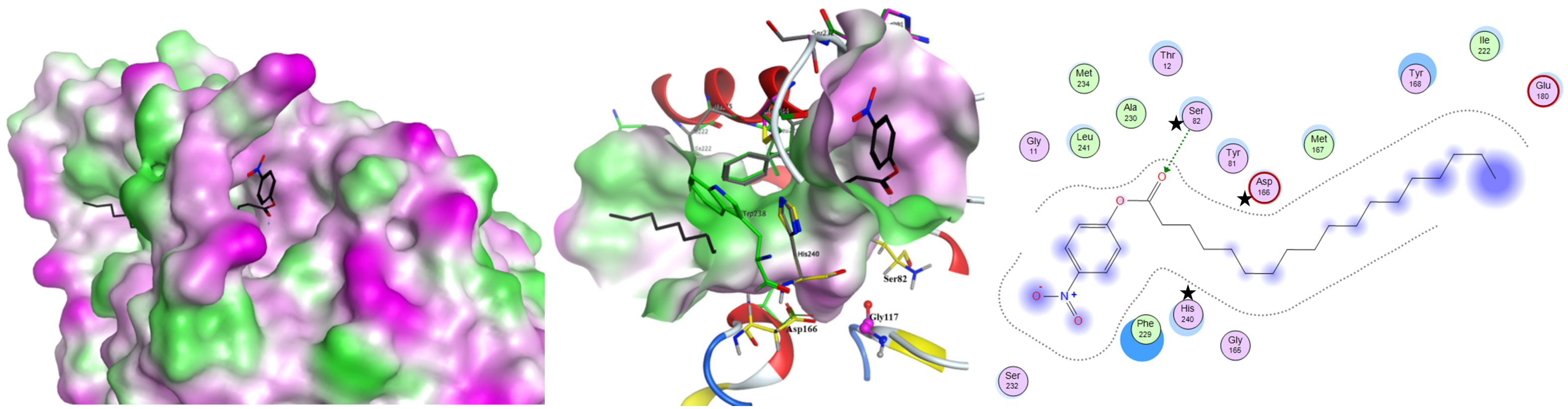

3-LysB-Chy5: Pose 139, *p*-NPP, S = -5.9

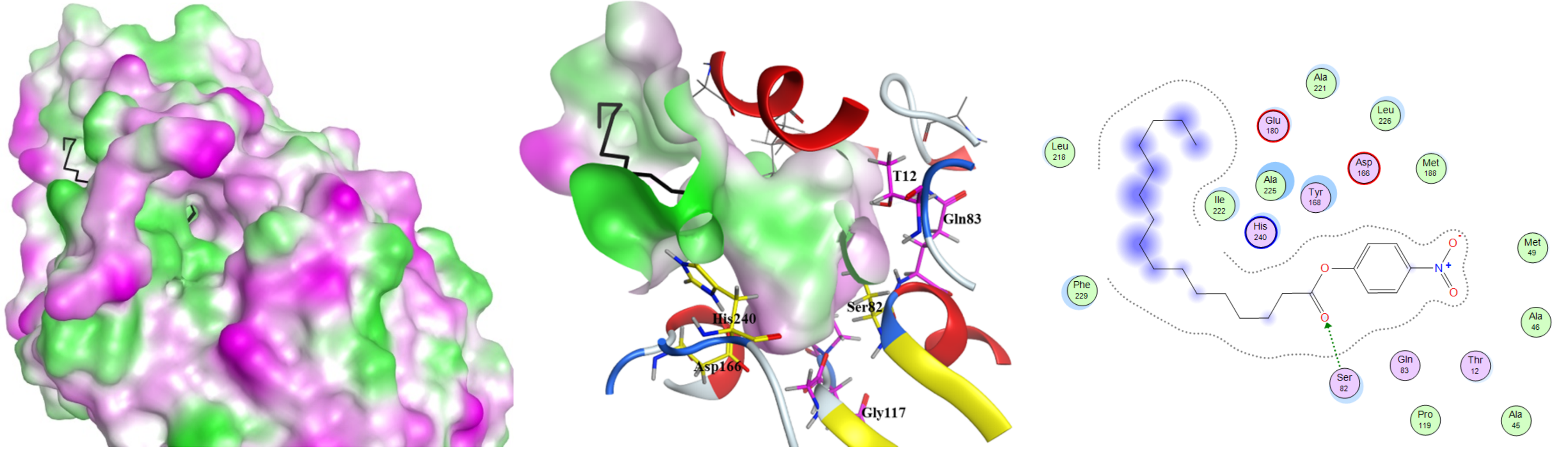


## B. Inverted D29 orientation

1- LysB-SWU-1: Pose 183, *p*-NPS, S = -6.9

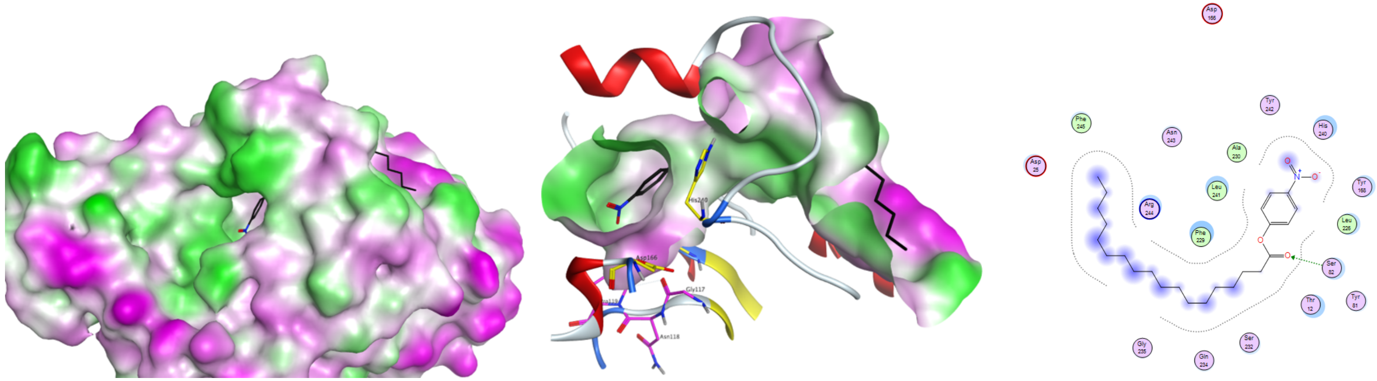

2- LysB-Babyray: NDP, *p*-NPP

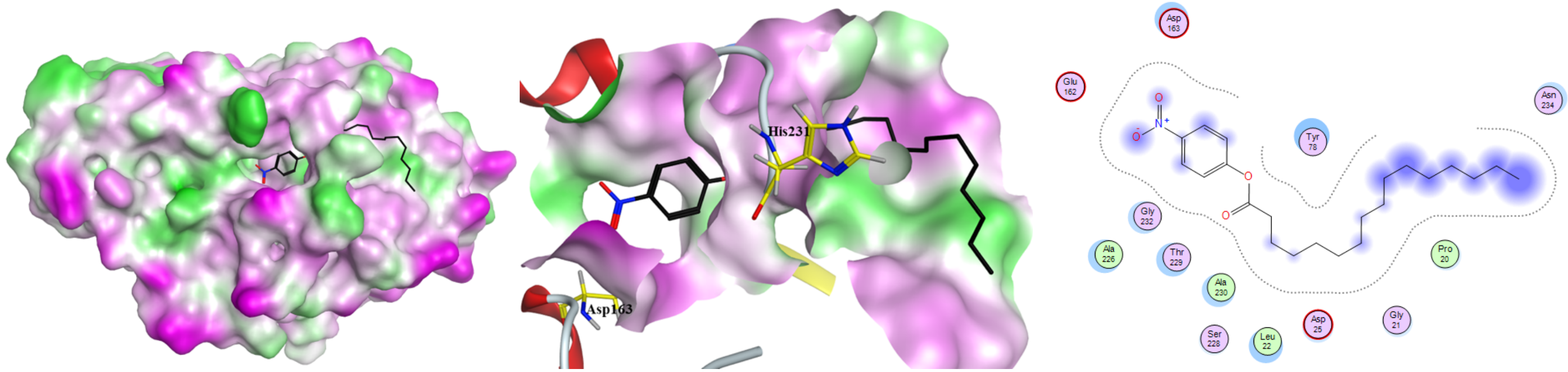


3- LysB-Pumpkin: NDP, *p*-NPS

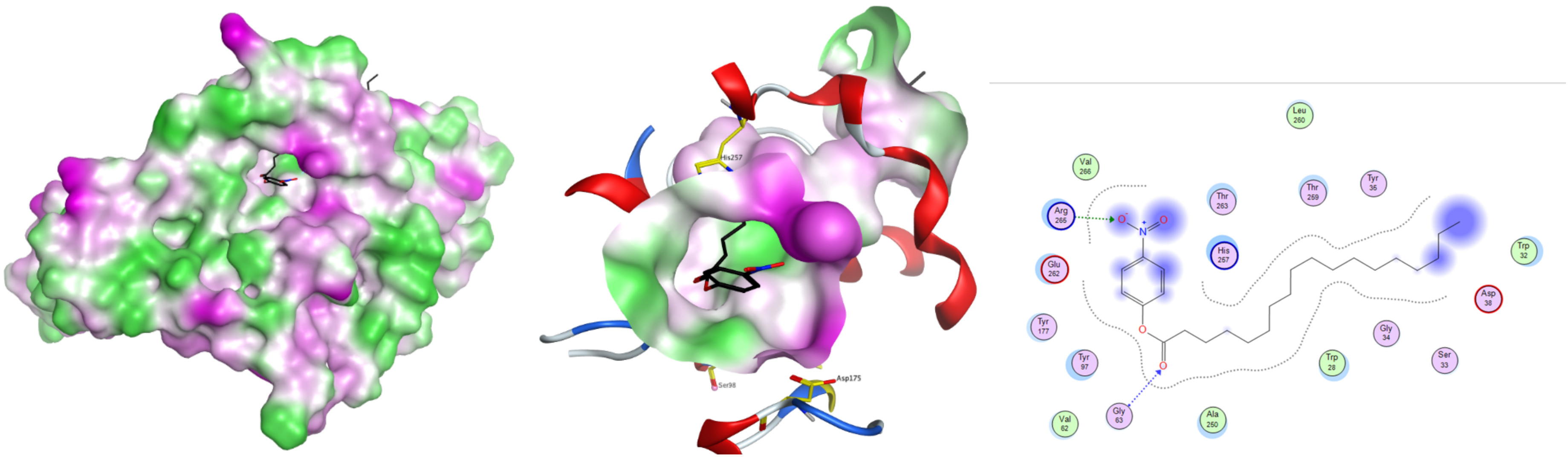

4- LysB-Ms6: Orientation pose 125, *p*-NPP, S= -5.5

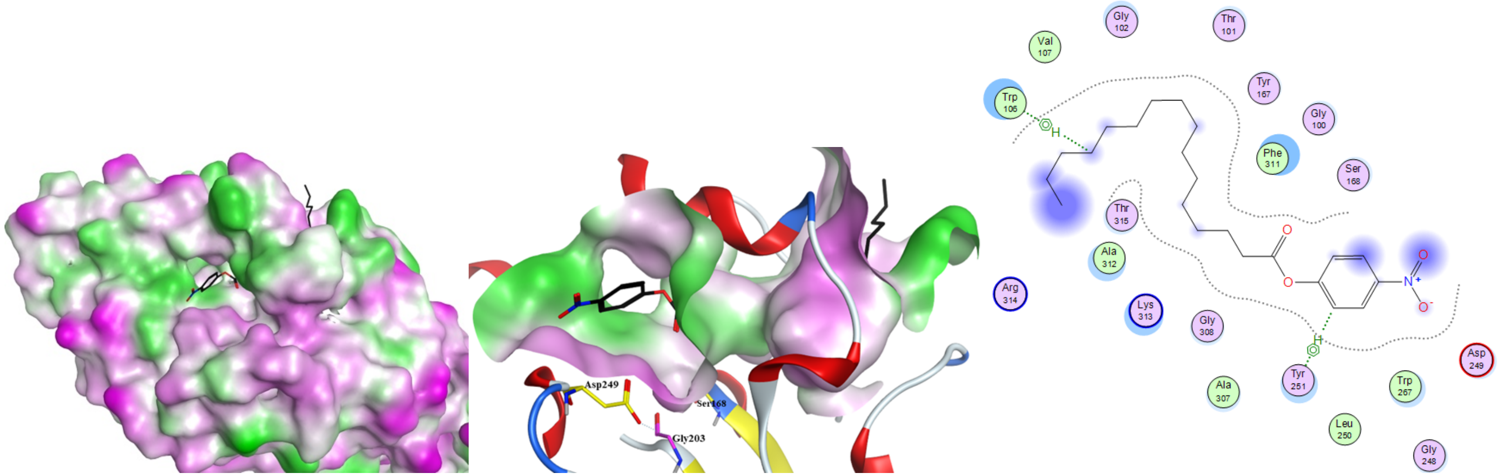


5- LysB-Palestino: Orientation pose 173, *p*-NPS, S= -6.7

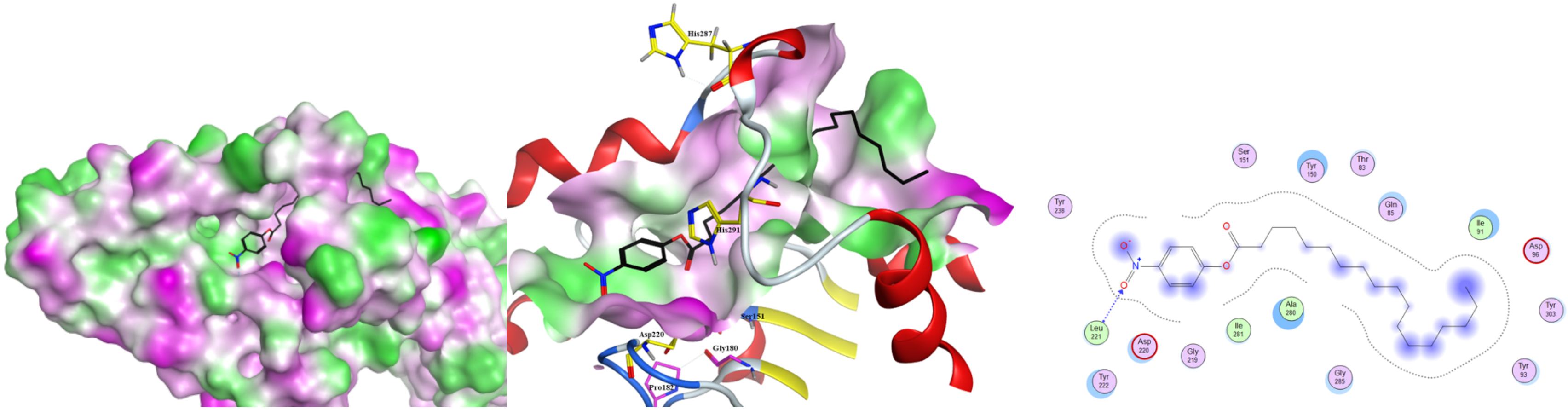


6- LysB-Obama12: Pose 160, *p*-NPP, S= -6.5

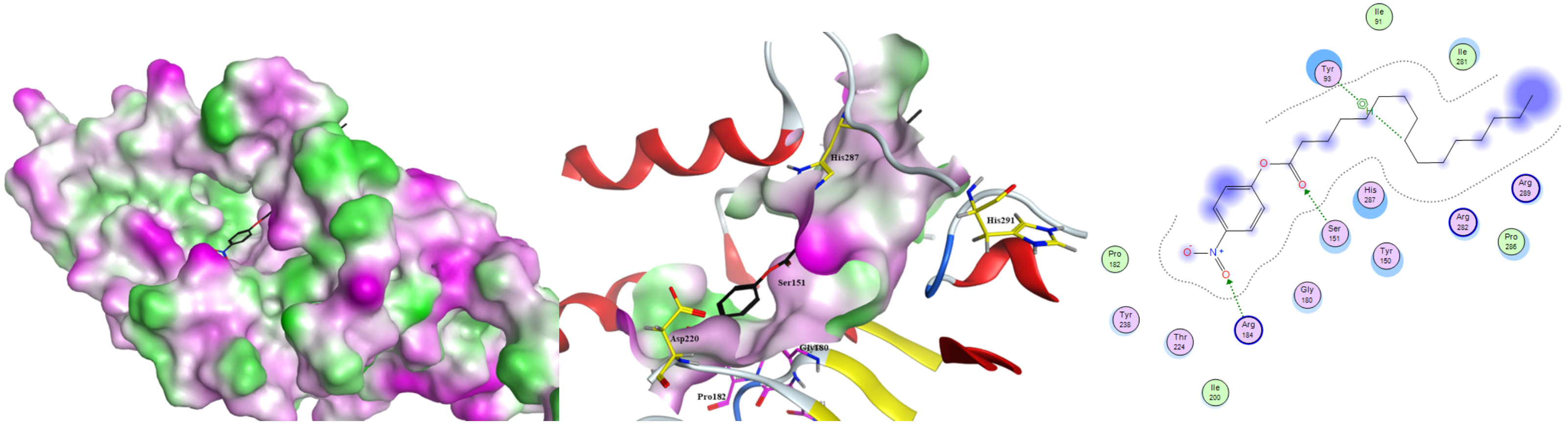


7- LysB-Enkosi: Pose 147, *p*-NPP, S= -6.6

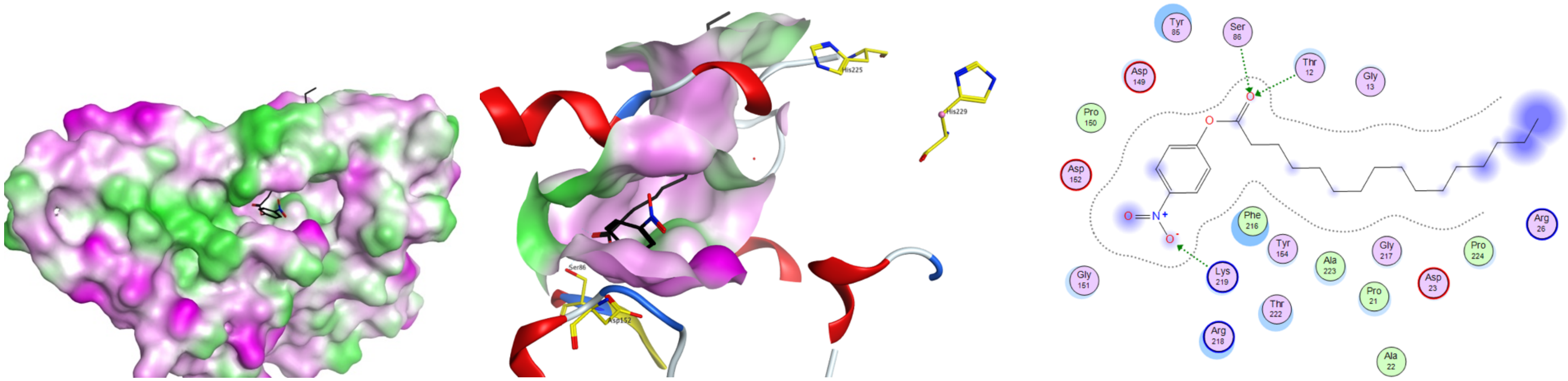

His225
His229
Ser86
Asp152
Tyr 85
Ser 86
Thr 12
Gly 13
Asp 149
Pro 150
Asp 152
Phe 216
Tyr 154
Ala 223
Gly 217
Pro 224
Arg 26
Gly 151
Lys 219
Pro 21
Asp 23
Thr 222
Arg 218
Ala 22

## II. Deep funnel conformation

## A. Very deep funnel

1- *Candida antartica* lipase B: Orientation pose 1, *p*-NPM, S = -7.5

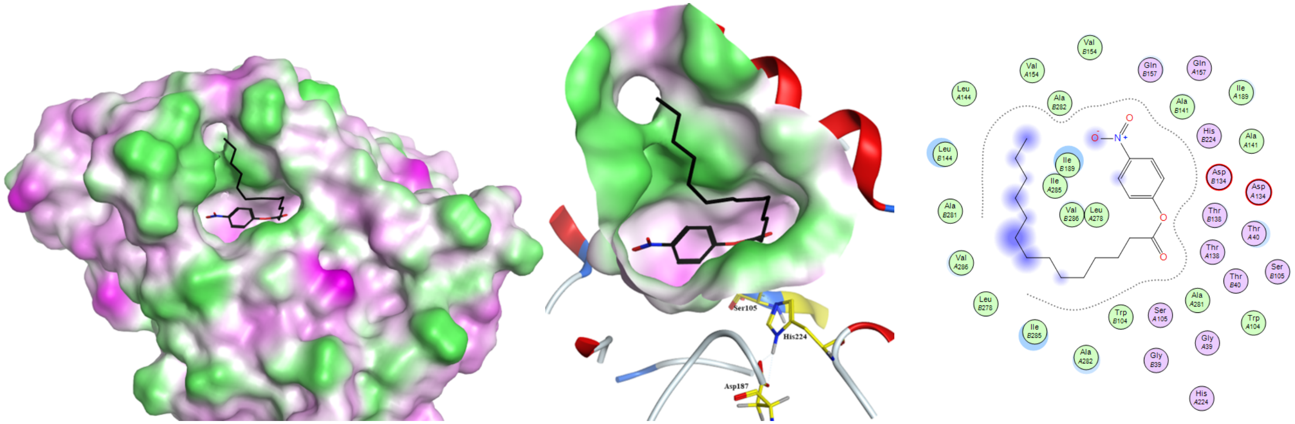


2- LysB-Saal: Orientation pose 165, *p*-NPP, S = -7.5

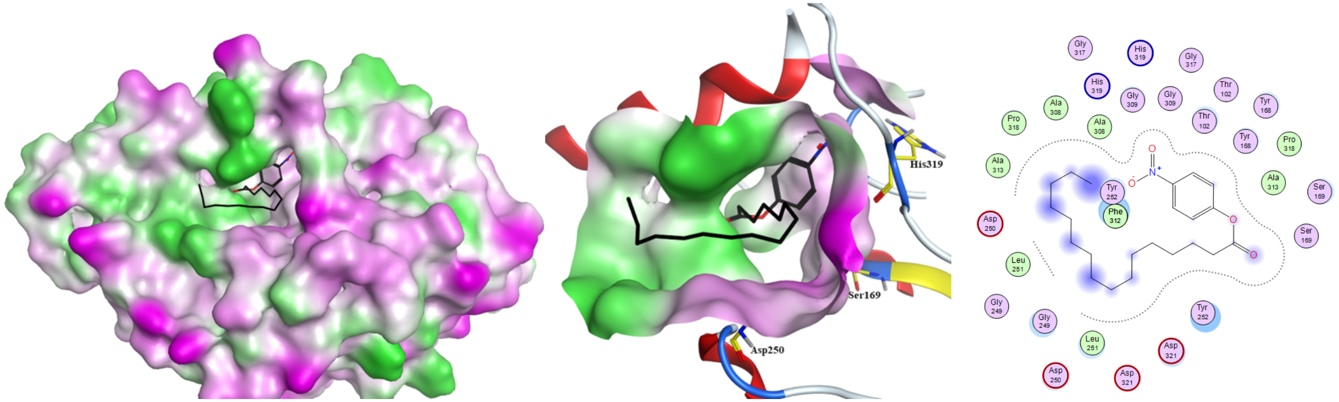

**3-** LysB-DS6A: Orientation pose 160, *p*-NPS, S = -6.1

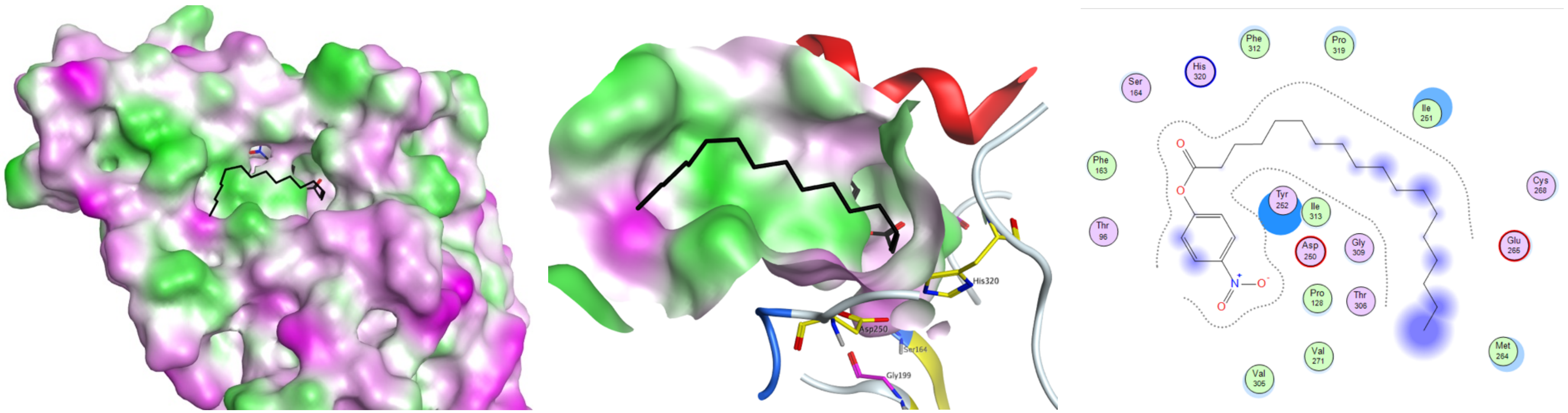

## B. Less deep funnel

*1- Pseudomonas cepacia* lipase: Pose 118, *p*-NPM, S = -7.4

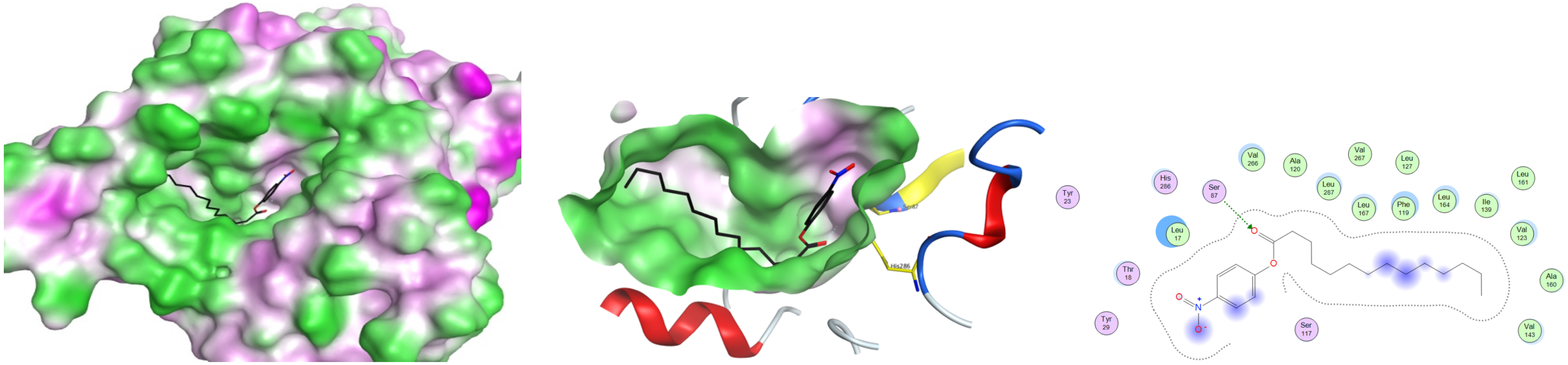


2 -LysB-Hades Orientation pose 104, *p*-NPM, S= -5.8

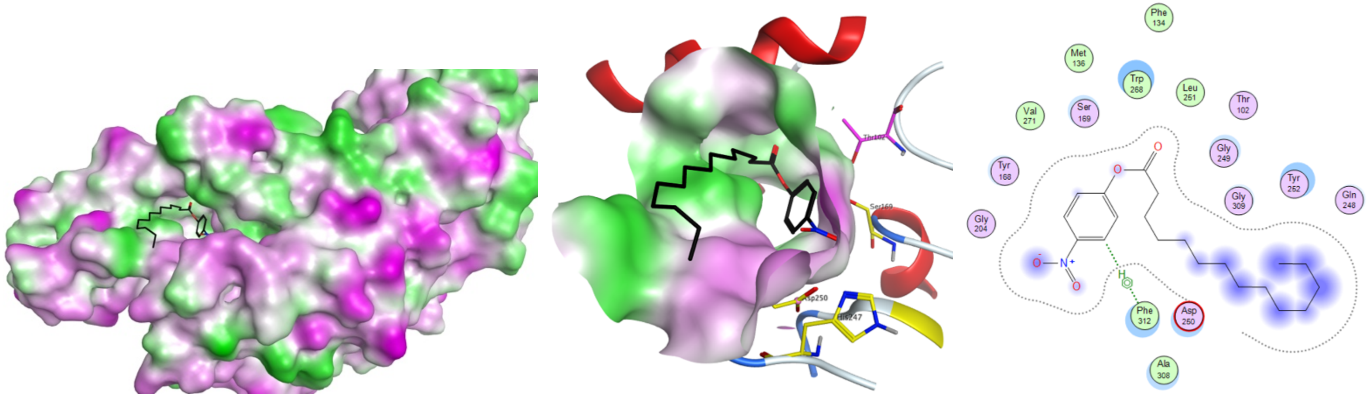

# III. Superficial funnel conformation

1- *Fusarium solani* cutinase: Pose 16, *p*-NPL, S= -5.9

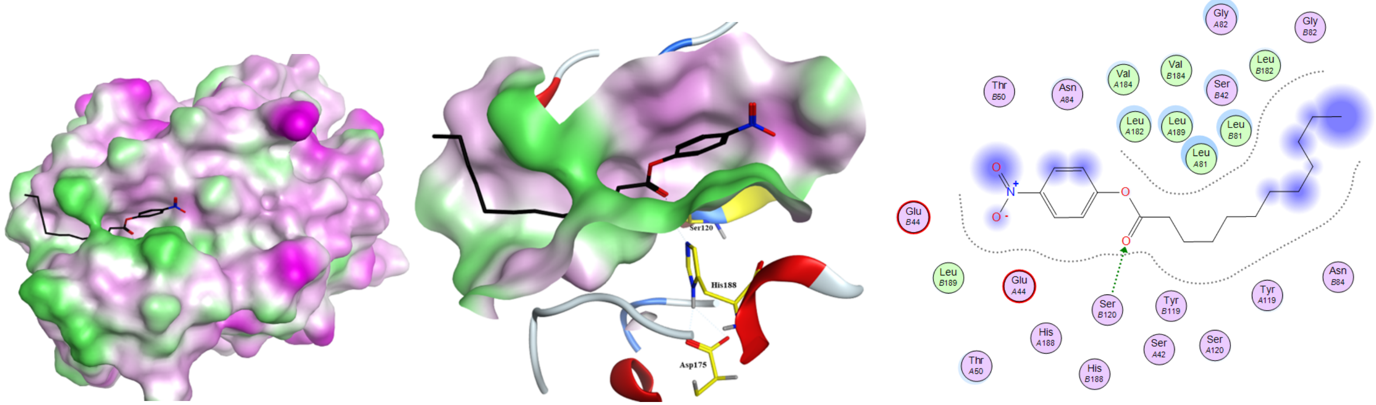


2- Human pancreatic lipase: Orientation pose 14, *p*-NPM, S= -6.6

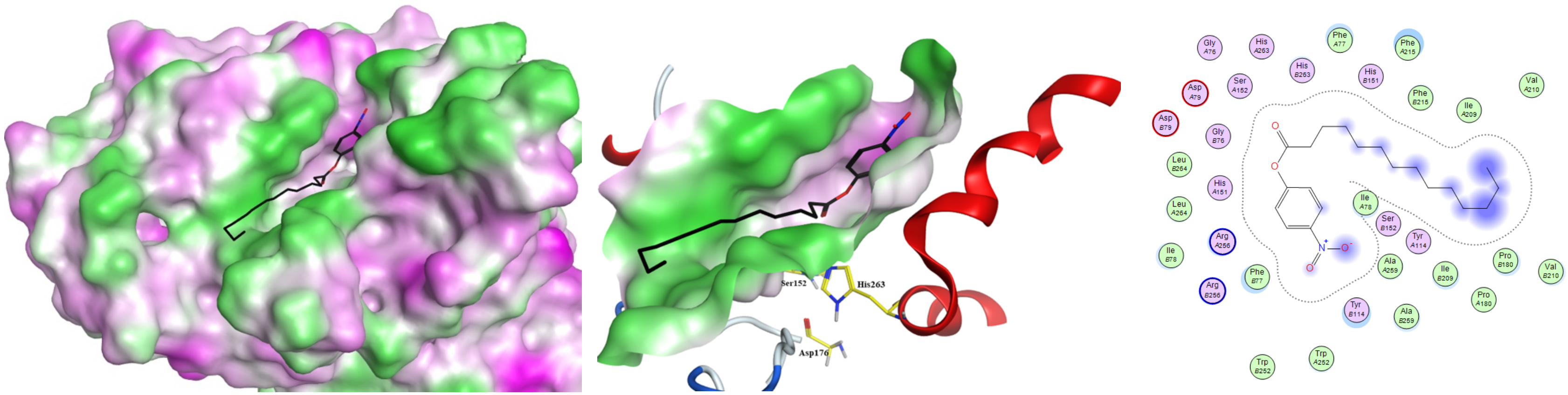

3- LysB-Bxz2: Orientation pose 62, *p*-NPL, S= -6.5

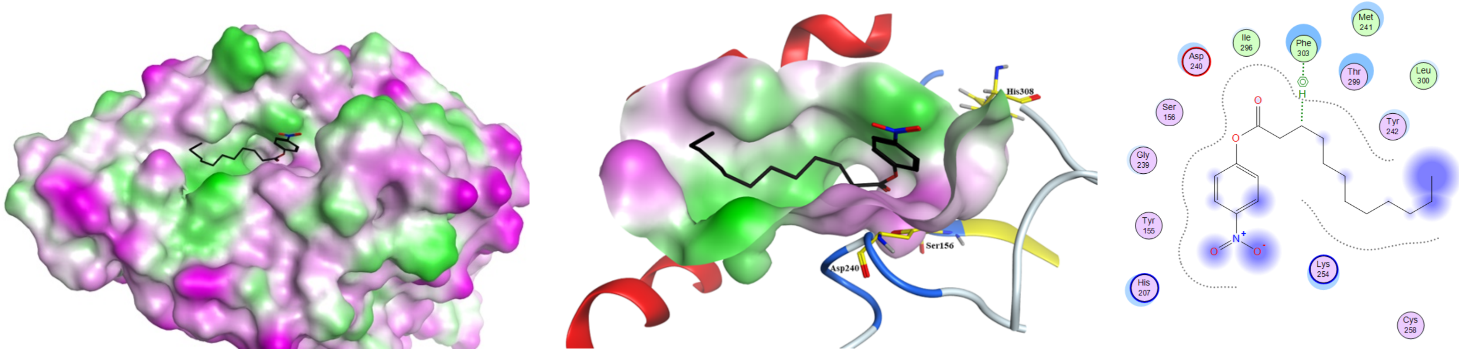


4- LysB-Anubis: Pose 118, *p*-NPM, S= -5.5

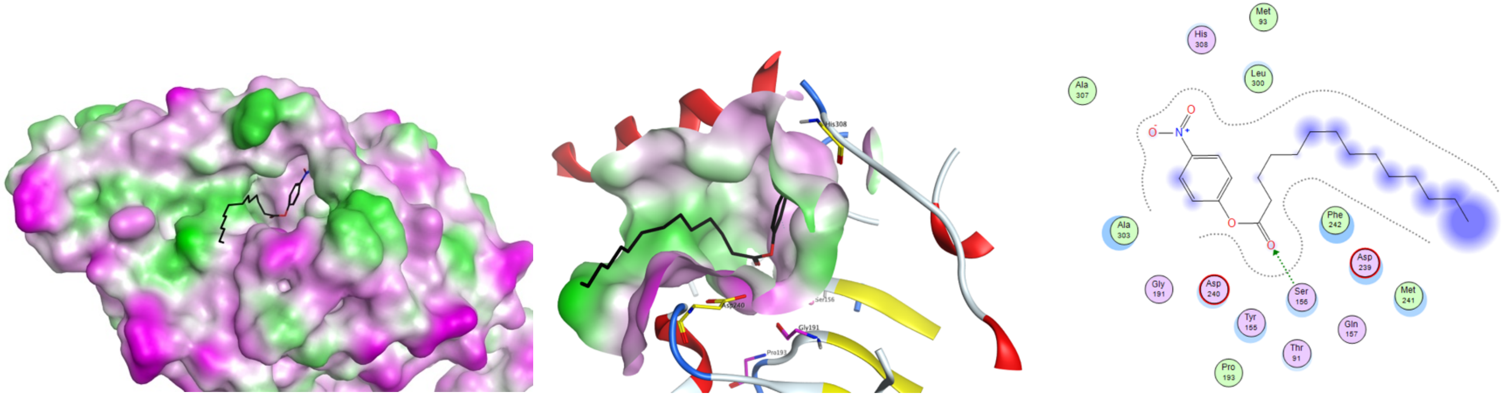

## IV. Long shallow bowl conformation

1- *Rhizomucor miehei* lipase: Orientation pose 8, *p*-NPS, S= -4.4

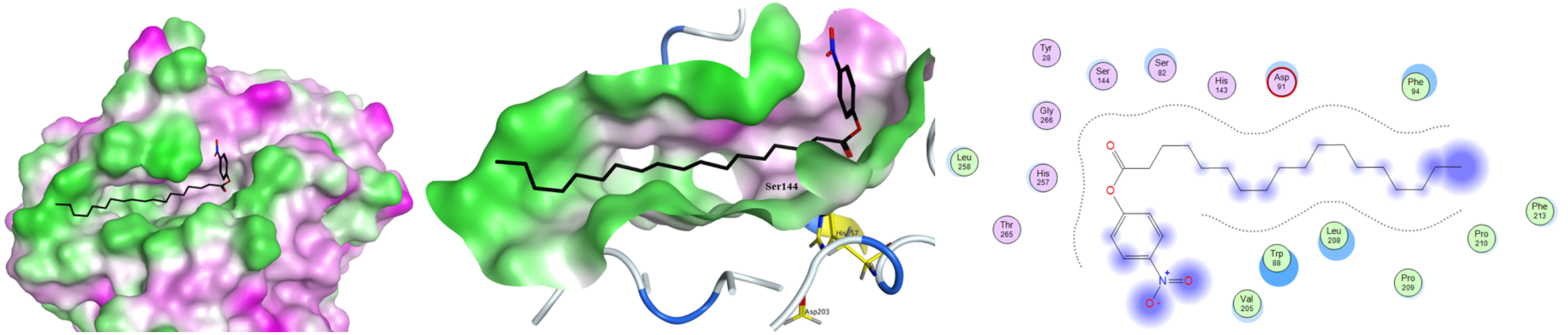


1- LysB-Omega: NDP, *p*-NPP

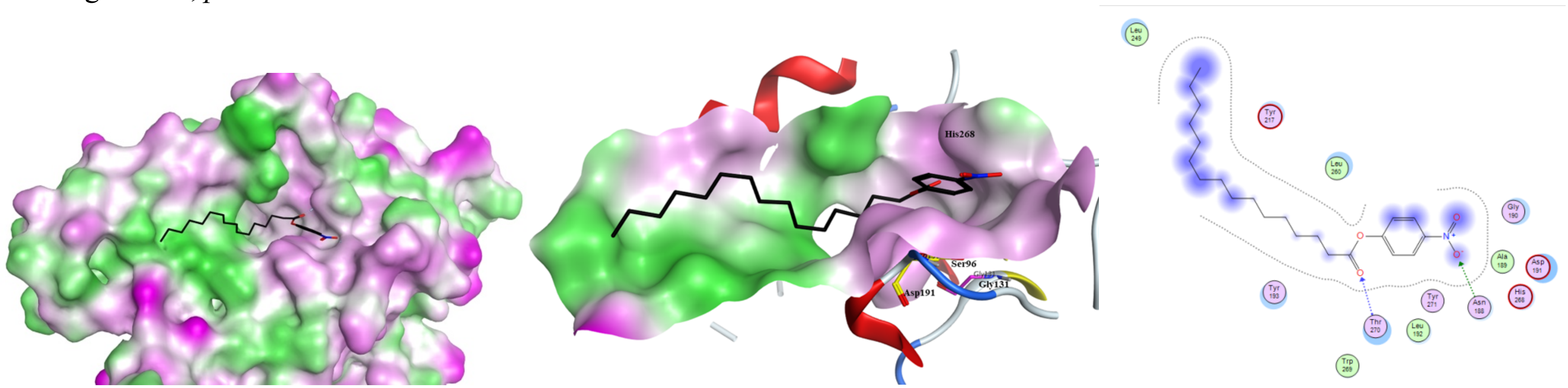

## V. Narrow buried cave conformation

1- Acetylcholine esterase: Orientation pose 36, *p*-NPL, S= - 10.06

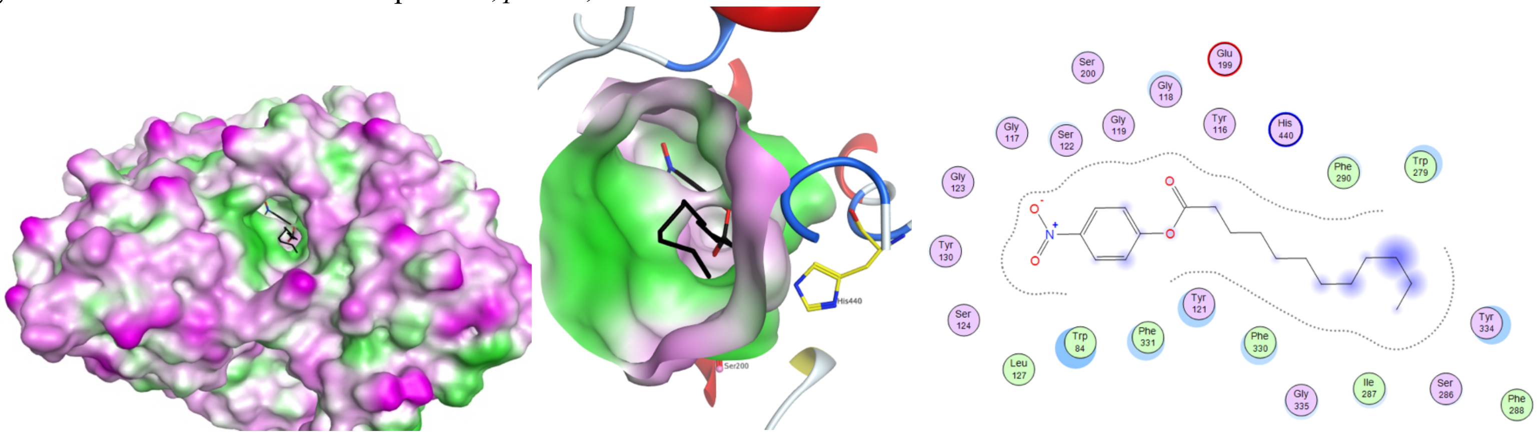


2-LysB-Goose: Orientation pose 36, *p*-NPD, S= - 6.3

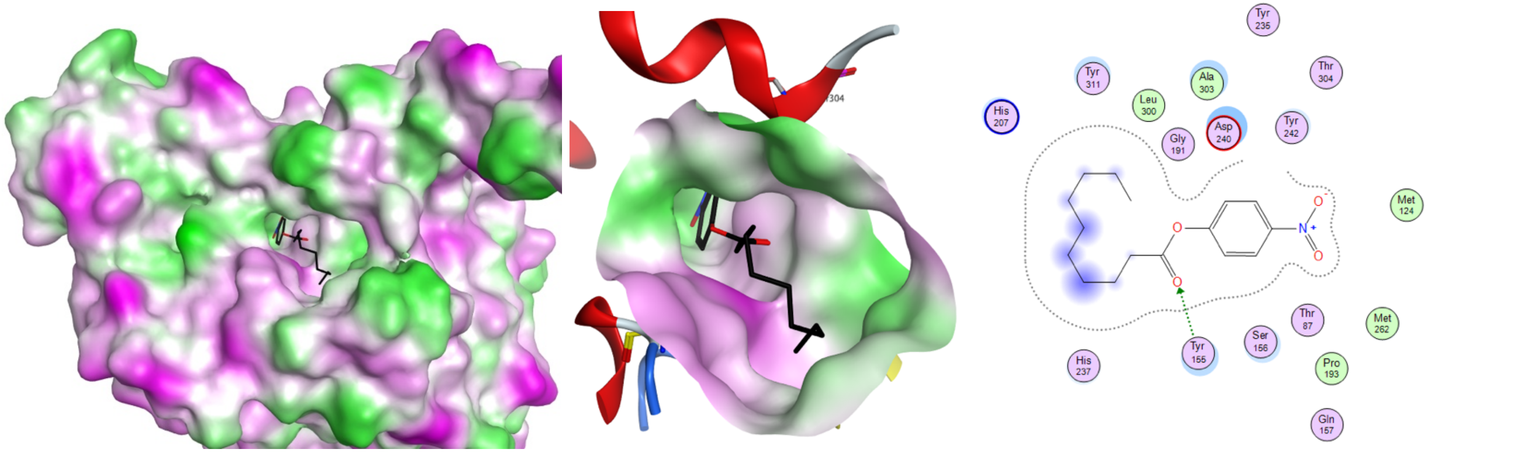

**Figure S8**: 3D conformations of poses of *p*-NP ligands upon docking to LysB proteins and other lipases and cutinases. Each diagram illustrates (in order) the overall surface of protein with its ligand, the shape of active site with the docked ligand, the interactions of ligand atoms with different residues of its protein active site. Hydrophilic residues (pink color), hydrophobic residues (green color), *p*-NP ligands (Black color). Orientation pose: ligand-protein conformation where the catalytic Ser faces ligand's ester bond (C=O) with no H-bond formation. NDP: (No Detected Pose) neither binding nor orientation pose were detected. Stars indicate catalytic triad residues of LysB-D29.
