# Supplementary material for: Comparative Structural Analysis of Different Mycobacteriophage-Derived Mycolylarabinogalactan Esterases (Lysin B)
Source: Biomolecules. 2019 Dec 27;10(1):45. doi: 10.3390/biom10010045 (PMC7022511; doi:10.3390/biom10010045)
Supplement: Supplementary file 1 [file biomolecules-10-00045-s001.zip › Supplementary/Supp. fig. 9.pdf]

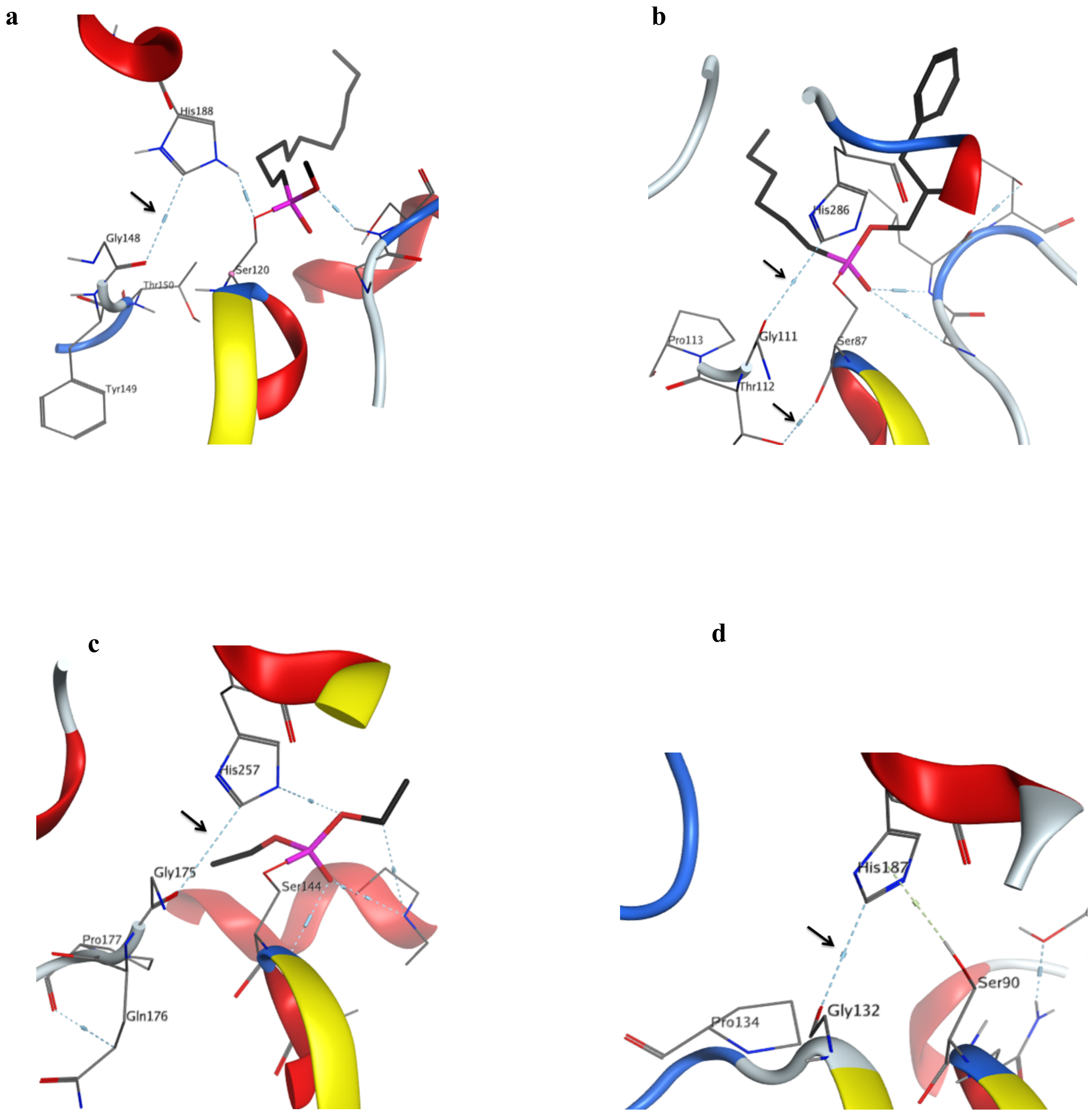
a
His188
Gly148
Thr150
Ser120
Tyr149
b
His286
Gly111
Pro113
Ser87
Thr112
c
His257
Gly175
Ser144
Pro177
Gln176
d
His187
Gly132
Pro134
Ser90

e

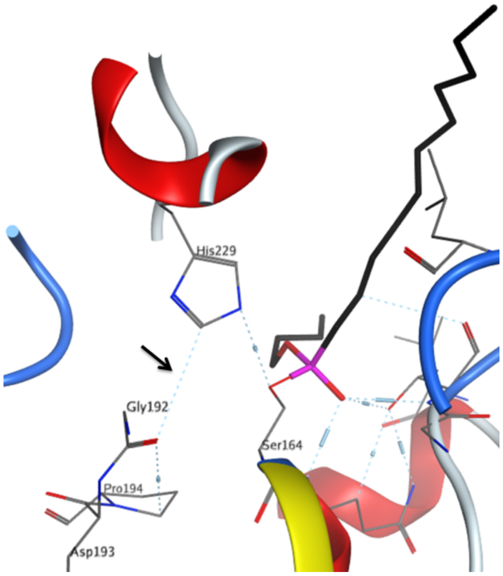

f

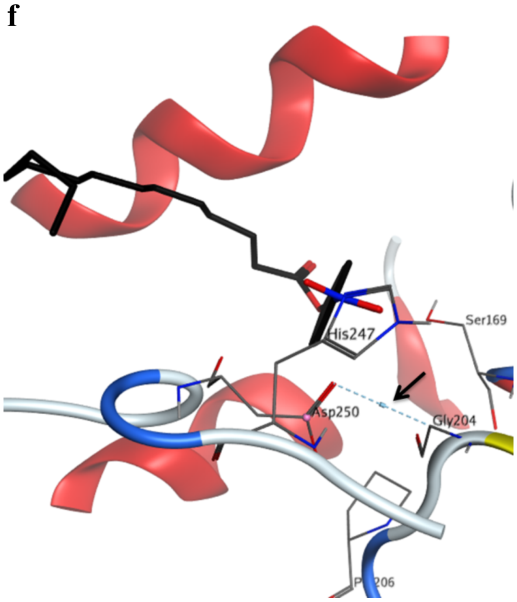

g

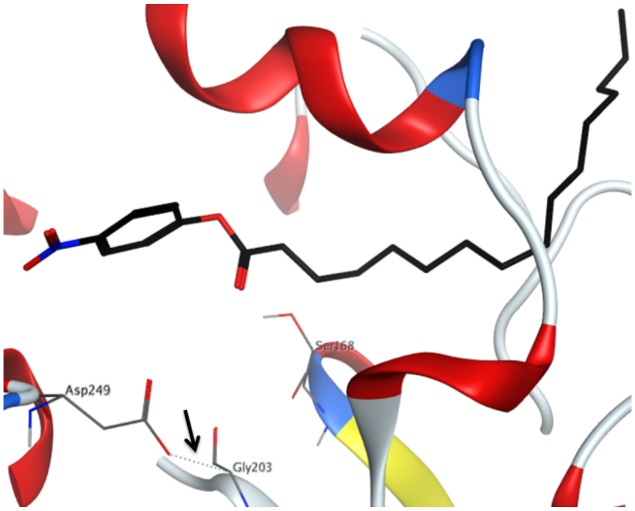

Figure S9: Three dimensional conformations of active sites of (**a**) *F. solani* cutinase, (**b**) *P. cepacia* lipase, (**c**) *R. miehei* lipase, (**d**) *P. purpureogenum* Acetylxylan esterase (**e**), *T. reesei* cutinase (**f**), LysB-Hades and (**g**) LysB-Ms6 showing the H-bonds between glycine residue of the GNP motif with members of the catalytic triad: Histidine residues (**a**-**e**) and Aspartate residues (**f**-**g**) (pointed by black arrows). Co-crystalized inhibitors and *p*-NP ligands (black color).
