# Supplementary material for: Comparative Structural Analysis of Different Mycobacteriophage-Derived Mycolylarabinogalactan Esterases (Lysin B)
Source: Biomolecules. 2019 Dec 27;10(1):45. doi: 10.3390/biom10010045 (PMC7022511; doi:10.3390/biom10010045)
Supplement: Supplementary file 1 [file biomolecules-10-00045-s001.zip › Supplementary/Supplementary Tables.pdf]

Table S1: Evaluation parameters of the generated LysB homology models.

No.	Name	No. of generated models by YASARA	YASARA Z-score[a] for hybrid model	Verify-3D[b]	ERRAT quality factor[c]	Prove[d]	Ramachandran (% residues in favored and allowed regions)	Ramachandran (% residues in the disallowed regions)	ProSA Z-score[e]
	**D29**	---------	---------	98.02%	97.95	0.9%	99%	1.0%	-7.62
1	**Chy5**	18	0.015	96.06%	98.37	3.6%	99.50%	0.5%	-7.74
2	**SWU1**	17	0.041	92.46%	93.44	2.3%	99.1%	0.9%	-7.57
3	**Echild**	2	-0.399	96.99%	98.03	3.2%	99.6%	0.4%	-8.18
4	**RedRock**	11	-0.575	85.54%	94.95	3.2%	99.6%	0.4%	-8.27
5	**Goose**	2	-0.439	97.67%	98	3.3%	98.6%	1.4%	-8.14
6	**Twister**	17	-0.293	99.61%	93.2	3.6%	99.1%	0.9%	-8.12
7	**Sheen**	16	-0.506	96.75%	94.95	2.5%	99.0%	1.0%	-7.54
8	**Timshel**	7	-0.590	86.46%	95.87	2.9%	99.3%	0.7%	-9.11
9	**HelDan**	46	-0.299	97.56%	95.79	3.1%	99.5%	0.5%	-7.26
10	**BabyRay**	70	-0.196	92.56%	88.03	5.0%	98.1%	1.9%	-7.21
11	**BTCU-1**	16	-0.501	99.19%	94.11	3.3%	99.1%	0.9%	-7.61
12	**Anubis**	14	-0.462	100.00%	95.88	2.5%	99.5%	0.5%	-8.23
13	**Bxz2**	21	-0.628	82.24%	100	2.3%	99.3%	0.7%	-7.01
14	**Severus**	21	-0.781	91.67%	93.98	3.3%	98.1%	1.9%	-8.45
15	**Zakai**	2	-0.896	83.40%	98.37	3.3%	98.6%	1.4%	-6.59
16	**Wildcat**	16	-0.934	91.19%	93.25	2.4%	99.5%	0.5%	-5.83
17	**Ardmore**	5	-1.369	87.65%	98.34	4.1%	98.6%	1.4%	-7.24

18	**MS6**	5	-1.369	88.45%	98.34	4.1%	98.6%	1.4%	-7.22
19	**Hades**	24	-1.645	80.18%	92.83	2.5%	98.9%	1.1%	-6.76
20	**Saal**	5	-1.611	86.06%	95.45	5.3%	98.6%	1.4%	-7.9
21	**Pumpkin**	6	-1.535	76.53%	97.76	4.9%	98.3%	1.7%	-5.49
22	**Graduation**	9	-1.199	83.58%	94.14	3.2%	99.1%	0.9%	-6.54
23	**MrMagoo**	16	-0.507	82.69%	92.85	2.8%	99.1%	0.9%	-6.86
24	*Dylan*	5	-1.235	94.55%	96.38	3.4%	98.6%	1.4%	-7.61
25	**DS6A**	14	-1.502	86.35%	95.03	3.7%	98.6%	1.4%	-6.48
26	**Palestino**	7	-2.245	81.37%	87.7	5.1%	99.6%	0.4%	-5.76
27	**Obama12**	46	-1.613	67.79%	97.48	3.3%	98.1%	1.9%	-5.61
28	**Omega**	39	-1.873	90.34%	98.93	2.9%	99.2%	0.8%	-8.31
29	**Larva**	79	-1.938	77.87%	91.32	2.7%	99.0%	1.0%	-5.9
30	**Enkosi**	89	-1.746	83.73%	97.11	3.4%	98.5%	1.5%	-6.69

[a] Z-score describes how many standard deviations the model quality is away from the average high-resolution X-ray structure. Higher values are better, negative values indicate that the homology model looks worse than a high-resolution X-ray structure. The overall score thus captures the correctness of backbone- (Ramachandran plot) and side-chain dihedrals, as well as packing interactions as it is calculated from the equation: Overall Z-score = 0.145*Dihedrals + 0.390*Packing1D + 0.465*Packing3D

[b] Verify_3D: Determines the compatibility of an atomic model (3D) with its own amino acid sequence (1D) by assigning a structural class based on its location and environment (alpha, beta, loop, polar, nonpolar etc.) and comparing the results to good structures. The model is regarded "Pass" if at least 80% of the amino acids have scored >= 0.2 in the 3D/1D profile.

[c] ERRAT: Expressed as the percentage of the protein for which the calculated error value falls below the 95% rejection limit. Good high resolution structures generally produce values around 95% or higher. For lower resolutions (2.5 to 3A) the average overall quality factor is around 91%.

[d] Prove: Calculates the volumes of atoms in macromolecules using an algorithm which treats the atoms like hard spheres and calculates a statistical Z-score deviation for the model from highly resolved (2.0 Å or better). It records "ERROR" if the %Buried outlier protein atoms exceed 5%.

[e] ProSA Z-score: indicates the overall model quality in comparison to z-scores of all experimentally determined protein chains in current PDB. It can be used to check whether the z-score of the input structure is within the range of scores typically found for native proteins of similar size.

Table S2: LysB homologous proteins representatives of the seven groups, chain length and domain diversity.

No	LysB-	Uniprot ID	(%) Similarity to LysB-D29	Group	Total protein Length	Domains, protein families and residues	No. of domains
	**D29**	O64205	100	Standard	254	[PE-PPE, (PF08237); 51-125]<br>[Cutinase, (PF01083); 41-169]	2
**1**	**Chy5**	R4JF81	99.2	1	254	[PE-PPE, (PF08237); 51-127]<br>[Cutinase, (PF01083); 41-169]	2
**2**	**SWU-1**	I1V1E2	90.6			[PE-PPE, (PF08237); 51-125]<br>[Cutinase, (PF01083); 41-169]	2
**3**	**Echild**	W0LJP4	76.6	2A	325	[PE-PPE, (PF08237); 109-191]<br>[Cutinase, (PF01083); 109-238]<br>[Flagellar hook-length control protein FliK (PF02120); 202-230]	3
**4**	**RedRock**	D3JZ76	74.5		325	[PE-PPE, (PF08237); 109-192]<br>[Cutinase, (PF01083); 109-240]	2
**5**	**Goose**	K0G173	76.6	2B	324	[PE-PPE, (PF08237); 126-199]<br>[Cutinase, (PF01083); 116-245]	2
**6**	**Twister**	H9NCI8	75.8		324	[PE-PPE, (PF08237); 126-198]<br>[Cutinase, (PF01083); 116-249]<br>[Uncharacterized protein family (UPF0565); 139-189]	3
**7**	**Sheen**	A0A0B5A441	70.2	2C	246	[PE-PPE, (PF08237); 51-119]<br>[Cutinase, (PF01083); 69-162]	2
**8**	**Timshel**	G1DB29	70.2		325	[PE-PPE, (PF08237); 130-198]<br>[Cutinase, (PF01083); 148-207]	2
**9**	**HelDan**	G1DA59	63	3	246	[PE-PPE, (PF08237); 50-122]<br>[Cutinase, (PF01083); 41-123]<br>[Phospholipase/Carboxylesterase (PF02230); 61-88]	3

10	**BabyRay**	A0A1D8EW72	63.1		244	[PE-PPE, (PF08237); 50-122]<br>[Cutinase, (PF01083); 41-131]<br>[Thioesterase domain (PF00975); 62-84]	3
11	**BCTU-1**	R9R488	63.0		246	[PE-PPE, (PF08237); 49-122]<br>[Cutinase, (PF01083); 41-132]<br>[Thioesterase domain (PF00975); 62-84]	3
12	**Anubis**	A0A097BYL0	62.9		321	[PE-PPE, (PF08237); 130-199]<br>[Cutinase, (PF01083); 149-200]	2
13	**Bxz2**	Q857L1	63.3	4	321	[PE-PPE, (PF08237); 130-199]<br>[Cutinase, (PF01083); 149-200]	2
14	**Severus**	R4JQ45	58.8		321	[PE-PPE, (PF08237); 122-199]<br>[Cutinase, (PF01083); 149-268]<br>[Protein of unknown function (PF05338); 283-311]	3
15	**Zakai**	A0A1D8EPB0	48.8	5A	342	[Protein of unknown function (PF05338); 80-94]<br>[PE-PPE, (PF08237); 143-218]<br>[Cutinase, (PF01083); 129-220]	3
16	**Wildcat**	Q19Y08	47.0	5B	321	[Putative peptidoglycan binding domain (PF01471); 17-71]<br>[PE-PPE, (PF08237); 131-202]<br>[Cutinase, (PF01083); 117-169]	3
17	**Ardmore**	D4N7H5	42.9		332	[Putative peptidoglycan binding domain (PF01471); 15-58]<br>[PE-PPE, (PF08237); 134-224]<br>[Cutinase, (PF01083); 120-208]	3
18	**MS6**	Q9FZR9	42.9	6A	333	[Putative peptidoglycan binding domain (PF01471); 15-58]<br>[PE-PPE, (PF08237); 134-224]<br>[Cutinase, (PF01083); 120-208]	3
19	**Hades**	A0A076YRQ3	42.9		332	[Statherin (PF03875); 14-43]<br>[Putative peptidoglycan binding domain (PF01471); 8-59]<br>[PE-PPE, (PF08237); 136-225]<br>[Cutinase, (PF01083); 120-208]	4

**20**	**Saal**	W6AV51	41.7		333	[Putative peptidoglycan binding domain (PF01471); 38-58]<br>[PE-PPE, (PF08237); 136-224]<br>[Cutinase, (PF01083); 120-208]	3
**21**	**Pumpkin**	C9DCK3	41.7	6B	285	[PE-PPE, (PF08237); 52-110]<br>[Cutinase, (PF01083); 49-177]	2
**22**	**Graduation**	V5R3P2	39.1	7A	322	[PE-PPE, (PF08237); 123-205]<br>[Cutinase, (PF01083); 115-240]	2
**23**	**MrMagoo**	A0A1L6BYH7	38.6		325	[Putative peptidoglycan binding domain, (PF01471); 26-70]<br>[PE-PPE, (PF08237); 118-207]<br>[Cutinase, (PF01083); 110-188]<br>[Lipase (class 3), (PF01764); 130-186]<br>[Protein of unknown function, (PF11288); 147-194]<br>[Bacterial virulence protein (VirJ), (PF06057); 133-167]<br>[Molybdopterin oxidoreductase N-terminal, (PF18364); 286-315]	7
**24**	**Dylan**	S5ZH98	37.2		346	[PE-PPE, (PF08237); 135-232]<br>[Cutinase, (PF01083); 128-225]<br>[Phospholipase/Carboxylesterase, (PF02230); 169-194]<br>[Protein of unknown function, (PF11288); 165-198]	4
**25**	**DS6A**	G8I4E1	37.4		345	[Protein of unknown function (PF11288); 68-117]<br>[PE-PPE, (PF08237); 133-219]<br>[Cutinase, (PF01083); 119-206]	3
**26**	**Palestino**	A0A1J0MAW0	36.4	7B	326	[PE-PPE, (PF08237); 91-187]	1
**27**	**Obama12**	W0LJG8	35.5		326	No conserved motif	0
**28**	**Omega**	Q854L1	31.3	7C	290	[Cutinase, (PF01083); 21-139]	1
**29**	**Larva**	G1FMT3	31.1		253	[Cutinase, (PF01083); 76-122]	1
**30**	**Enkosi**	A0A0M4R3S7	30.1		252	No conserved motif	0

Table S3: Types of active site conformations of LysB models and the best docking score upon docking of *p*–NP ligands.

No.	LysB-	Length of loop-5[a] (aa)	*p*-NP ligands							Presence of GNP in the active site
			C4 *p*-NPB	C8 *p*-NPC	C10 *p*-NPD	C12 *p*-NPL	C14 *p*-NPM	C16 *p*-NPP	C18 *p*-NPS	
**I. Tunnel conformation**										
**A. lysB-D29-like orientation**										
	**D29[b]**	14	Pose: 4 S:(-5.41)	Pose: 22 S:(-6.45)	Pose: 56 S:(-0.46)	Pose: 88 S: (-6.65)	OP	Pose: 136 S: (-7.56)	**Pose: 163 S: (-8.07)**	G
1	**Chy5[b]**	14	Pose: 11 S: (-4.99)	Pose: 44 S: (-4.95)	Pose: 74 S: (-5.06)	NDP	Pose: 115 S: (-6.00)	**Pose: 139 S: (-5.95)**	NDP	G and P
**B. Inverted lysB-D29 orientation**										
1	**SWU-1[b]**	13	Pose: 10 S: (-5.3)	Pose: 24 S: (-6.3)	Pose: 54 S: (-6.8)	Pose: 89 S: (-6.7)	Pose: 114 S: (-7.0)	Pose: 155 S: (-6.7)	**Pose: 183 S: (-6.9)**	GNP
2	Echild	13	NDP	NDP	NDP	NDP	Pose: 101 S: (-7.31)	OP	OP	-------
3	RedRock	13	NDP	NDP	NDP	NDP	NDP	Pose: 11 S: (-7.2)	NDP	GNP
4	Sheen	12	NDP	NDP	NDP	NDP	Pose: 87 S: (-5.76)	Pose: 105 S: (-6.23)	Pose: 138 S: (-6.12)	G and P
5	Timshel	12	NDP	NDP	Pose: 32 S:(-6.14)	Pose: 58 S: (-5.26)	Pose: 79 S: (-5.98)	NDP	Pose: 134 S: (-6.43)	G and P
6	**BabyRay[b]**	9	NDP	NDP	NDP	NDP	NDP	**NDP**	NDP	-------
7	Ardmore	10	NDP	NDP	NDP	NDP	NDP	OP	NDP	G
8	Ms6	10	NDP	NDP	NDP	NDP	NDP	**OP**	NDP	G
9	**Pumpkin[b]**	18	NDP	NDP	NDP	NDP	NDP	NDP	**NDP**	GNP

10	MrMagoo	14	Pose: 8<br>S:(-5.32)	NDP	NDP	NDP	OP	Pose: 174<br>S: (-4.29)	OP	-------
11	Dylan	10	NDP	NDP	NDP	NDP	NDP	NDP	NDP	GNP
12	**Palestino**[b]	14	NDP	NDP	NDP	NDP	OP	NDP	**OP**	GNP
13	**Obama12**[b]	19	Pose: 7<br>S:(-5.14)	Pose: 27<br>S:(-5.94)	OP	OP	OP	**Pose: 160<br>S: (-6.56)**	OP	GNP
14	Larva	15	NDP	NDP	NDP	NDP	NDP	NDP	NDP	G
15	**Enkosi**[b]	16	OP	OP	OP	OP	OP	**Pose: 147<br>S: (-6.6)**	OP	-------
**II. Deep Funnel conformation<br>A. Very deep funnel**									
1	Zakai	10	OP	OP	NDP	NDP	OP	NDP	NDP	G
2	**Saal**[b]	10	Pose: 22<br>S:(-3.93)	Pose: 29<br>S:(-5.19)	OP	Pose: 97<br>S: (-6.5)	OP	**OP**	OP	GNP
3	Graduation	10	NDP	Pose: 41<br>S: (5.74)	NDP	NDP	Pose: 126<br>S: (5.94)	OP	NDP	-------
4	**DS6A**[b]	11	OP	OP	OP	OP	OP	OP	**OP**	G
**B. Less deep funnel**									
1	Twister	9	OP	OP	OP	OP	OP	OP	OP	G and P
2	Wildcat	9	OP	OP	NDP	OP	OP	OP	OP	G and P
3	**Hades**[b]	11	NDP	NDP	NDP	NDP	**OP**	OP	NDP	G
**III. Superficial funnel conformation**									
1	HelDan	7	NDP	NDP	NDP	NDP	NDP	NDP	NDP	G
2	BTCU-1	6	NDP	OP	NDP	NDP	NDP	NDP	NDP	G and P
3	**Anubis**[b]	6	Pose: 9<br>S:(-4.39)	NDP	Pose: 61<br>S:(-5.46)	Pose: 104<br>S: (-4.76)	**Pose: 118 S:<br>(-5.5)**	NDP	NDP	G and P
4	Bxz2	7	NDP	NDP	NDP	**OP**	OP	NDP	NDP	-------
5	Severus	7	NDP	NDP	OP	OP	NDP	NDP	NDP	G and P

**IV. Long Shallow bowl conformation**										
1	**Omega**[b]	16	NDP	NDP	NDP	NDP	NDP	**NDP**	NDP	G and P
**V. Narrow buried cave conformation**										
1	**Goose**[b]	9	NDP	NDP	**OP**	NDP	NDP	NDP	NDP	G and P

[a] length of loop-5 according to the 3D structural alignment of each LysB 3D model to LysB-D29 crystal structure.

[b] Representative members chosen for subsequent cloning, production and enzymatic assays.

Pose: ligand-protein conformation achieving H-bond between the catalytic Ser & ligand's ester bond (C=O).

S: (Docking score) a value calculated by MOE and indicate final energy for the ligand-substrate complex (the higher the negative value, the better the docking score).

OP: (Orientation pose) ligand-protein conformation where the catalytic Ser faces ester ligand's ester bond (C=O) with no H-bond formation.

NDP: (No Detected Pose) neither binding nor orientation pose.

Table S4: Quantitative measurements and structural alignments of LysB active sites to their counterparts of the α/β hydrolases.

No.	Item	Active site Conformation	size	No. of residues forming the active site	Volume of the active site (A^3)	Solvent Accessible Surface area (SASA) of the active site (A^2)	RMSD of alignment	Percentage of Sequence identity	TM-score
Std.	*Candida rugosa* lipase	Tunnel	149	38	903.3	455.9	0	0%	0.0526
Std.	LysB-D29		172	43	1838.5	1965.8	------	------	------
1	LysB-Chy5		78	24	499.5	572.8	0.111	100%	0.7787
2	LysB-SWU-1		142	34	1176.5	1063.6	0	100%	0.9706
3	LysB-Echild-2		82	25	902.3	1346.5	0.286	44.44%	0.04
4	LysB-Redrock		26	14	103.3	93.1	0	0%	0.0022
5	LysB-Sheen		79	22	732.4	814.6	0.878	66.67%	0.0841
6	LysB-Timshel		93	25	602.1	592.7	0.257	80.0000%	0.04
7	LysB-Babyray		116	30	893.9	1177.1	1.706	68.7500%	0.1467
8	LysB-Ardmore		125	31	530.4	859.6	0.03	33.3300%	0.0645
9	LysB-Ms6		125	31	540.9	884.4	0.03	33.3300%	0.0645
10	LysB-Pumpkin		90	32	664.6	1137.2	0	0%	0.0624
11	LysB-Mrmagoo		139	37	848.4	1081.5	0.555	69.2300%	0.0645
12	LysB-Dylan		88	28	331.1	574.5	0.199	75.0000%	0.0614
13	LysB-Palestino		218	47	1863.1	2201.1	0.875	63.6400%	0.0293
14	LysB-Obama12		147	32	1072.1	1364.5	0.476	66.6700%	0.028
15	LysB-Larva		130	29	818	1299.2	0	0%	0.0697
16	LysB-Enkosi		119	28	635.6	885.2	1.394	50.0000%	0.1235
Std.	*Candida antartica* lipase B	Very deep funnel	123	34	838.5	809.2	------	------	------
1	LysB-Zakai		74	21	459.5	366.6	0	0%	0.0707

2	LysB-Saal		186	43	957.8	1461.8	0.303	0%	0.0464
3	LysB-Graduation		77	22	239.5	389	0.755	0%	0.0455
4	LysB-DS6A		76	23	237.6	427.5	0.011	33%	N.D
Std.	*Pseudomonas cepacia* lipase	Less deep funnel	128	36	1235.9	947.2	------	------	------
1	LysB-Twister		20	9	71.1	125.7	0	0%	N.D
2	LysB-Wildcat		74	24	437.9	559.6	0	0%	0.0417
3	LysB-Hades		61	19	329.1	437.8	0	0%	0.0802
Std.	Human pancreatic lipase	Superficial funnel	41	9	157.6	282.7	------	------	------
Std.	*Fusarium solani* cutinase		67	23	421	910.2	------	------	------
1	LysB-Heldan		60	17	304.8	638.9	0	0%	0.0588
2	LysB-BTCU-1		108	32	891.9	988.8	0	0%	0.0149
3	LysB-Anubis		113	30	797.3	728.4	0	0%	N.D
4	LysB-BXZ2		91	24	661	1029.7	0	0%	N.D
5	LysB-Severus		67	19	307.1	272.2	0	0%	N.D
Std.	*Rhizomucor miehei* lipase	Long Shallow bowl	86	23	493.5	738.2	------	------	------
1	LysB-Omega		92	28	814.8	819.4	0	0%	0.0536
Std.	Acetylcholine esterase	Narrow buried cave	151	38	867.6	766.5	------	------	------
1	LysB-Goose		139	36	1342.1	1204	0.911	0.00%	0.0573

Std: Standard.
N.D: Not defined
Note: TM-score is a scoring function to assess the similarity of protein structures based on statistics [1, 2]:
0.0 < TM-score < 0.17, random structural similarity.
0.5 < TM-score < 1.00, in about the same fold.
Note: Alignment of the active site of LysB-D29 was done to *C. rugosa* lipase active site, while alignments of other tunnel-shaped LysB active sites were done to LysB-D29 active site and alignments of the other classes of LysB active sites were done to each corresponding of the class standard.

References

1. Zhang, Y.; Skolnick, J. Scoring function for automated assessment of protein structure template quality. *Proteins*. **2004**, *57*, 702-710.
2. Xu, J.; Zhang, Y. How significant is a protein structure similarity with TM-score= 0.5?. *Bioinformatics* **2010**, *26*, 889-895.

Table S5: Specific activities and kinetic parameters of LysB enzymes in comparison with relative cutinases and lipases.

Organism	Enzyme	Specific activity (U. mg^{-1})[a]			V_{max} (U. mg^{-1})			K_m (μM)			Reference
		p-NPB	*p*-NPL	*p*-NPP	*p*-NPB	*p*-NPL	*p*-NPP	*p*-NPB	*p*-NPL	*p*-NPP	
**Mycobacteriophages**											
D29	Endolysin B (LysB)	0.94±0.002	4.8±0.09	0.55±0.07	122.3	7.55	2.73	422.6	19.6	37.7	Present study - [1]
		1.61±0.031[b]	1.4±0.016[b]	0.07[b]±0.005							
Omega		N.D	9.7±0.3	0.32±0.02	111.8	79.8	1.45	618.8	280.8	24.4	
Saal		N.D	0.137±0.02	0.25±0.02	1.49	0.425	0.4	4172.12	1476.4	2833.32	
Obama12		N.D	0.018±0.004	0.0048±0.005	0.47	0.17	0.084	1268.5	3000	800	
SWU-1		N.D	0.09±0.01	0.007±0.0009							
Babyray		N.D	0.02±0.001	0.002±0.003							
Palestino		N.D	0.01±0.001	0							
Bxz2		2.7	0	N.D	49.8±1.1	N.D	N.D	211.2±4.8	N.D	N.D	[2]
Ms6		0.35	0.21	N.D	1.7±0.4	N.D	N.D	107.7±27.0	N.D	N.D	
Fusarium solani	Cutinase I				1x10^{-6}	0.008x10^{-6}	N.D	3.5x10^{7}	5.6x10^{7}	N.D	[3]
	Cutinase II				1.2x10^{-6}	0.002x10^{-6}	N.D	7.5x10^{7}	4.5x10^{7}	N.D	
								350±20[a]	N.D	N.D	[4]
					0.638 ±0.024[a]	N.D	N.D	0.121 ±0.009[a]	N.D	N.D	[5]
								1.36±0.68	N.D	N.D	[6]
								1.26 ± 0.28	N.D	N.D	[7]
	Free cutinase				6.2 x 10^{-6}	N.D	N.D				[8]
Humicola insolens	Cutinase							454±29	N.D	2246±347	[9]
								2.46±0.15	N.D	N.D	[6]
		367.6±7.6	N.D	N.D							[10]
Pseudomonas cepacia	lipase				N.D	N.D	430±2	N.D	N.D	11x10^{3}±0.8	[11]
					N.D	N.D	434±3	N.D	N.D	0.12x10^{3}	[12]

										± 0.5	
		N.D	N.D	14200 ±1420							[13]
	Wild type	3.5 x10^3 ±0.016	N.D	7.9x10^3 ±0.1							[14]
Candida rugosa lipase	free enzyme				N.D	N.D	1928.66	N.D	N.D	0.17	[15]
	LIP1	18±0.7	31±2.0	10±0.2							[16]
	LIP3	113±1.9	251±11	111±2.7							[17]
	LIP4	0.0029 ±0.0002	0.0044 ±0.0002	0.0061 ±0.0001							[18]
	lipase A	2.45	14.4	7.9							[19]
	lipase B	8.61	30.9	16.9							
	lipase A	350[b]	0[b]	0[b]							
	lipase B	146[b]	0[b]	0[b]							

[a] One unit (U) of enzyme activity corresponds to the liberation of 1 μmol of *p*-nitrophenol per min under the assay conditions.
[b] The assay was performed without addition of triton X-100 as a surfactant.
N.D: Not defined.

References

1. Abouhmad A.; Korany A. H.; Grey C.; Dishisha T.; Hatti–Kaul R. Exploring the enzymatic and antibacterial activities of novel Mycobacteriophage Lysin B (LysB) enzymes. (Manuscript in preparation)
2. Grover, N.; Paskaleva, E.E.; Mehta, K.K.; Dordick, J.S.; Kane, R.S. Growth inhibition of *Mycobacterium smegmatis* by mycobacteriophage-derived enzymes. *Enzyme Microb. Tech.* **2014**, *63*, 1-6, doi:10.1016/j.enzmictec.2014.04.018
3. Purdy, R. E.; Kolattukudy, P. E. Hydrolysis of plant cuticle by plant pathogens. Properties of cutinase I, cutinase II, and a nonspecific esterase isolated from *Fusarium solani pisi*. *Biochemistry* **1975**, *14*, 2832-2840.
4. Brissos, V.; Melo, E. P.; Martinho, J. M. G.; Cabral, J. M. S. Biochemical and structural characterisation of cutinase mutants in the presence of the anionic surfactant AOT. *Biochim. Biophys. Acta - Proteins Proteomics*. **2008**, *1784*, 1326-1334.
5. Pedersen, S.; Nesgaard, L.; Baptista, R. P.; Melo, E. P.; Kristensen, S. R.; Otzen, D. E. pH-dependent aggregation of cutinase is efficiently suppressed by 1, 8-ANS. *Biopolymers* **2006**, *83*, 619-629.
6. Baker, P. J.; Poultney, C.; Liu, Z.; Gross, R.; Montclare, J. K. Identification and comparison of cutinases for synthetic polyester degradation. *App. Microbiol. Biot.* **2012**, *93*, 229-240.
7. Liu, Z.; Gosser, Y.; Baker, P. J.; Ravee, Y.; Lu, Z.; Alemu, G.; Montclare, J. K. Structural and functional studies of *Aspergillus oryzae* cutinase: enhanced thermostability and hydrolytic activity of synthetic ester and polyester degradation. *J. Am. Chem. Soc.* **2009**, *131*, 15711-15716.

8. Stavila, E.; Arsyi, R. Z.; Petrovic, D. M.; Loos, K. *Fusarium solani pisi* cutinase-catalyzed synthesis of polyamides. *Eur. Polym. J.* **2013**, *49*, 834-842.
9. Kazenwadel, C.; Eiben, S.; Maurer, S.; Beuttler, H.; Wetzl, D.; Hauer, B.; Koschorreck, K. Thiol-functionalization of acrylic ester monomers catalyzed by immobilized *Humicola insolens* cutinase. *Enzyme. Microb. Tech.* **2012**, *51*, 9-15.
10. Carniel, A.; Valoni, É.; Junior, J. N.; da Conceição Gomes, A.; de Castro, A. M. Lipase from *Candida antarctica* (CALB) and cutinase from Humicola insolens act synergistically for PET hydrolysis to terephthalic acid. *Process Biochem.* **2017**, *59*, 84-90.
11. Dalal, S.; Singh, P. K.; Raghava, S.; Rawat, S.; Gupta, M. N. Purification and properties of the alkaline lipase from *Burkholderia cepacia* ATCC 25609. *Biotechnol. Appl. Bioc.* **2008**, *51*, 23-31.
12. Singh, P. K.; Gupta, M. N. Simultaneous refolding and purification of a recombinant lipase with an intein tag by affinity precipitation with chitosan. *Biochim. Biophys. Acta - Proteins Proteomics*. **2008**, *1784*, 1825-1829.
13. Pencreac'h, G.; Baratti, J. C. Hydrolysis of *p*-nitrophenyl palmitate in n-heptane by the *Pseudomonas cepacia* lipase: a simple test for the determination of lipase activity in organic media. *Enzyme Microb. Tech*. **1996**, *18*, 417-422.
14. Yang, J.; Koga, Y.; Nakano, H.; Yamane, T. Modifying the chain-length selectivity of the lipase from *Burkholderia cepacia* KWI-56 through in vitro combinatorial mutagenesis in the substrate-binding site. *Protein Eng.* **2002**, *15*, 147-152, doi:DOI 10.1093/protein/15.2.147.
15. Miranda, M.; Urioste, D.; Andrade Souza, L. T.; Mendes, A. A.; de Castro, H. F. Assessment of the morphological, biochemical, and kinetic properties for *Candida rugosa* lipase immobilized on hydrous niobium oxide to be used in the biodiesel synthesis. *Enzyme res.* **2011**, 216435. doi: 10.4061/2011/216435.
16. Chang, S. W.; Lee, G. C.; Shaw, J. F. Codon optimization of *Candida rugosa* lip 1 gene for improving expression in *Pichia pastoris* and biochemical characterization of the purified recombinant LIP1 lipase. *J. Agr. Food chem.* **2006**, *54*, 815-822.
17. Chang, S. W.; Lee, G. C.; Shaw, J. F. Efficient production of active recombinant *Candida rugosa* LIP3 lipase in *Pichia pastoris* and biochemical characterization of the purified enzyme. *J. Agr. Food chem.* **2006**, *54*, 5831-5838.
18. Lee, L. C.; Chen, Y. T.; Yen, C. C.; Chiang, T. C. Y.; Tang, S. J.; Lee, G. C.; Shaw, J. F. Altering the substrate specificity of *Candida rugosa* LIP4 by engineering the substrate-binding sites. *J. Agr. Food chem.* **2007**, *55*, 5103-5108.
19. Redondo, O.; Herrero, A.; Bello, J. F.; Roig, M. G.; Calvo, M. V, Plou, F. J.; Burguillo, F. J. Comparative kinetic study of lipases A and B from *Candida rugosa* in the hydrolysis of lipid *p*-nitrophenyl esters in mixed micelles with Triton X-100. *Biochim. Biophys. Acta (BBA)-General Subj.* **1995**, *1243*, 15-24.
